# Epigenomic and Other Evidence for Cannabis-Induced Aging Contextualized in a Synthetic Epidemiologic Overview of Cannabinoid-Related Teratogenesis and Cannabinoid-Related Carcinogenesis

**DOI:** 10.3390/ijerph192416721

**Published:** 2022-12-13

**Authors:** Albert Stuart Reece, Gary Kenneth Hulse

**Affiliations:** 1 Division of Psychiatry, University of Western Australia, Crawley, WA 6009, Australia; 2 School of Medical and Health Sciences, Edith Cowan University, Joondalup, WA 6027, Australia

**Keywords:** cannabis, genotoxicity, epigenotoxicity, aging, ageing, teratology, DNA methylation

## Abstract

Background: Twelve separate streams of empirical data make a strong case for cannabis-induced accelerated aging including hormonal, mitochondriopathic, cardiovascular, hepatotoxic, immunological, genotoxic, epigenotoxic, disruption of chromosomal physiology, congenital anomalies, cancers including inheritable tumorigenesis, telomerase inhibition and elevated mortality. Methods: Results from a recently published longitudinal epigenomic screen were analyzed with regard to the results of recent large epidemiological studies of the causal impacts of cannabis. We also integrate theoretical syntheses with prior studies into these combined epigenomic and epidemiological results. Results: Cannabis dependence not only recapitulates many of the key features of aging, but is characterized by both age-defining and age-generating illnesses including immunomodulation, hepatic inflammation, many psychiatric syndromes with a neuroinflammatory basis, genotoxicity and epigenotoxicity. DNA breaks, chromosomal breakage-fusion-bridge morphologies and likely cycles, and altered intergenerational DNA methylation and disruption of both the histone and tubulin codes in the context of increased clinical congenital anomalies, cancers and heritable tumors imply widespread disruption of the genome and epigenome. Modern epigenomic clocks indicate that, in cannabis-dependent patients, cannabis advances cellular DNA methylation age by 25–30% at age 30 years. Data have implications not only for somatic but also stem cell and germ line tissues including post-fertilization zygotes. This effect is likely increases with the square of chronological age. Conclusion: Recent epigenomic studies of cannabis exposure provide many explanations for the broad spectrum of cannabis-related teratogenicity and carcinogenicity and appear to account for many epidemiologically observed findings. Further research is indicated on the role of cannabinoids in the aging process both developmentally and longitudinally, from stem cell to germ cell to blastocystoids to embryoid bodies and beyond.

## 1. Introduction

Aging is the ubiquitous fate of biota and involves progressive loss of function [1]. Whilst the unkempt appearance and often poor physical and/or mental health of the patient chronically dependent on drugs including cannabis is widely appreciated, formal studies of cellular aging following chronic drug exposure are curiously absent from the literature. Major recent advances in various fields including epigenomics, epidemiology, stem-cell physiology and the mechanics of mitotic and meiotic cell division provide a unique opportunity to conduct an investigative review of the interaction of cannabis exposure and aging with a view to stimulating formal investigation of the field with epigenomic and other aging biomarkers.

Whilst teratology and cancerogenesis are well recognized aspects of genotoxicity and are now well documented in relation to cannabis-related genotoxicity, accelerated aging is the third well recognized aspect of genotoxicity generally [2], which presently lacks a detailed, coordinated and comprehensive review of its phenomenology and underlying theoretical and mechanistic bases with regard to cannabis and cannabinoids. The present paper addresses this gap.

Major hallmarks of biological aging include genomic instability, epigenomic alterations, telomere attrition, cellular senescence, mitochondrial dysfunction, altered intercellular communication, stem-cell exhaustion, difficulty with nutrient utilization and loss of proteostasis [1,3,4,5,6,7]. It is important to note that most of these pathways are now known to interact with the epigenome. Another frequently cited theory of aging is the free oxygen radical theory. Oxyradicals have also been shown to interact with epigenomic pathways via P16INK4A [8].

In 1942, Conrad Waddington hypothesized that epigenomic states constrained cell lineage differentiation to certain “valleys” so that cell specification within the major types was energetically constrained [9]. This profound insight had several implications including that differentiated cells do not readily transdifferentiate into a different cell type. Moreover, cells usually differentiate from a less differentiated progenitor state into a more highly differentiated state so that the biological age of cells in terms of numbers of cell divisions is encoded and recorded epigenetically, together with many other immune, metabolic and in neurological tissues, electrical, memories [10]. This usual process of differentiation from multipotent progenitors into progeny with increasingly restricted fate is known as canalization [11].

In 2006, Takahashi and Yamanaka screened 20 putative stem-cell factors to define the minimal signaling core group required to induce and maintain pluripotent stem cells. The four factors they defined were called OSKM factors (Oct3/4, Sox2, Klf4 and cMyc) or simply Yamanaka factors [12]. These authors used these factors to induce mouse fibroblasts to dedifferentiate back into embryonic stem cells thereby showing that the biological clock could be reversed. Elegant studies by other groups with Yamanaka factors or similar have since replicated these findings in other systems including recovery of aged rodent pancreatic islets and skeletal muscle crush [13], recovery of cardiac function and reversal of heart failure after rodent myocardial infarction [14], and recovery of vision after traumatic optic nerve crush injury, glaucoma, cataract and age-related blindness in old rats [15]. They were even able to restore and rejuvenate the aged cells of a mouse model of progeria [13]. Not only does this collection of studies generalize the Yamanaka findings relating to tissue and organismal age reversal, but, as observed by leading aging researchers, they also provide powerful evidence for the primacy of epigenomic regulation of the aging process overall [16]. In this context, the various hallmarks of aging mentioned above are now probably best understood from their relationship to the complex and multi-layered epigenomic regulatory pathways.

Cannabis dependence is defined as the state which exists when individuals become physically or mentally unwell after ceasing exposure to cannabis [17]. Cannabis withdrawal is characterized by a spectrum of symptoms including anxiety, irritability, dysphoria, craving, sleeping difficulties, abdominal cramps, muscle aches and diarrhea [17]. Chronic exposure may be defined as exposure which occurs during a period exceeding six months [17]. Daily cannabis exposure is operationally defined as being cannabis exposure on all or most days each month or twenty or more days per month [18].

Chronic cannabis dependence is characterized by many of the age defining hallmarks mentioned above with DNA breaks, fusions and bridges well described [19,20,21,22,23,24] and potentially including the breakage-fusion-bridge cycle (where chromosomal breaks lead to aberrant interchromosomal joinings and which causes chromosomal bridges to form when the chromosomes separate in anaphase which then leads to further breaks when the chromosomes are pulled apart in telophase so that the cycle repeats) [25]; major changes in DNA methylation [26,27] which have been shown to be transmissible to sperm and to a subsequent generation of offspring [26,27,28,29,30,31,32]; telomere attrition [33,34]; immunomodulation including heritable immunomodulation [35,36,37,38,39,40]; inhibition of mitochondrial function including increased free radical generation [41,42,43,44]; impairment of DNA, RNA and protein synthesis and cell growth [45,46,47,48,49] and thus stem-cell impairment and widespread negative trophic and functional effects in many tissues [47,48,50,51]. Chronic exposure is also associated with increased rates of many cancers [52,53,54,55,56,57,58,59,60,61,62,63,64,65,66]; a suppressed endocrine state [67,68,69,70,71,72,73] and impaired male and female fertility [67,71,74]. Thus, significant long-term exposure to cannabinoids recapitulates and accelerates many of the significant features of physiological aging.

The most prominent of the various biophysiological clocks which have been described to measure biological as compared to chronological aging are epigenomic clocks based on DNA methylation [75,76,77,78]. Cardiovascular [79,80,81,82], immunological [83], transcriptomic, microRNA, proteomic and metabolomic clocks have also been released [84,85,86,87,88,89,90].

### 1.1. Key Definitions

Genomic instability is a major mechanism in cancer, congenital anomalies, neurodevelopmental defects and aging. Genomic instability refers to cellular mutations and includes changes to the nucleic acid sequence, chromosomal rearrangements aneuploidy, copy number variations, circular DNA and microchromsomes [91,92,93,94,95,96,97,98,99,100,101,102].

“Canalization” refers to the process described in Waddington’s famous theory of cellular differentiation like a marble rolling down a landscape of hills and valleys and finding its energetically most favorable point, progressively becoming more terminally differentiated [9].

“Yamanaka factors” are those four cellular transcription factors originally described by Yamanaka and colleagues for potentiate the de-differentiation of terminally differentiated fibroblasts into induced pluripotential stem cells. The four factors identified were Oct3/4 (POU5F1, POU Class 5 Homeobox 1), Sox2 (SRY-Box transcription factor 2), c-Myc (MYC protooncogene, BHLH Transcription Factor), and Klf4 (KLF Transcription factor 4).

“Epigenomic regulatory pathways” refer to the many mechanisms of gene regulation including: DNA methylation, post-translational histone modifications, micro-RNAs, long non-coding RNAs, involvement in topologically defined domains and adjacency to transcription factories, closeness to the nuclear envelope (which suppresses gene transcription), involvement tin euchromatin or heterochromatin structure (the former promoting and the latter suppressing transcription), various post-transcriptional modifications of RNA including C6-adenosyl methylation, circular DNA structure and microchromsomes, amongst others.

### 1.2. Outline

The plan of this review is as follows. Firstly, twelve independent streams of empirical data for accelerated aging will be presented to make a strong case for accelerated biological aging associated with chronic cannabis exposure to set the context for the following discussion. Secondly, evidence for perturbation of some fundamental cellular machinery by cannabis exposure and withdrawal will be presented including alteration of the epigenomic machinery itself, modulation of various stem-cell factors and epigenomic interference with the chromosomal machinery of cell division. Epigenetic changes in brain and cardiovascular function are briefly considered as changes in these organs not only reflect but drive systemic aging, i.e., they are not only age-defining illnesses but also age-generating disorders. Thirdly, since cancer and congenital anomalies (birth defects) are both age-related disorders and are clinical reflections of genotoxicity and/or epigenotoxicity and are heightened after cannabis exposure [25,66,103,104,105,106,107,108,109,110,111,112,113,114,115,116,117,118], contemporary USA and European epidemiological findings are reviewed and form the backdrop for a contextual exploration of the recent powerful longitudinal epigenomic data published by Schrott and Murphy and colleagues on changes in the DNA methylome of human sperm after cannabis exposure and withdrawal annotated for many benign and malignant conditions [27]. These datasets are augmented by other recent organ specific studies highlighting genes of particular interest which are then interrogated in the Schrott data. Consideration is also given to genotoxic effects of cannabinoids more broadly including cannabidiol and Δ8-tetrahydrocannabinol (Δ8THC).

These matters are set out in tabular form in Table 1.

It is concluded that these metrics collectively point towards cannabinoid-exposed tissues being of advanced biological age resulting in age related morbidity, and that this process is driven by cannabinoid-disruption of the human epigenome, with increasing global cannabis exposure to a much greater extent than is commonly realized [119], having far-reaching public health implications for the current and future generations

## 2. Methods

Literature Review. Evidence was overviewed from the authors prior knowledge of studies examining cannabis effects on mechanisms of ageing. A literature search was conducted of PubMed on 30 November 2022 using the two sets of search terms “cannabis AND aging” and “cannabinoids AND aging”. Identified articles were manually searched. In total, 48 and 108 articles were identified from the raw searches. However, these dealt generally with only specific organ systems of aging (such as Alzheimer’s disease or pancreatic aging) and not the whole field of the pathobiology of aging itself; or alternatively hypothesized about unproven aging preventative actions. Thus, it was not possible to identify any recent reviews of the impacts of cannabis or cannabinoids on the fundamental pathobiology of aging. This finding formally demonstrates the novelty of the present study.

The 12 streams of evidence referenced flow from cellular systems and mechanisms (Epigenomic Overview) through organ systems (Prefrontal Cortex and Brain), to health disorders including cancer (Carcinogenesis), to population impacts (on birth defects and cancer).

Data. Data on rates of congenital anomalies are taken from published reports in USA [103] and Europe [115,120]. Data on cancer rates are taken from published reports on USA [112,113,114,121] and Europe [121,122]. Epigenomic DNA methylation data were taken from the EWAS (Epigenome Wide Association Study) report of Schrott and colleagues relating to cannabis dependence and withdrawal in human sperm before and 11 weeks after a period of cannabis dependence [27]. Genes of interest were searched in the 359-page pdf document which comprises the supplementary Schrott database.

Analysis. Statistical processing of code to derive relevant descriptive statistics was performed in R Studio 1.4.1717 based on R version 4.1.1 and both data and code are available as supplementary files in the following Mendeley repository https://data.mendeley.com/datasets/sngdkpg8gy/1 (doi:10.17632/sngdkpg8gy.1) (accessed on 10 December 2022. Full address is: https://data.mendeley.com/datasets/sngdkpg8gy).

Ethics. Ethical approval for this study was provided from the Human Research Ethics Committee of the University of Western Australia number RA/4/20/4724 on 24 September 2021.

## 3. Results and Discussion

### 3.1. Streams of Evidence for Cannabinoid Acceleration of Aging

Twelve independent empirical data streams both independently and collectively indicate accelerated biological aging associated with chronic cannabis exposure.

#### 3.1.1. Clinical Syndromes

Long-term cannabis dependence is characterized by a cluster of syndromes which are themselves age defining illnesses including: neuroinflammation from the many mental illnesses [123,124,125,126,127,128,129,130,131,132]; steatohepatitis and cirrhosis progression [133,134,135,136]; myocardial infarction, cerebrovascular disorders and cardiac arrythmia [17,137,138,139]; immunomodulation [35,36,37,38,39]; endocrine suppression [67,68,69,70,71,72,73]; impaired male and female fertility [67,71,74]; cancers [52,53,54,55,56,57,58,59,60,61,62,63,64,65,66]; congenital anomalies [66,103,108,109,110,111,115,116,118]; genotoxicity including DNA breaks, telomere loss and mitotic and meiotic errors [19,33,140]; epigenotoxicity including altered DNA methylation [26,27,28,29,30,31,32] and histone physiology [141,142].

#### 3.1.2. Mitochondrial Inhibition

Mitochondrial inhibition is well described in lymphocytes, neurons, sperm, hepatocytes and oocytes following cannabis exposure [41,42,43,44,140,143,144]. Mitochondria carry all of the cannabinoid signal transduction machinery found in the plasmalemma [44,145,146,147]. Since mitochondria supply energy and epigenomic substrates to the nucleus and interact with it closely via mitohormetic and mitonuclear balance systems [148,149] metabolic inhibition implies epigenomic disruption. Mitochondrial inhibition is well established as one of the key hallmarks of aging [1,150,151,152,153,154,155,156,157,158] and implicated pathophysiological pathways include such novel mechanisms as the leakage of mitochondrial DNA into the cytosol and stimulation of innate γ-interferon-dependent immunity via the cGAS-STING pathway [156].

#### 3.1.3. DNA Methylation

Many studies have documented extensive alteration of DNA methylation following cannabis administration in both rats and humans [26,27,28,29,30,31,32,159,160]. Moreover, an elegant study has proven not only that the epigenome controls the aging process but that reversion of epigenomic age can heal traumatic optic nerve injury, glaucoma and geriatric blindness as normally only seen in neonatal life [15]. Extensive reduction in histone synthesis has been demonstrated including reduced phosphorylated and acetylated isoforms [49,141].

#### 3.1.4. Mental Illnesses

Cannabis is associated with many mental illnesses including depression, stress, anxiety, PTSD, other substance dependence, bipolar disorder, schizophrenia and suicide [123,124,125,126,127,128,129,130,131,132] all of which are characterized by neuroinflammation [161,162,163,164,165,166,167,168], which is one of the hallmarks of the aged and dementing brain [169,170,171,172]. Not only is neuroinflammation an age defining illness it is also an age causing illness as it induces systemic inflammation throughout the body (“inflamm-aging”) [4,173]. Cannabis exposure was recently shown to be causally related to all four indices of mental dysfunction (depressive symptoms, any mental illness, severe mental illness and suicidal thinking) tracked by the annual nationwide massive National Survey of Drug Use and Health in a space time and causal inferential analysis [106]. Cannabis exposure has also been linked with the development of autism-like and ADHD-like syndromes in children [117,174] in spacetime and causal inferential studies [175] and in epigenomic studies [26,159,176,177]. An extensive literature and many meta-analyses strongly connect cannabis use and the development of schizophrenia by many mechanisms [17,178,179,180,181,182,183,184,185,186,187,188,189,190,191,192,193,194,195,196].

#### 3.1.5. Cardiovascular Age

Biological age as cardiovascular physiological age has been measured directly biophysically in cannabis dependence and been found to be advanced above controls [81]. An effect size of 12% and a positive dose-response relationship (*p* < 0.002) were demonstrated.

#### 3.1.6. Endocrine Suppression

Widespread suppression of many key endocrine systems including luteinizing hormone (in males and females), testosterone, prolactin (chronic effect), growth hormone, estradiol and progesterone, Graafian follicle maturation, vasopressin and pregnancy including reduced fertility have been demonstrated in association with chronic cannabis use [67,68,69,70,71,72,73]. It has also been demonstrated in combined opioid-cannabinoid-dependent patients that the reversal of the FSH/LH ratio, a key clinical biomarker of the perimenopause, happened 20 years earlier [197]. Ovarian failure has also been shown to invariably be due to DNA damage [198]. Hormonal signals are rapidly transduced by the epigenome [199]. Hormonal failure and reproductive senescence represent age-defining and age-generating illnesses [1,157,158,200].

#### 3.1.7. Liver Inflammation

Liver inflammation, cirrhosis and cancer have also been linked with cannabinoid exposure [133,134,135,136]. In that hepatic inflammation causes systemic inflammation, insulin resistance and dysmetabolism [201], generally these are also age-defining and age-generating illnesses. Moreover, the complex multi-way interaction between dysmetabolic and immunopathic changes is increasingly being defined and emphasized [202].

#### 3.1.8. Cancer

Clinical genotoxicity is expressed as heightened rates of many cancers including liver, breast, pancreas, diverse leukemias and lymphomas, oropharyngeal, thyroid, urinary, esophageal and testicular tumors [52,53,54,55,56,57,58,59,60,61,62,63,64,65,66]. Genotoxicity is also one of the well-established key hallmarks of cellular aging [1,157,158,203].

#### 3.1.9. Inheritable Cancer

Several cannabis-related cancers occur in the pediatric age group and are therefore evidence of heritable carcinogenesis [204,205] and therefore combine both teratogenesis and malignancy in the one case. This has been found for acute myeloid and lymphoid leukemias and total pediatric cancer [65,66,104,105,206] and for rhabdomyosarcoma and neuroblastoma [207,208]. One recent survey of the cannabis-exposed DNA methylome showed 487 hits for various malignancies [27].

#### 3.1.10. Congenital Anomalies

Clinical genotoxicity is also expressed as congenital anomalies. As a majority of congenital anomalies, particularly those affecting the heart and chromosomal systems, are known to be related to parental age [209,210] the congenital anomaly rate becomes a surrogate or biomarker for biological age. Dozens of congenital anomalies have been described following prenatal or community cannabis exposure in Hawaii, Colorado, Canada, Australia, USA and Europe affecting particularly limbs, central nervous, cardiovascular, gastrointestinal, uronephrological and chromosomal systems [66,103,108,109,110,111,115,116,118]. Hundreds of positive hits were recorded on a DNA methylome screen for all the organ systems involved including mitochondria, chromosomes, microtubules, body axis and embryonic growth [27].

#### 3.1.11. Telomerase Inhibition

Cannabis inhibits the activity of telomerase one of the key enzymes controlling aging [27,211]. Telomerase reverse transcriptase (TERT) is the key enzyme tasked with maintenance of telomere length and thus chromosomal length maintenance during cell division.

#### 3.1.12. Elevated Mortality Rate

Mortality has been shown to be very elevated in cannabis users in several studies [212,213,214,215,216,217,218,219,220,221,222,223] at 30% over 30 years [223] and in another had a standardized mortality index of 14.61 (C.I. 9.21–23.19) over 14 years [222]. Whilst drug overdose, suicide and AIDS were the leading causes of death, cannabis itself predisposes to other drug use and mental illness [106,224,225,226]. Mortality is of course a hard end point for aging albeit in this context the pathway is complex.

“The sections that follow integrate cannabis ageing theories from eight pathogenetic fields”.

### 3.2. Pathogenetic Field of Interest

#### 3.2.1. Epigenomic Overview

Longitudinal epigenomic data published by Schrott and colleagues on changes in the DNA methylome of human sperm after cannabis dependence and withdrawal [27] provide an explanation for the broad spectrum of cannabis-related teratogenicity and cancerogenicity mentioned above.

Table 2 presents a re-formatted extract of the Schrott data looking at the epigenomic modulation of the key epigenomic machinery itself [27]. As shown in the Table, most of these perturbations of DNA methylation occur in introns within genes but some are in upstream presumably promoter regions and some are in downstream enhancer regions.

DNA methyltransferases 1 (DNMT1) and 3A and 3B (DNMT3A, DNMT3B) are the main enzymes which are responsible for laying down the methylation signals on DNA both from conception and in response to many signals thereafter. TET1 (ten-eleven translocase) is the main enzyme responsible for removing the methylation signals. It oxidizes the methylcytosines of CpG dinucleotides and introduces a hydroxyl group which is then oxidized in subsequent steps with the effect of removing the methylation mark. Hence the first lines of this Table show that both writing and erasing the key DNA methylation marks are disturbed by cannabis dependence or withdrawal. Here it is important to note that most habitual cannabis users go through withdrawal daily which is one of the major motivations to repeat use and making withdrawal a major and defining feature of clinical cannabis dependence [227].

UHRF1 (ubiquitin-like containing PHD and RING finger domains 1) is a key enzyme which is involved with both DNA methylation and histone modifications [228]. It recruits both DNMT1 to write DNA methylation marks and histone deacetylases which control access by the transcription machinery [228]. Its tudor-like and PHD- domains recognize and bind histone 3 trimethylated at lysine 9 (H3K9me3) and unmethylated arginine-2 (H3R2me0) and recruits chromatin proteins. Hence this enzyme is regarded as a key epigenomic hub coordinating the activities of the DNA methylation and histone regulatory systems. It regulates both the retinoblastoma gene product and the P53 damage checkpoint. Its expression levels peak in late G1 and it controls the G1/S transition of the cell cycle. It plays a key role in the regulation of pericentric chromatin and thus kinetochore function and chromosomal segregation. It is also involved in DNA repair. It is a known oncogene and has been implicated in liver cancer amongst others [229]. Hence its perturbation can be predicted to have a major effect on epigenomic regulation.

DPPA3 (Developmental PluriPotency Associated protein 3) has been shown to protect the epigenome of the oocyte from methylation [230]. Whilst DPPA3 was not identified in the spermatocyte EWAS conducted by Schrott team DPPA2 was identified as indicated.

TERT (telomerase reverse transcriptase) is a key enzyme responsible for maintaining the length of telomeres and is key to maintaining pluripotency in stem cells and germ cells and is often highly induced in cancer cells. Telomeres are protective caps on the ends of chromosomes and because some length is lost with each cell replication event they usually shorten with age. Since telomere attrition is one of the key chromosomal hallmarks of aging the regulation of telomere length is a key metric for the cellular aging clock. This important finding of cannabinoid interference with this key cellular enzyme has also been reported by others [211].

The polycomb repressive complex (Table 2) is one of the main epigenomic complexes which silence heterochromatin long term. Therefore, interference with these activities can be expected to have long-term consequences for cellular health.

SMARCA2 and SMARCA4 are SWI/SNF (SWItch/Sucrose NonFermentable) ATP-dependent modifiers of chromatin which change nucleosome position in an energy-dependent manner and therefore rearrange the genome and make new sections available for transcription. Modulation of these epigenomic controllers was recently shown to have a very positive effect in advanced castrate resistant prostate cancer which was addicted to their activities [231]. Since the SWI/SNF system is a major rearranger of chromatin perturbation of this system carries major downstream implications for cellular health. SMARCA2 and SMARCA4 (also known as Brahma, BRM and Brahma-related Gene 1, BRG1) were also recently determined to be key determinants of differentiation and canalization of precursor mesodermal cells into a cardiac fate [11].

Not only is DNA methylated but so too are histone proteins. There were 161 hits in the Schrott database for histone methyltransferases which write this mark onto the histone code (some top hits shown in Supplementary Table S1) and 199 hits for the histone demethylases which remove this mark (of which an extract is shown in Supplementary Table S2).

Histone acetylation is a key mark on histone tails. By neutralizing the charge of histone tails histone acetylation opens up chromatin and makes it available for gene transcription. This key acetylation mark is written onto the histone code by histone acetyl transferases and removed by histone deacetylases. Eleven hits in the Schrott data for each of these which were noted in both cannabis dependence and withdrawal are detailed in Supplementary Tables S3 and S4 respectively.

Thus, this brief introductory overview provides good evidence of major changes not only of the DNA methylome but of the central machinery which writes and erases and coordinates the epigenetic code on both DNA and histones. Key chromosomal areas such as the telomeres and centromeres are also impacted which thereby directly impacts processes such as aging (via accelerated telomere loss) and cellular division (via disruptions of centromere/kinetochore function).

#### 3.2.2. Stem-Cell Factors

Takahashi and Yamanaka published their seminal and ground-breaking paper on the use of four defined recombinant stem-cell factors to maintain and induce the pluripotential state of embryonic stem cells in 2006 [12]. Proof of the induced stem-cell concept was provided by their demonstration that they were able to revert mouse fibroblasts to embryonic stem cells by the use of their four defined factors OSKM. These induced embryonic stem (iPS) cells went on to contribute to viable mouse embryos after injection into blastocysts. Intermittent use of the OSKM factors, a technique known as partial reprogramming, was able both to rescue a mouse model of progeria and to dramatically accelerate injury recovery to skeletal muscle and pancreatic islets in aged mice [13] and was able to improve cardiac function after myocardial infarction in a mouse model [232]. Inducible expression of OSK in retinal ganglion cells was able to restore vision in a manner only seen in neonatal mouse pups after glaucoma, optic nerve crush and extreme age in old mice [15].

As shown in Table 3, there were 11 hits in the Schrott database for the Yamanaka stem-cell factors. The name of the Oct3/4 gene has since been changed to POU5F1. SOX2, KLF4 and MYC were positively identified but Nanog was not identified.

A modification of the Yamanaka protocol using slightly different stem-cell factors where Klf4 was replaced by Lin28 was also shown to induce iPS induction [14]. Stem-cell factors used by these researchers and also by Yamanaka were further investigated in the Schrott data with results shown in Supplementary Table S5. As there were 146 hits for Ras, 230 hits for Catenin and 185 hits for Kit in this database only a leading selection is shown in the Table. Hits for PAX7, one of the skeletal muscle master transcription factors and Lin28 are also shown at the bottom of the Table. Supplementary Table S6 provides an expanded list of some of the hits for Kit.

There is also a powerful and well documented multi-way link between immune activation, dysmetabolic changes and the aging process. For example, a recent study showed that much of the effect of calorie restriction, which has been well demonstrated to induce life extension in flies, worms and mice, when applied in humans was mediated by PLA2G7 (platelet activating factor acetyl hydrolase/phospholipase A2 group VII) [202]. PLA2G7 is found in cholesterol-rich low density lipoprotein particles and PLA2G7 oxidizes saturated lipids and activates vessel wall macrophages, lymphocytes and endothelial cells. It thereby stands at the intersection of immunity and metabolic processes.

A research group from Stanford developed a biological clock based on immune biomarkers and found that CXCR9 was the key chemokine which accounted for most of the variance they identified [83]. A sizeable literature exists around NAD (nicotinamide adenine dinucleotide) metabolism and the links between its normal dramatic age-dependent decline and the ageing process itself [233,234,235,236,237,238,239,240,241]. The key rate limiting enzyme in the NAD biosynthetic pathway is nicotinamide phosphoribosyl transferase (NAMPT) which acts as the gateway to this pathway [148]. It was therefore of interest to learn if these key immune and metabolic mediators were identified in the Schrott EWAS. The results of this investigation are shown in Supplementary Table S7. Both PLA2G7 and NAMPT were positively identified. CXCR9 was not identified but CXCR13 was found.

#### 3.2.3. Chromosomal Mechanics

During the process of cell division at the beginning of prometaphase, the nuclear membrane breaks down and what has very properly been called the “mammoth” supramolecular mitotic and meiotic machine involving the mitotic spindle begins to form [242]. The process takes place on the large scale of the whole cell cytoplasm and each of its innumerable steps are tightly regulated, carefully choreographed and finely coordinated by elegant and sophisticated mechanisms. The implication of this vastness, complexity and sophistication is that the delicate process of cell division is open to perturbation and disruption at numerous steps.

Whilst the process of cell division is well known to students of biology the world over from watching time lapsed video micrographs, it is less well known that in the human oocyte the process is highly error prone with error rates of 60–90% being reported even when young oocytes are used [243,244,245,246,247,248,249] and this error rate is known to rise sharply with age [243,244,245,246,248,249]. The bipolar alignment of the mitotic spindle with two spindle poles is critical to directing the cell to divide into two daughter cells during the subsequent anaphase separation. Whilst most species have a pair of centrioles and pericentriolar material (called centrosomes) which direct this process this is absent from higher (non-rodent) mammalian species including humans. Such species organize their spindle poles using acentriolar microtubule organizing centers (aMTOC) organized by NUMA (nuclear mitotic apparatus protein) and the kinesin motor protein KIFCI (kinesin family member C1) to draw the microtubules together [248]. Supplementation of human oocytes with KIFC1 largely rescued the high mitotic error rate [248] and in mice its knockdown via degron mediated destruction increased the error rate of bovine and modified aMTOC-free mouse oocytes to be highly similar to that of the human oocyte [248]. In actual fact, the number of poles in human oocytes mitotic spindles oscillates dynamically during oocyte maturation over several hours from several poles to just one pole and most frequently settles at just two spindle poles [245]. This implies that NUMA and KIFC1 are key to the integrity and reliability of the inherently error-prone oogenesis mitotic process in humans [248].

In worms, a kinesin-12 protein (KLP-18, kinesin-like protein), dynein (and its binding partner dynactin) and a kinesin-5 member (BMK-1, Big Mitogen Activated Protein 1) are required to prevent spindle splaying [247,250].

The anaphase-promoting complex/cyclosome (APC/C) is known to be a key organizer of the mitotic spindle and to determine when all the paired chromosomes are aligned satisfactorily on the metaphase plate and thus licences and controls the chromosomal separation of anaphase [249,251]. In human-derived HEK293 cell lines it was shown that APC/C also localizes to the centrosome where its activity is controlled by Cep152 (Centrosomal Protein 152) in complex with Cep 57 and Cep 63 [249].

Tubulin is also subject to numerous post-translational modifications particularly acetylation, polyglutaminylation and tyrosinylation [252]. Acetylation is key to the formation of the tubulin polymers of the mitotic spindle and this is controlled by lysine (K) acetyltransferase and histone deacetylases (HDAC) particularly HDAC3, HDAC6 and HDAC11 and the sirtuin (SIRT) HDAC’s SIRT2 in meiosis I and SIRT1 in meiosis II [245]. The process is also sensitive to oxidative stress and ROS (reactive oxygen species) are known to play important roles in both folliculogenesis and oocyte maturation but excessive ROS levels have been linked to shrinkage of the width and length of the mitotic spindle, disruption of the spindle asters, chromosomal misalignment in metaphase II, chromosomal disassembly in meiosis I and II and increased aneuploidy rates [245]. Adducts of ROS including 4-hydroxynonenal form and co-localize with α-, β- and γ-tubulins [245]. Ovarian ROS production also rises with age [245].

Importantly, acetylation of lysine-40 on polymerized α-tubulin by α-tubulin acetyl transferase 1 (ATAT1) occurs on the inner surface of the microtubule and allows for running repairs to be undertaken on the polymer when the microtubules is stressed or bent thereby adding greatly to the structural strength and flexibility of the structure [253]. Without K-40 alpha-tubulin acetylation, the microtubules remain brittle and bending leads to microtubule fracture and chromosomal derailment, isolation, aneuploidy and micronucleus development during the anaphase disjunction. Unlike female meiosis, cell division in the fertilized zygote is organized around centriole-containing centrosomes which are derived from the paternal gamete as those associated with the female pronucleus are rudimentary [243,244]. It is therefore clear that interference with any of these structural, binding, signaling or motor proteins will lead to an elevated error rate of human female gametogenesis [248].

Supplementary Table S8 therefore presents the hits identified in the Schrott database for NUMA, CEP and kinesin- and dynein-dynactin motor proteins. It is noted that KIF14 is an alternate nomenclature for KIFC3 which was noted to be critical [248]. Hits in intron, exon and enhancer regions are noted. There were 218 hits for kinesin motors and these hits were some of the strongest hits identified in cannabis dependency in both Schrott’s Tables S1 and S4 [27]. Some of the top-scoring kinesin motor protein hits are detailed in Supplementary Table S9. It is noted that these results for the DNA methylome come from sperm so it remains to be determined how the detailed results from oocytes might compare.

When one considers tubulins in the database of Schrott and colleagues, 106 hits are obtained. Some of those for tubulin (not including the pseudogenes) and ATAT1 are shown in Supplementary Table S10. This Table also shows epigenomic hits identified for some of the key enzymes which write and modify the tubulin code including acetylation, tyrosinylation/detyrosinylation and acetylation. In total, 86 of the hits observed for tubulin are for TUBB6 (β-tubulin 6 class V) and these appear as the most significant of all of the functional annotations in the Schrott Table S4 for cannabis dependence as partially extracted in Supplementary Table S11. TUBB6 epimutations are also linked with many cancers [27].

#### 3.2.4. Centromeres and Kinetochores

In addition to the poles, organization and microtubular rays of the mitotic and meiotic spindles the points of attachment of the chromosomes to the microtubules also form a key locus of control for the whole mitotic process and a key point of vulnerability at which xenotoxins may impact. Somewhat confusingly the combination of the central repetitive non-coding DNA at the center of the chromosome (the centromere) together with its accompanying histones and proteins is (also) referred to as the centrosome. The key marker for the development of the centromere is the substitution of histone 3 (H3) for its derivative CENPA (Centrosomal protein A) and the formation of neocentromeres can be induced by the forced expression of CENPA along chromosomal arms [254]. A multiprotein complex of 16 other centrosomal proteins called the kinetochore is then assembled on the centrosome at CENPA to form a large multimolecular complex which binds to the growing plus ends of 25–30 microtubules for each chromosome.

Detailed descriptions of the protein composition of the kinetochore have appeared [254,255,256]. When these proteins are run through the Schrott database 109 hits are obtained for the 19 proteins listed in Table 4. Interestingly, 86 of these hits are for CENPN which is the equal second protein to assemble alongside CENPA at the very commencement of kinetochore assembly. Some of the most significant hits for CENPN are shown in Supplementary Table S12 and are extracted from the Table S4 in Schrott’s dataset for cannabis dependence. They are notable for their very high levels of statistical significance along with their association with uniformly malignant disorders. With the exception of SPC24, all the hits identified were in cannabis dependence rather than cannabis withdrawal.

Table 4 also includes details on the addition of the Small Ubiquitin-like MOdifier (SUMO) protein to histones. Sumoylation is a key post-translational modification (PTM) of many proteins which has been shown to be critically involved in many key genomic functions such as DSB repair, DNA transcription and replication and chromosomal segregation and synapsis [257,258]. Sumoylation is a foundational post-translational modification on many proteins including RNA polymerase II which forms the basis for the addition of sometimes lengthy chains of PTM’s which control these key genomic activities [258]. Δ9THC acting via CB1Rs has been shown to directly modulate P53 (the “guardian of the genome”) and Mdm2 (murine double minute, one of its key controlling proteins) [259]. As documented in the lower segment of Table 4, it was demonstrated in the Schrott EWAS that SUMO1 itself, one of the key E3 SUMO ligases which attaches the PTM to proteins, ZNF451 (zinc finger 451) and two of the SUMO endopeptidase proteins (SENP6 and SENP7) which cleave the SUMO PTM’s are affected epigenomically by cannabis dependence and withdrawal.

Since centromeres form the site of attachment of the chromosomes to the mitotic spindle, it follows that centromeric stability is key to maintenance of genomic stability [2]. In fact, centromeres are intrinsically “stiffer” and more fragile than the rest of the chromosome and represent “hot spots” for double stranded break (DSB) occurrence and chromosomal rearrangements [2]. Accurate repair of these breaks by homologous recombination is therefore essential to genome stability. Homologous recombination is normally understood to be suppressed in the G1 phase of the cell cycle. However, it has recently been reported that CENPA together with its chaperone HJURP (Holliday Junction Recognition Protein) and dimethylation of H3 (H3K4me2) permit invasion of the double stranded DNA by the DNA-RNA hybrids (R-loops) and licences the assembly of the RAD51 (RAD51 Recombinase)—BRCA1 (BRCA1 DNA Repair Associated 1)—BRCA2 complex which is the core complex of the main high fidelity homologous recombination (HR) pathway. Inhibition of HR necessarily leads to activation of much lower fidelity pathways such as microhomology-mediated end joining mediated by RAD52 and compromises genomic stability [2]. These investigators were able to demonstrate that RAD51 inhibition greatly increased centromeric breaks and centromeric translocations in NIH3T3 cells (as immortalized embryonic fibroblast cell line). Inhibition of both RAD51 and RAD52 together, inhibited both major repair pathways and blocked the formation of chromosomal translocations [2].

These findings lend special significance then to the combined demonstration in Supplementary Table S13 of much greater epigenomic interference with RAD51 than RAD52 by cannabis dependence and withdrawal (9 hits vs. 1) in the Schrott data and the well documented increased rate of chromosomal translocations seen experimentally after cannabis exposure [19,20,21,22,23,24,260,261,262].

#### 3.2.5. Prefrontal Cortex and Brain

It is of interest to consider the representation in the Schrott EWAS of some of the key genes and pathways which are believed to be central to brain development. DSCAM (Down syndrome cell adhesion molecule) is most highly expressed in the fetal brain and retina where it is involved in neuronal self-avoidance, axon growth cone guidance, amacrine and retinal ganglion cell dendrite arborisation, commissural midline crossing in the spinal cord, homophilic synapse development and congenital heart disease [263,264]. It is overexpressed in Down syndrome and this has been implicated in some of the development of intellectual impairment in that disorder [264]. Supplementary Table S14 sets out the 14 EWAS hits in the Schrott database for DSCAM.

DLGAP2 (DLG associated protein) is an autism associated candidate gene also implicated in schizophrenia which has previously been linked with paternal cannabis exposure in sperm EWAS Studies [27]. It was thus of interest to see if the present study confirmed these earlier results. Supplementary Table S15 shows that indeed these results were strongly confirmed by the present EWAS series.

It was shown in the last decade that one of the main reasons for the relatively very enlarged frontal lobes of the human brain is the increased activity of Robo (Roundabout) signaling in the frontal cortex which leads to a greatly expanded neurogenesis in the frontal lobes and hyperproliferation of dedicated neural progenitor cells which feed into the exuberant frontal lobar growth [265,266,267]. Slits 1–3 form the natural ligand for robo receptors. The system is involved in both nervous system development and patterning and axonal guidance and also in arterial pathfinding and steering [209]. It has also been shown that this activity is blocked by cannabinoids [268]. It was therefore fascinating to observe that SRGAP2C (SLIT-ROBO Rho GTPase Activating Protein 2C) was identified by genomic screens and comparative genetics across many species to be the gene responsible for the exuberant outgrowth of the human forebrain neocortex [269]. Indeed, inducible expression of the forebrain of mice increased the cortical neuronal density and the synaptic short and long range corticocortical and bidirectional thalamocortical connectivity of layer 2/3 pyramidal cortical cells, enhancing their computational power and the rodents’ ability to quickly learn complex sensory-discriminant tasks [269].

For these reasons, it was of interest to observe how this system performed in the Schrott EWAS. Supplementary Table S16 sets out five results for Slits, Supplementary Table S17 sets out 26 results for Robo and Supplementary Table S18 sets out the eight results for SRGAP2C and its natural antagonist and controller SRGAP2B.

Another system which has also been shown to induce the relative overgrowth of the enlarged human forebrain is retinoic acid (RA). It was recently shown that high concentrations of RA at the frontal pole decline to lower and more normal levels at the posterior of the prefrontal neocortex in the premotor cortex [270]. The enzyme at the anterior pole which is chiefly responsible for synthesizing the high levels of RA is ALDH1A1 (aldehyde dehydrogenase 1 family member 1), the RA signal is transduced by the retinoid receptors RXRG and RARB, and RA is catabolized near the premotor cortex by CYP26B1 which is part of the cytochrome P450 system [270].

It was thus of interest to examine how these systems were affected in the Schrott EWAS. Supplementary Table S19 lists 11 hits for ALDH1 including two hits for ALDH1A1 and cadherin and protocadherin (PCDH17) which also function in this pathway. Indeed there were 156 hits for protocadherin 17 from the very lowest *p*-vale of 7.73 × 10^−20^ [27]. The nine hits for retinoid receptors are disclosed in Supplementary Table S20. Although CYP26B was not identified in the Schrott screen there were twelve hits for CYP2 series cytochromes including CYP20A1, CYP27A1, CYP27C1 and CYP27C2; and CYP2B7P, CYP2C1, CYP2C18, CYP2C61P and CYP2W1.

#### 3.2.6. Cardiovascular System

Aging of the cardiovascular system is known to be a critical determinant and driver of systemic aging [271,272,273,274,275,276]. Indeed, it is said that one is as “old as one’s arteries” [157,158,277,278,279]. This is true at both the macrovascular level, with myocardial infarction being a major cause of death in developed nations, and at the microvascular levels where capillaries and sinusoids often form critical elements of many stem-cell niches [157,278,279]. Moreover, a two-way crosstalk has recently been defined between major cardiovascular disorders (myocardial infarction, hypertension and atherosclerosis) and the bone marrow haemopoietic stem-cell niche where endothelial inflammation in one compartment directly signals to the stem-cell compartment of the other system [280,281]. For these reasons, consideration of the epigenomic findings in the Schrott cannabis exposure and withdrawal data of relevance to arterial health are central to any consideration of cannabinoid-related aging processes. A detailed consideration of the cardiovascular hits in the Schrott study is deferred until the later section on teratology (see Supplementary Table S25).

It is of interest to consider the genomic processes controlling arterial health. The key genes involved in generating arteries from embryonic angioblasts are listed as sonic hedgehog (shh), vascular endothelial growth factor (VEGF), notch and ephrin B2 [209]. These genes and pathways were therefore screened through the Schrott dataset and the hits identified in Table 5A,B were identified. PTCH1 is the main shh receptor. Gli3 (GLI family zinc finger 3) is one of the key transcription factors which mediates shh signaling in the nucleus [282]. Gli3 scored 185 hits in the Schrott EWAS data of which only a selection has been extracted for illustration. PSENEN (Presenilin enhancer, gamma secretase subunit) is a key plasmalemma bound enzyme which processes the shh ligand after receptor binding. SUFU (SUFU negative regulator of hedgehog signaling) inhibits shh [283].

Supplementary Table S21A,B list genes involved in the notch signaling pathway identified in the Schrott screen. JAG1 is a canonical notch ligand. Notch 1-3 are notch receptors. RBPJ (Recombination Signal Binding Protein for Immunoglobulin Kappa J Region) is an important transcriptional regulator of notch signaling. PSENEN also processes the notch ligand at the cell membrane [284].

Supplementary Table S22A,B list the six hits in the Schrott database relating to VEGF and EphrinB2 signaling. Both VEGF and EphrinB2 are key signaling and transduction factors involved in mediating numerous major morphogenic decisions and pathways [209,251].

In this regard, fascinating recent detailed studies have appeared on the profound impact of prenatal cannabinoid (as Δ9THC) exposure on cardiac development. Robinson and colleagues showed that prenatal exposure to Δ9THC led to cardiac wall thickening in three week old mice and thickening and hypertrophy of the semilunar valves and increased ventricular septal defects [285]. Myocardial cell proliferation was increased and cardiac function was reduced with lower ejection fraction, fractional shortening and cardiac output.

Lee and co-workers demonstrated rat fetal growth restriction following in utero exposure to Δ9THC, smaller hearts and reduced a heart to body weight ratio at birth [286]. By three weeks of post-natal life this has been reversed by post-natal catchup growth which resulted in larger but stiffer ventricular wall thickness and a corresponding reduction in cardiac output. This was linked with increased expression of collagens I and III, reduced matrix metalloproteinase 2 and increased glycogen synthase kinase 3β signaling all of which are linked with cardiac remodeling. This study is highly significant as it relates the smaller hearts at birth to subsequent cardiac stiffness and reduced cardiac output, all of which are age related changes [277]. These changes in early postnatal life are known to be causally related to increased incidence of adult heart disease in later life which is the leading cause of death globally [285,286,287].

Many congenital anomalies and cancers in USA and European epidemiological datasets have been shown to be heightened after cannabis exposure. The following sections on these cannabinoid-related teratogenic and carcinogenic findings are respectively reviewed using the epigenomic data on changes in the DNA methylome of human sperm after cannabis exposure and withdrawal with a focus on genotoxicity and/or epigenotoxicity.

#### 3.2.7. Cannabinoid-Related Teratogenesis

The consistent association between congenital anomalies and cannabis exposure provides functional examples of how cannabis ageing mechanisms contribute to inter-generational disability. Table 6 directly compares the congenital anomalies which were found to be cannabis-associated in USA [103] with those identified in recent reports in the larger European dataset [115]. In total, 45/62 congenital anomalies were found to be cannabis-associated in the US dataset compared to 89/95 in the larger European dataset [103,115]. These concerning findings are noted to be highly concordant with those of other investigators in recent large population-based series [66,107,108,109,110,111,116,118,288,289]. These data are presented to introduce and contextualize the system-based narrative discussion undertaken in the following sections.

**Table 5:** Cannabis Impacts on Sonic Hedgehog Signaling, Schrott EWAS Data.

**(A)**									
**Nearest Gene Name**	**Nearest Gene Number**	**Page No.**	**Annotation**	**Chromosome Number**	**Dependency Status**	**Relative Position**	**Distance to Nearest Gene**	***p*-Value**	**Bonferroni Adjusted *p*-Value**
PTCH1	ENSG00000185920	58	Shh Receptor	9	Dependence	Intron	0	3.46 × 10^−6^	0.012789
PTCHD1-AS	ENSG00000233067	91	lnc Promoter/enhancer	X	Dependence	Intron	0	8.61 × 10^−6^	0.019678
PTCHD1-AS	ENSG00000233067	129	lnc Promoter/enhancer	X	Withdrawal	Intron	0	8.21 × 10^−8^	0.002096
PTCHD4	ENSG00000244694	138	Shh Receptor; Otopalatodigital syndrome	6	Withdrawal	Intron	0	4.21 × 10^−7^	0.005104
PTCH1	ENSG00000185920	185	Shh Receptor	9	Withdrawal	Intron	0	5.80 × 10^−6^	0.017679
SUFU	ENSG00000161996	207	Hedgehog Inhibitor	16	Withdrawal	Exon	0	1.01 × 10^−5^	0.022942
Gli3	ENSG00000106571	78	Shh mediator	7	Dependence	Downstream	81232	6.35 × 10^−6^	0.017090
Gli3	ENSG00000106571	99	Shh mediator	7	Dependence	Intron	0	1.00 × 10^−5^	0.021181
Gli3	ENSG00000106571	124	Shh mediator	7	Withdrawal	Downstream	20318	8.23 × 10^−9^	0.000646
Gli3	ENSG00000106571	182	Shh mediator	7	Withdrawal	Intron	0	5.28 × 10^−6^	0.001687
Gli3	ENSG00000106571	231	Shh mediator	7	Withdrawal	Intron	0	1.62 × 10^−5^	0.028539
**(B)**									
**Nearest Gene Name**	**Nearest Gene Number**	**Page No.**	**Annotation**	**Chromosome Number**	**Dependency Status**	**Number Genes Identified**	**Function**	***p*-Value**	
PTCH1	ENSG00000185920	237	Notch Processing	9	KEGG Pathway	31	Notch Processing	0.044117	
PTCH1	ENSG00000185920	238	Skin cancer	9	KEGG Pathway	54	Notch Processing	0.067770	
PSENEN	ENSG00000185920	326	Cutaneous melanoma	19	Withdrawal	110	Notch Processing	0.000008	
Gli3	ENSG00000106571	325	Skin lesion	7	Withdrawal	115	Notch transcription factor	1.65 × 10^−6^	
Gli3	ENSG00000106571	325	Head and Neck SCC	7	Withdrawal	53	Notch transcription factor	3.59 × 10^−6^	
Gli3	ENSG00000106571	325	Skin cancer	7	Withdrawal	113	Notch transcription factor	4.79 × 10^−6^	
Gli3	ENSG00000106571	325	Lung adenocarcinoma	7	Withdrawal	42	Notch transcription factor	5.84 × 10^−6^	
Gli3	ENSG00000106571	325	Cancer	7	Withdrawal	149	Notch transcription factor	7.17 × 10^−6^	
Gli3	ENSG00000106571	326	Large bowel cancer	7	Withdrawal	120	Notch transcription factor	7.45 × 10^−6^	
Gli3	ENSG00000106571	326	Cutaneous melanoma	7	Withdrawal	110	Notch transcription factor	7.71 × 10^−6^	
Gli3	ENSG00000106571	326	High-grade astrocytoma	7	Withdrawal	82	Notch transcription factor	8.42 × 10^−6^	
Gli3	ENSG00000106571	326	Abdominal adenocarcinoma	7	Withdrawal	135	Notch transcription factor	8.46 × 10^−6^	
Gli3	ENSG00000106571	327	Solid cancer	7	Withdrawal	150	Notch transcription factor	9.16 × 10^−6^	
Gli3	ENSG00000106571	327	Head and Neck cancer	7	Withdrawal	137	Notch transcription factor	9.54 × 10^−6^	
Gli3	ENSG00000106571	327	Sensory development	7	Withdrawal	18	Notch transcription factor	1.30 × 10^−5^	
Gli3	ENSG00000106571	327	Carcinoma	7	Withdrawal	148	Notch transcription factor	1.38 × 10^−5^	

The *p*-values which relate to these various anomalies may be extracted from the Schrott EWAS database as indicated in Supplementary Table S23. This Table provides a list of 245 systems, targets and annotations ordered by their system for all of the above EWAS hits. The above table demonstrates cross-nationally consistent associations between cannabis exposure and varied congenital abnormalities. The sections that follow evaluates evidence associating cannabis exposure with epigenomic mechanisms for congenital abnormalities.

Supplementary Tables S24–S32 present the systems-based interrogation of the Schrott database for the cardiovascular, central nervous, face, general, limb, gastrointestinal, chromosomal, uronephrological and body wall systems respectively. Examination of these Supplementary Tables demonstrates that they offer profound insights into the possible pathogenesis of the congenital anomalies described in Table 6.

Supplementary Table S24 describes 73 central nervous system EWAS hits and lists features such as brain size, brain formation, forebrain patterning, development of many kinds of synapses, head development, head size, movement and viability of cerebral cortex cells, neurite growth, neuronal growth, neuronogenesis and neuronal outgrowth and proliferation, brain cell migration, axonogenesis and outgrowth which would be consistent not only with defects such as brain growth and size (microcephalus and anencephalus) but also defects of brain function such as epileptiform disorders, autism [117,174,288,290,291], intellectual disability (mental retardation) and many mental illnesses in childhood and later life [290,291,292,293,294,295,296,297,298,299,300]. Many disorders of eye development are also noted which is consistent with the finding of microphthalmia in both the USA and European series. Many disorders of inner ear development are noted consistent with the findings of microtia and anotia in the USA and European datasets. Associations are also reported with some malignant brain conditions which is consistent with earlier reports [59].

Supplementary Table S25 shows the 29 EWAS hits which are linked with the 23 cardiovascular anomalies in Europe and the eleven cardiovascular anomalies in USA. Hypoplasia of the cardiac chambers is mentioned both in Supplementary Table S25 and reported for both left and right ventricles in the congenital anomaly (CA) list of Table 6. Septal defects are reported in the EWAS list and in the CA list for both atria and ventricles. Anomalies of the atrioventricular valves/endocardial cushions are mentioned in the EWAS hit list and mitral and tricuspid valvular anomalies including Fallot’s teratology are mentioned in the CA teratological list. Many defects of vasculogenesis, angiogenesis, pulmonary venogenesis and vascular breakdown are mentioned on the EWAS list and the cardiovascular anomalies of transposition of the great arteries, total anomalous pulmonary venous return, vascular disruptions, VACTERL (vertebral, anal, cardiac, tracheoesophageal atresia, renal and limb) syndrome, aortic arch anomalies, coarctation of the aorta, severe cardiac congenital anomalies, double outlet right ventricle, tetralogy of Fallot and others were identified on the CA list.

Supplementary Table S26 lists 22 EWAS hits of interest for facial development. Development of the face has been shown to impact brain development embryologically as the organizers for both regions interact during gestation and both are controlled by strong anterior gradients of sonic hedgehog and retinoic acid [209]. Supplementary Table S26 lists anomalies of the head, palate, nose, lens, iris and ear which relate to listed CAs of microcephaly, cleft lip and palate (which may involve the nasolabial groove), congenital cataract (in both USA and Europe) and anotia/microtia (in both USA and Europe). Importantly the severe CA holoprosencephaly which is strongly associated with abnormal brain development was identified as a strong association of cannabis teratogenesis in Europe and a weak association in USA [103,115].

Supplementary Table S27 lists 60 hits from the Schrott EWAS dataset relating to “general” issues which do not readily classify under other systems. In total, 36 (60%) of these hits relate to cannabis dependence and 24 (40%) to cannabis withdrawal. The EWAS list provides fascinating and powerful insights to the observed teratological profile documented in Table 6. Defects of cell growth, embryonic growth, organismal growth and embryonic morphogenesis head up the Table. Defects of most major DNA activities are comprehended including synthesis, binding, recombination, transcription, translation, repair, recombination, replication, and synapsis (crossing over) are shown. Defective RNA translation is indicated. Defects of chromosomal synapsis, homologous pairing, assembly and synapsis are shown.

Mitochondrial defects are listed. This is important as mitochondria supply both the energy for genomic and epigenomic reactions and the underlying substrates for the epigenomic machinery. Two hits for microtubular impairment are shown, one each in cannabis dependence and withdrawal. This may relate to anomalous chromosomal mis-segregation disorders for chromosomal trisomies and monosomies affecting chromosomes 13, 18, 21 and X (Supplementary Table S27). Reproductive defects are indicated with diminished ovarian reserve—a hallmark of ovarian ageing—and three hits for breast cells which potentially relate to recently reported elevated rates of breast malignancy in USA in relation to cannabis consumption [66,112,113,114,121].

Anomalies of body trunk and body axis development are shown. In total, 22 anomalies of bone development are listed consistent with very elevated rates of VACTERL syndrome reported from Europe.

Supplementary Table S28 reports six hits for limb anomaly development consistent with major limb anomalies including limb reductions reported from both Europe and USA. These studies may be extended further as indicated in Table 7. It is known that morphogens such as retinoic acid, fibroblast growth factors (FGFs) and Wnts play pivotal roles in the three dimension temporally sequenced complex choreography of limb development [209]. Genes such as Meis1/2 (Meis homeobox), FGF4, RXRA (retinoid X receptor) and RARB (Retinoic Acid Receptor B), TBX4/5 (T-box transcription factor), Wnt’s, shh, GREM1/2 (Gremlin), CHD7 (Chromodomain Helicase DNA binding protein 7), TMEM107 (Transmembrane Protein 107), MEGF8 (Multiple EGF-like domains 8), BMP4, and GLI3 play key roles [27,209,301].

Some of the hits from the Schrott EWAS data are extracted and illustrated in Table 7. Numbers shown in bold on the right-hand side of the second column on the right are the total hits for that gene. The other numbers listed in the “Numbers of genes column” are the numbers of genes identified with the particular DNA methylation pattern identified and listed in the Schrott dataset. Hence Meis1 had 37 hits in the EWAS, Meis2 97 hits, FGFs 175 hits, FGF4 7 hits, RXR/RARs 10 hits, CHD7 124 hits, MEGF8 105 hits, TMEM107 232 hits, BMP4 166 hits and Gli3 183 hits. Together, this accounts for 1129 hits in these major morphogens and gene pathways which is a very substantial number of perturbations compromising limb morphogenesis.

In total, 37 gastrointestinal EWAS hits are listed in Supplementary Table S29 which relate to the many gastrointestinal congenital anomalies reported in Table 6 which affect most of the major gastrointestinal organs. 27/37 (73%) relate to cannabis dependence and 10 (27%) are in withdrawal. Supplementary Table S29 also lists most of the gastrointestinal organs. Cancer and carcinoma are prominently identified.

Supplementary Table S30 lists four Schrott EWAS hits for chromosomal disorders. Given that trisomies 13, 18 and 21, Turners, Klinefelters and genomic deletions along with all chromosomal disorders are all listed in Table 6 this is highly important. As discussed in earlier sections on the underlying subcellular pathoaetiology, it is not clear if these chromosomal disorders relate to epigenomic, microtubular, kinetochore, centrosome or related problems or possibly some combination of these aberrations.

The eight identified EWAS hits for renal disorders are shown in Supplementary Table S31. These clearly cover most aspects of uronephrological development. These relate to the many uronephrological CAs identified in Table 6 including overall urinary anomalies, multicystic renal disease, obstructive genitourinary disorder, congenital posterior urethral valve, renal agenesis, bladder extrophy and hydronephrosis. Importantly, renal agenesis was a strong association of cannabis teratogenesis in both USA and Europe. This fits with the above pathophysiological narrative as sonic hedgehog and retinoic acid are major morphogens in renal and urinary development [209].

Supplementary Table S32 lists 15 EWAS hits for body wall development. In total, 7/15 (46.7%) are in cannabis dependence and 8 (53.3%) are in cannabis withdrawal. Body trunk and body axis development are prominent as is development of the abdomen. Growth and differentiation of embryonic tissues is clearly predominant in the lower part of the Table.

These various Tables may be combined by body system as shown in Supplementary Table S33. This Table does not include the extended studies listed above for congenital limb anomalies. Supplementary Figure S1 presents the summary of the *p*-values as the negative log of the *p*-value as boxplots. Non-overlapping notches indicate statistically significant differences. Gastrointestinal, chromosomal and neurological defects appear towards the right end of the graph.

Table 8 provides the mean and median *p*-value for each system. A significantly rising trend by body system is noted (β-est. = 1.21, Student’s t = 7.65, *p* = 4.69 × 10^−13^; Adj R Squ. = 0.1908, F = 58.53, df = 1, 243, *p* = 4.69 × 10^−13^).

If one considers 39 of the (arguably) most significant target organs of interest the results for mean and median *p*-value shown in Supplementary Table S34 are revealed which are plotted graphically in Supplementary Figure S2. Gastrointestinal, liver, brain, atrioventricular valves, head and chromosomes appear towards the right-hand side of this Figure as most severely affected. Again, the trend across this graph is highly statistically significant (β-est. = 0.31, Student’s t = 9.23, *p* = 6.82 × 10^−18^; Adj R Squ. = 0.2565, F = 85.16, df = 1, 243, *p* = 6.82 × 10^−18^).

Comparison of *p*-values between dependence and withdrawal shows that those in dependence are much lower than those in withdrawal (median (log P) ± IQR: cannabis dependence −7.66 (−10.56, −6.34); cannabis withdrawal −5.96 (−7.26, −5.17); t = 6.341, df = 187.12, *p* = 1.65 × 10^−9^). These findings are illustrated graphically in the boxplot of Supplementary Figure S3.

**Figure 1:**
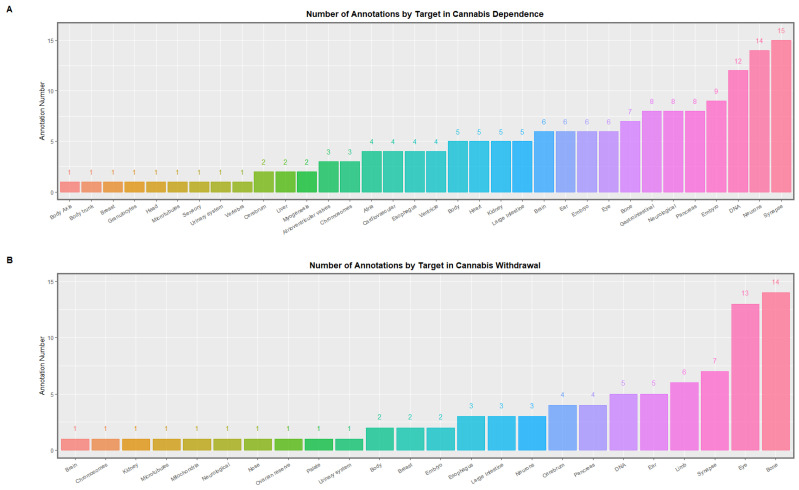
Number of epigenomic annotations in the Schrott database for target organs by dependency status in (**A**) cannabis dependence and (**B**) withdrawal.

**Figure 2:**
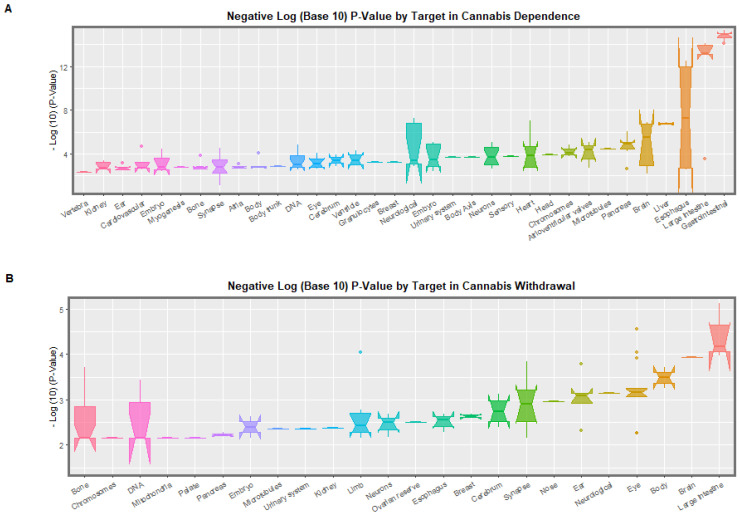
Significance levels (as *p*-values) of target organs by dependency status in (**A**) cannabis dependence and (**B**) withdrawal in the Schrott database.

These data may be summarized by target organ as shown in Table 9. The number of annotations cited in the Schrott EWAS data by target for cannabis dependence and withdrawal is shown in Figure 1. The gene numbers identified in each condition by target are shown in Supplementary Figure S4. Figure 2 compares the relative *p*-values in each condition by target organ.

#### 3.2.8. Cannabinoid-Related Carcinogenesis

The consistent association between varied cancers and cannabis exposure provides further examples of how cannabis ageing mechanisms contribute to disease. Table 10 sets out the most significant associations of various cancers with cannabis or cannabinoids in USA and Europe [112,113,114,121]. The Table lists the minimum *p*-value, the model type and the primary correlate of the various cancers listed. Two of the main features of this Table are the number of cancers listed and the commonality between the USA and European experience which are the two largest datasets on this issue available internationally.

The above table demonstrates cross-nationally consistent associations between cannabis exposure and varied cancers. The sections that follow evaluate evidence associating cannabis exposure with cancer epigenomic mechanisms.

Supplementary Table S35 extracts all of the *p*-values applicable to 20 of these tumors comprehended by the Schrott EWAS dataset. Supplementary Table S36 summarizes the data of the preceding Table for minimum, mean and median significance levels by tumor type and is ordered by descending minimum *p*-value. The cumulative gene number includes duplicate mentions for some genes. Thyroid, melanoma and urinary cancers head this list. When the list is ordered by median *p*-value thyroid, testis, stomach, liver and oropharyngeal tumors head the list (Supplementary Table S37). Some of these key data are shown in Supplementary Figure S5 which lists the number of annotations, the cumulative gene number and the negative log of the *p*-value for each tumor type.

**Figure 3:**
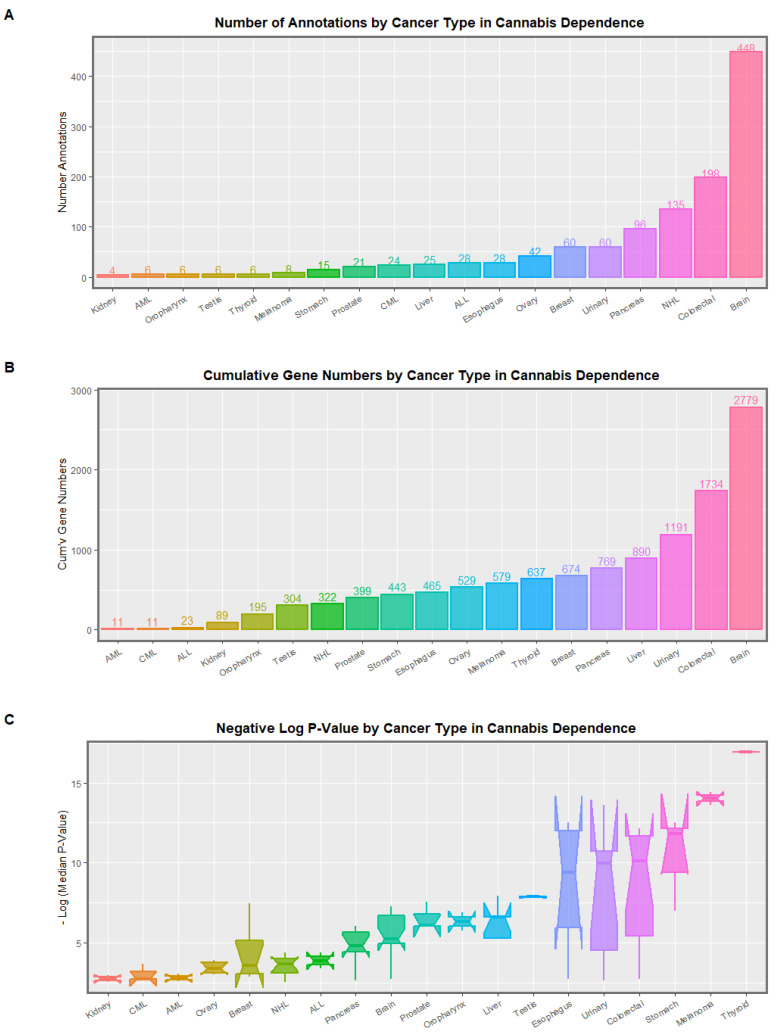
(**A**) Numbers of gene annotations, (**B**) numbers of genes affected and (**C**) negative logarithm of *p*-value by cancer type—cannabis dependence Schrott data.

Because the Schrott dataset is elegantly organized into both cannabis dependence and withdrawal it may be categorized for 19 tumors in cannabis dependence as shown in Supplementary Table S38, which is listed in descending order of minimum *p*-value. The cumulative gene number again includes duplicate mentions for some genes. This list is headed by thyroid, melanoma and urinary cancers. When the same list is ordered by median *p*-value the order of significance is thyroid, melanoma, stomach, colorectal urinary and testis cancer as indicated in Supplementary Table S39. Some of these key data are illustrated graphically in Figure 3 which lists the number of cancers, the cumulative gene number from the Schrott EWAS dataset, and the negative log of the *p*-value for each tumor type.

**Figure 4:**
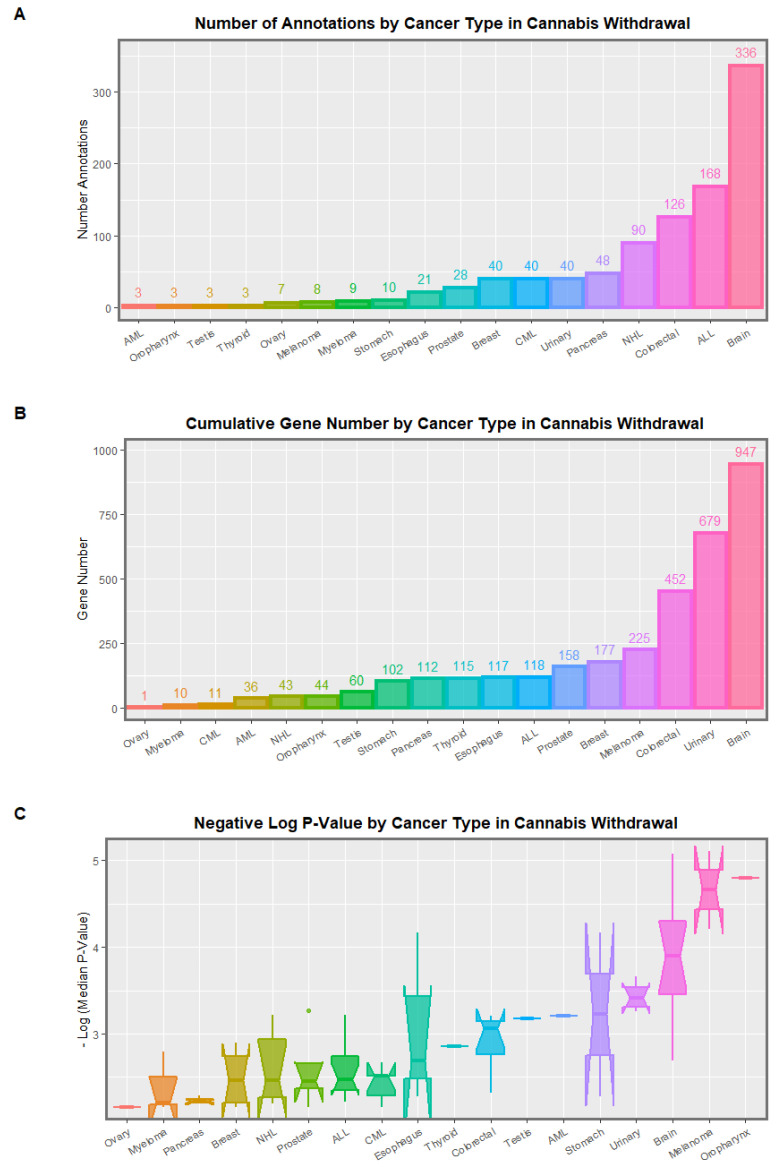
(**A**) Numbers of gene annotations, (**B**) numbers of genes affected and (**C**) negative logarithm of *p*-value by cancer type—cannabis withdrawal Schrott data.

Supplementary Table S40 lists the applicable *p*-values for cannabis withdrawal for 18 tumor types and is ordered by minimum *p*-value. The list is headed by melanoma, brain, oropharynx and esophageal cancers. These significance levels are noted to be lower than those in the preceding Tables. When the list is sorted by median *p*-value oropharynx, melanoma, brain, urinary, acute myeloid leukemia and testicular cancer head the list (Supplementary Table S41). These results are illustrated graphically in Figure 4 which shows, respectively, the number of gene annotations, the cumulative gene number and the negative log of the significance levels by tumor type.

Supplementary Figure S6 directly compares the significance levels of the tumors by cannabis dependency status. It is observed that the tumors are in a very different order and that the level of significance is generally much lower in cannabis withdrawal than in cannabis dependence.

Table 11 directly compares the significance levels and gene numbers for the various tumors types in dependence and withdrawal. Whilst the overall pattern is clearly that there are more genes implicated and at higher levels of statistical significance by cannabis dependence than cannabis withdrawal, there are a few notable exceptions to this pattern.

Both acute myeloid leukemia (AML) and acute lymphoid leukemia (ALL) have a lower gene number dependence/withdrawal ratio than unity. AML also has a lower minimum (and median and mean) *p*-value dependence/withdrawal ratio. Data are listed by the gene number ratio in Supplementary Table S42 and ovarian, Non-Hodgkins, pancreas thyroid and testicular cancers are noted to head up the list.

**Figure 5:**
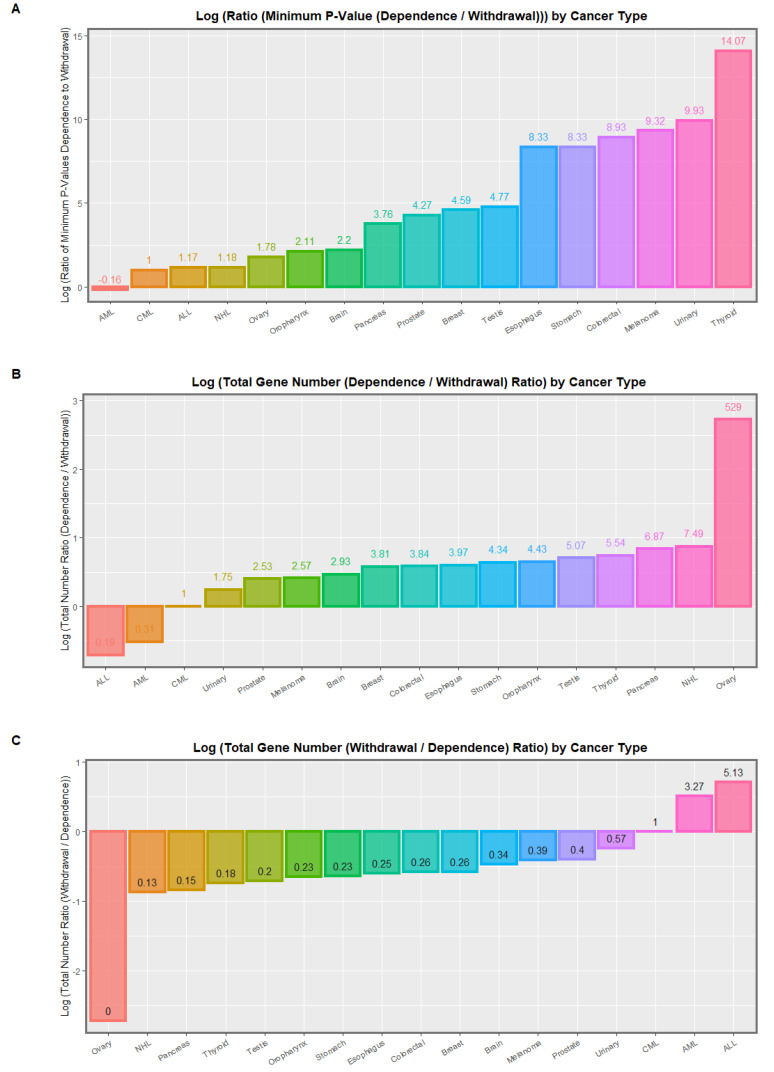
Log plots of significance levels for (**A**) ratio of *p*-values between cannabis dependence and withdrawal, (**B**) log of the dependence/withdrawal ratio of total gene numbers affected between cannabis dependence and withdrawal and (**C**) log of the withdrawal/dependence ratio of total gene numbers affected between cannabis dependence and withdrawal; each by tumor type from the Schrott EWAS data.

Some of these data are shown graphically in Figure 5 which lists the log of the ratio of the minimum *p*-values, the log of the gene number for dependence/withdrawal and the log the gene number for the withdrawal/dependence ratio. In this way, the distinctly higher withdrawal/dependence ratios in the pediatric AML and ALL cancers are highlighted.

### 3.3. Implications of Findings

From such a very broad array of objective reported results, basic cellular mechanisms and highly concordant epidemiological findings in both addiction medicine and aging science, it is necessary in discussing these results to highlight just a few key findings which are of particular importance to the overall flow of the main themes of this review and the major concepts presented. More detailed discussions have been presented in the references cited and other exhaustive and encyclopaedic sources [302,303,304,305,306,307]. The study is the first to combine and connect data from a broad range of genotoxic areas. Perhaps the most striking finding is the extraordinarily accurate predictive power of the epigenomic results to apparently explain the epidemiologically observed mutagenic and teratological phenomenology. This accuracy provides confirmation of the validity of the cannabis ageing mechanisms outlined in this paper. The epigenomic results of the Schrott group [27] not only predict with great accuracy such disparate findings as the high rates of atrial septal defect widely observed in Canada, Australia, Colorado, Hawaii, USA and Europe [103,107,108,111,114,115,116,118] and elevated rates of ventricular septal defect noted by the American Academy of Pediatrics and the American Heart Association and elsewhere [103,109,111,115,308], but also the mechanistically closely related pattern of congenital cardiac and renal anomalies which both share critical sensitivity to inhibition of the notch, sonic hedgehog and retinoic acid morphogenic pathways. Both atrial septal and ventricular septal defects feature prominently in the spectrum of cannabis teratological defects, and also in the multisyndromic VACTERL syndrome which formally relates renal, cardiac and limb anomalies (amongst others) and was the most strongly significantly cannabis-associated of all the European birth defects studied [114].

Findings also explain with extraordinary accuracy 20 cancers which are shared commonly between the epigenomic and epidemiological datasets featuring prominently liver, breast, pancreas, diverse leukemias and lymphomas, oropharyngeal, thyroid, urinary, esophageal and testicular tumors. These findings also accord closely with older published data which link cannabis to exposure of a range of tumors including lung, head and neck, larynx, brain, prostate, testis and urothelium [52,53,54,55,56,57,58,59,60,61,62].

The likely foundational importance of cannabis-induced epigenotoxicity implies that not only has the salience of epigenomic disturbances emerged as being pre-eminent from the mechanistic confusion surrounding the aging process itself [16] but in a similar way it appears that with time and further research the epigenomic perturbations induced by cannabis dependence and withdrawal are likely to be shown to be foundational in understanding the plethoric and protean manifestations of cannabinoid-induced mutagenesis, teratogenesis, carcinogenesis and indeed aging [2].

This foundational centrality of epigenotoxicity to the understanding of cannabinoid toxicity is highly reminiscent of the central understanding which the fundamentally epigenomic nature of fetal alcohol syndrome has been shown to display [309,310,311,312,313,314,315,316,317,318,319,320]. Indeed, fetal alcohol syndrome has been shown to be primarily mediated epigenomically via cannabinoid type 1 receptors (CB1Rs) [321,322,323,324,325,326,327,328,329,330,331]. It should come therefore as little surprise to note that cannabinoids can also act via CB1Rs with a unique spectrum of clinical manifestations.

One major corollary of the finding of the primacy of epigenomic toxicity is that at least some of these changes are likely to be epigenetically inheritable. Indeed, a heritable pediatric fetal cannabinoid syndrome, analogous to fetal alcohol syndrome has been previously proposed [321,322,323,327,328,329,330,332,333,334]. In the case of the pediatric cancers acute myeloid and lymphoid leukemia [65,66,206], this implies not only heritable teratogenicity but also heritable teratogenic carcinogenicity [204,205]. This finding likely also applies to other pediatric tumors previously linked with parental cannabis exposure such as rhabdomyosarcoma, neuroblastoma and astrocytoma [207,208,321,322,323,327,328,329,330,332,333,334].

It was noted that the ratios of the most significant *p*-values were inverted for the pediatric tumor ALL, and for AML of which some cases occur early in life. This suggests the intriguing possibility that it is the cannabis withdrawal state following birth which triggers and launches the leukemogenic gene cassettes of childhood.

Many other features of the above series of results stand out prominently. The high numbers and wide ranges of both congenital anomalies-45/62 in USA and 89/95 in Europe (Table 6)-and cancers-33/40 in Europe and 25/28 in USA (Table 10)-are striking both in their own right and by virtue of the range of tissues and organ systems affected. As these observations have been made previously [66,103,112,113,114,115,116,120,121,122], they do not form the primary focus of the present investigation. What is more important for the present discussion of cannabis-related aging and its mechanisms is the relationship of oncogenicity and teratogenesis to aging related processes.

The North Carolina group reported that the significance of the DMR’s in cannabis dependence was higher than cannabis withdrawal [27]. Hence most of the ratios for the gene numbers affected in Table 11 were expected. However, the higher gene numbers affected in ALL (primarily a pediatric cancer) and AML (occasionally a pediatric cancer) and the higher significance level in AML found in withdrawal were unexpected and raise the intriguing possibility that the cannabis withdrawal state following birth may trigger leukemogenic gene activation. Whether this holds true for the other pediatric cancers previously related to cannabis remains to be studied. Moreover, this topic was shown to be of much greater significance beyond the field of pediatric cancer by the recent finding that many adult haemopoietic tumors have been shown to commence in fetal life [335], a finding which these latter investigators note may also apply more widely to the field of solid organ tumorigenesis.

One of the prominent findings to emerge from the above epidemiological overview was the salience of chromosomal disorders in both the congenital anomaly and the cancer datasets. Trisomies or monosomies of chromosomes 13, 18, 21 and X (including syndromes described by Turner and Klinefelter) were observed directly [66,103,115,120]. Moreover, strong signals were detected for acute lymphoid leukemia (which has been shown to often involve translocations between chromosomes 4, 9, 10, 11 and 22) [105,336] and testicular cancer [105,112,113,114,121,122] (which has been shown to implicate chromosomes 1, 7, 8, 11, 12, 13, 18, 21, X and Y) [337]. The total length of these chromosomes together comprehends 1754 megabases of the 3000 megabases, or 59%, of the whole human genome directly impacted by cannabinoid-related genotoxicity/epigenotoxicity. Deletions of chromosome 22 in USA and microdeletions in Europe were also significantly cannabis-associated [66,103,115]. These data make the issue of chromosomal non-segregation, non-disjunction, aneuploidy, chromosomal breaks and translocations and subsequent teratogenic malignancy a very prominent feature of cannabis related genotoxicity. As described in considerable detail in the pathophysiological review, multiple direct and epigenomic pathways exist which comfortably explain and may account for these prominent and important clinical findings of hundred megabase scale epi/genotoxic activities.

Given so much powerful evidence for cannabinoid-related epigenotoxicity, the possibility that these epigenomic changes are potentially reflected as pro-ageing effects effectively accelerating natural aging warrants particularly careful consideration. On this issue, three tissues are of particular and pivotal importance namely: spermatocytes, oocytes and zygotes.

### 3.4. Spermatocytes

Classic photomicrographs of cannabis exposed sperm featuring multiple (up to four) heads, multiple tails, obviously deformed heads and tails on a background of proteinaceous and inflamed tissue [24] along with gross chromosomal translocations and ring and chain formation [23,260] give an obviously degenerate genotoxic appearance. Multiple cannabinoids are known to induce adverse mitochondrial effects, reduced energy charge and increased free radial flux [33,140] which are all changes that are well established as being age related. It has been shown that cannabinoid signaling via CB1R has a deleterious effect on sperm chromatin which increases along the epididymis, altered histone-protamine substitution via inhibition of transition protein 2 (TNP2) and leads to genome DNA fragmentation with compromise of male fertility [34]. Moreover, the above demonstration of cannabinoid-related gross changes to the tubulin code and meiotic apparatus (Supplementary Tables S9–S13) implies that not only are the microtubules of the sperm flagellum disrupted but so also are those comprising the sperm centrioles and first and second meiotic spindles. Since all of these various changes are age-defining and age-causing disorders, this implies that the age of cannabinoid-exposed sperm is advanced.

### 3.5. Oocytes

Diminished ovarian reserve was noted in the epigenomic dataset of Schrott (Supplementary Table S27; Schrott [27] Page 349) and is both a defining feature of female aging [1] and an important cause thereof [200]. Gross and severe morphological changes were noted in cannabis exposed oocytes induced to divide including chromosomal nucleoplasmic bridges, non-disjunctions, tripolar, quadripolar and pentapolar cell divisions along with an extremely high (20%) rate of oocyte death with just a single cell division. Moreover, oocyte depletion has been attributed primarily to failure of DNA damage repair [230]. As noted above, cannabis has been shown to suppress pituitary FSH secretion thereby interfering with the normal female hormonal cycle. All of these are clearly age-related and age-inducing changes.

### 3.6. Zygotes

Since both sperm and oocytes bear many chromosomal, genetic and epigenetic features of aging, it seems clear that these changes would persist in the pronuclei of the fertilized zygote and carry important influences into the first few rounds of zygotic cell division which are epigenetically controlled from the time of fertilization. These deleterious changes would be compounded by aberrant histone and protamine changes in sperm and by the disrupted tubulin code known to be borne by sperm. Together, these changes indicate that not only are the gametes themselves aged, but so too must the fertilized zygote be aged from—and actually even prior to—conception. It is noted again that the fragile process of human female meiosis is highly error prone ordinarily [248] which suggests that the tolerance for error under the influence of external xenobiotic genotoxic agents is very narrow indeed. These considerations raise the intriguing and very concerning possibility that the zygote itself may manifest advanced epigenomic age from even before fertilization and conception. It is noted that the newly described method of analysis of blastocystoid bodies derived from induced human pluripotential embryonic stem cells (iPS) might provide an ideal and ethical laboratory method to formally assess these issues [338].

It was recently shown that a key part in sperm maturation is played by the addition of mRNA exosomes (as epididymosomes) in the tail of the epididymis during sperm maturation. These extracellular packages of mRNA play a key part in early embryonic development during the initial divisions of the fertilized zygote and are under close control at several points by CB1R-mediated cannabinoid control [339]. Interference with this normal mechanism led to profound perturbation of sperm maturation, fertility and function. In this regard, the human system closely mirrors that seen in mice.

### 3.7. Cannabidiol and Δ8THC

At the time of writing, cannabidiol and Δ8THC have been allowed to freely penetrate culture without restriction in many places and have been made available in cookies, sauces, lollies, candies, crackers and in solid translucent blocks often being marketed as “legal weed”.

In such a context, it is important to note that it was found long ago that the genotoxic moiety of cannabinoids lies primarily in their central olevitol nucleus, an activity which is little modified by their various side chains [340,341]. This important finding implicates most cannabinoids in genotoxic effects.

Cannabidiol has an experimental [24,342,343,344,345] and an epidemiological literature describing its genotoxic effects in both cancer [112,113,114] and congenital anomalies [103]. Cannabidiol is also genotoxic by virtue of its involvement in signaling via the nuclear receptor—transcription factor PPARγ (Peroxisome proliferator receptor gamma) [346,347,348,349,350,351,352,353], by its inhibition of mitochondrial respiration which forms the energetic and co-factor substrate basis for the epigenomic machinery [41,42,354,355,356,357,358,359,360,361], and by its interaction at higher doses [362,363,364,365,366,367,368,369,370] with the cannabinoid type 1 receptors present on mitochondria themselves [44,145,146,147,371,372,373,374]. Importantly, the PPARγ nuclear signal is transduced by binding to retinoic acid receptors (RXR) which together then bind the genome [375]. Similarly, Δ8THC has been epidemiologically implicated in both cancer [376] and birth defects [377].

A recent very concerning paper demonstrated not only that many cannabinoids (including Δ9THC, Δ8THC and cannabidiol) could freely pass into the milk of dairy cattle fed legal hemp (with nominally less that 0.3% THC content) but that the cannabinoid concentration in milk could rise to a level where the total recommended daily dose of Δ9THC was exceeded [119]. Moreover, the cows themselves became obviously ataxic and “stoned” and stood motionless for extended period, not moving and not eating, apparently “stoned”. They were also ataxic and had difficulty walking. After cessation of the hemp/cannabinoid feed, these changes abruptly declined. Most concerningly, the levels of cannabinoid found in the feed when analyzed by state-of-the-art tandem liquid chromatography/gas chromatography—mass spectrometry (LCGC-MS) techniques were more than ten times those found with the standard legally prescribed tests for cannabinoid, a finding which necessarily impugns and indicts so called legally safe “low-THC” hemp products and imperils public heath and safety.

Moreover, such findings dramatically and eloquently illustrate the florid manner in which such grossly affected animals in the food chain might pass on the severe genotoxic cannabinoid-mediated damage (which includes limblessness) as has been chronicled in recent reports from France and Germany [378,379,380,381].

## 4. Conclusions

Many metrics, including hormonal, mitochondriopathic, cardiovascular, hepatotoxic, immunological, genotoxic, epigenotoxic, disruption of chromosomal physiology, congenital anomalies, cancers including inheritable tumorigenesis, telomerase inhibition and elevated mortality point towards cannabinoid-exposed tissues being of advanced biological age. Evidence from many studies indicates extensive perturbation of the human epigenome by exposure to many cannabinoids. Since the epigenome has emerged as the key and central mediator of the panorganismal aging process [13,14,15,16,202,382], it becomes of primary importance to investigate its likely implication in aging processes directly by the application of late-generation epigenomic clocks [383,384,385,386,387,388,389]. The likely involvement of spermatogonia, oocyte and fertilized zygote in this accelerated aging process increases the importance of this enquiry for the health of subsequent generations, an enquiry which is heightened and intensified by the transgenerational transmission of cannabinoid-related epigenotoxicity in human sperm [26,27], to subsequent rodent generations [28,29,30,31,32,390], for pediatric brain function and development including autistic-like disorders [117,174,288,290,292,295,296,297,298,299,391] and through the heritable passage of many birth defects [103,108,109,110,111,115,118,120] including several pediatric cancers [63,64,65,66,105,121]. Inversion of the ratio of the minimum *p*-values between dependence and withdrawal for ALL and AML may imply that it is the activation of leukemogenic gene cassettes by the withdrawal state occasioned by birth which gives rise to these pediatric cancers. The genotoxic, epigenotoxic, mutagenic and teratological issues raised are clearly very serious and have been shown several times to greatly outweigh those attributable to tobacco and alcohol [103,112,113,114,115]. These changes carry such far-reaching public health implications that they are worthy of investigation by the most advanced multiomics techniques including multichannel single cell epigenomic and 3D chromosomal topological techniques with appropriate resourcing to exhaustively perform these investigations in a translational multigenerational context.

## Figures and Tables

**Table 1:** Outline of Paper.

No.	Streams of Evidence	Focus of the Discussion
Section 3.1.1	Clinical syndromes	Clinical phenomenology
Section 3.1.2	Mitochondrial inhibition	Cellular systems and mechanisms
Section 3.1.3	DNA Methylation	Cellular systems and mechanisms
Section 3.1.4	Mental illnesses	Organ systems
Section 3.1.5	Cardiovascular age	Organ systems
Section 3.1.6	Endocrine suppression	Organ systems
Section 3.1.7	Liver inflammation	Organ systems
Section 3.1.8	Cancer	Heath disorders and Population impacts
Section 3.1.9	Inheritable cancer	Heath disorders and Population impacts
Section 3.1.10	Congenital Anomalies	Heath disorders and Population impacts
Section 3.1.11	Telomerase inhibition	Cellular systems and mechanisms
Section 3.1.12	Elevated Mortality rate	Epidemiological Studies
	**Pathogenetic Field of Interest**	
Section 3.2.1	Epigenomic Overview	Cellular systems and mechanisms
Section 3.2.2	Stem-Cell Factors	Cellular systems and mechanisms
Section 3.2.3	Chromosomal Mechanics	Cellular systems and mechanisms
Section 3.2.4	Centromeres and Kinetochores	Cellular systems and mechanisms
Section 3.2.5	Prefrontal cortex and Brain	Organ systems
Section 3.2.6	Cardiovascular System	Organ systems
Section 3.2.7	Teratogenesis	Analysis DNA Methylation data and epidemiological impacts
Section 3.2.8	Carcinogenesis	Analysis DNA Methylation data and epidemiological impacts

**Table 2:** Overview of Cannabis Impacts on Epigenetic Machinery, Schrott EWAS Data.

Nearest Gene Name	Chromosome Number	Nearest Gene Number	Dependency Status	Functional Annotation	Page	Distance from Nearest Gene	Relative Position	*p*-Value	Bonferroni Adjusted *p*-Value
**DNA Methyltransferases**									
DNMT1	19	ENSG00000130816	Withdrawal	Maintenance DNA methyltransferase	156	0	Intron	1.89 × 10^−6^	0.010563
DNMT1	19	ENSG00000130816	Withdrawal	Maintenance DNA methyltransferase	179	0	Intron	4.81 × 10^−6^	0.016176
DNMT3B	20	ENSG00000088305	Dependence	de novo DNA methyltransferase	109	0	Intron	1.22 × 10^−5^	0.023205
DNMT3B	20	ENSG00000088305	Withdrawal	de novo DNA methyltransferase	125	1067	Upstream	2.08 × 10^−8^	0.001062
DNMT3A	2	ENSG00000119772	Withdrawal	de novo DNA methyltransferase	194	0	Intron	7.57 × 10^−6^	0.020149
**DNA Demethylases**									
TET1	10	ENSG00000138336	Dependence	Ten-Eleven translocase	107	0	Intron	1.18 × 10^−5^	0.022782
TET1P1	13	ENSG00000232204	Dependence	Pseudogene for TET	63	36,150	Downstream	4.14 × 10^−6^	0.013905
TET1P1	13	ENSG00000232204	Dependence	Pseudogene for TET	85	47,940	Upstream	7.47 × 10^−6^	0.018443
TET1P1	13	ENSG00000232204	Dependence	Pseudogene for TET	98	9930	Downstream	9.97 × 10^−6^	0.021086
TET1P1	13	ENSG00000232204	Dependence	Pseudogene for TET	98	55,192	Upstream	6.32 × 10^−6^	0.018533
**Others**									
UHRF1	19	ENSG00000276043	Withdrawal	Integrator of epigenetic information	128	0	Intron	5.74 × 10^−8^	0.001782
UHRF1BP1L	12	ENSG00000111647	Withdrawal	Regulator of UHRF1	155	0	Intron	1.79 × 10^−6^	0.010239
UHRF1BP1L	12	ENSG00000111647	Withdrawal	Regulator of UHRF1	233	0	Intron	1.67 × 10^−5^	0.028881
DPPA2	3	ENSG00000163530	Dependence	Developmental Pluripotency Associated 2	40	15,599	Downstream	1.66 × 10^−6^	0.009001
DPPA2	3	ENSG00000163530	Dependence	Developmental Pluripotency Associated 2	133	6894	Downstream	1.90 × 10^−7^	0.003298
DPPA2P1	Y	ENSG00000223915	Withdrawal	Pseudogene for DPPA2A	135	26,055	Upstream	2.78 × 10^−7^	0.004034
**Telomerase**									
TERT	5	ENSG00000223915	Dependence	Telomerase	44	4227	Upstream	2.82 × 10^−6^	0.012582
**Polycomb Repressors**									
PCGF6 in PRC1	10	ENSG00000156374	Dependence	Polycomb Repressive Complex 1	65	0	Intron	4.37 × 10^−6^	0.014300
PCGF6 in PRC1	10	ENSG00000156374	Withdrawal	Polycomb Repressive Complex 1	137	0	Intron	4.03 × 10^−7^	0.004978
EZH2 in PRC2	7	ENSG00000180628	Dependence	Polycomb Repressive Complex 2	94	0	Intron	9.22 × 10^−6^	0.020342
**Chromatin Remodellers**									
SMARCA2	9	ENSG00000080503	Dependence	SWI/SNF Matrix, Actin Chromatin Regulator 2	6	0	Intron	5.27 × 10^−9^	0.000438
SMARCA2	9	ENSG00000080503	Dependence	SWI/SNF Matrix, Actin Chromatin Regulator 2	62	3071	Downstream	4.00 × 10^−6^	0.013641
SMARCA2	9	ENSG00000080503	Dependence	SWI/SNF Matrix, Actin Chromatin Regulator 2	114	0	Intron	1.34 × 10^−5^	0.024371
SMARCA4	19	ENSG00000127616	Withdrawal	SWI/SNF Matrix, Actin Chromatin Regulator 4	145	9567	Upstream	8.86 × 10^−7^	0.007300
SMARCA4	19	ENSG00000127616	Withdrawal	SWI/SNF Matrix, Actin Chromatin Regulator 4	199	9258	Upstream	8.54 × 10^−6^	0.021311

**Table 3:** Cannabis Impacts on Yamanaka Stem-Cell Factors, Schrott EWAS Data.

Nearest Gene Name	Chromosome Number	Nearest Gene Number	Dependency Status	Functional Annotation	Page	Distance from Nearest Gene	Relative Position	*p*-Value	Bonferroni Adjusted *p*-Value
POU5F1P2	8	ENSG00000253382	Dependence	Oct3/4 Pseudogene	5	2871	Downstream	1.49 × 10^−9^	0.000216
SOX2-OT	3	ENSG00000242808	Dependence	Sox2 Overlapping Transcript	6	0	Intron	5.25 × 10^−9^	0.000438
SOX2-OT	3	ENSG00000242808	Dependence	Sox2 Overlapping Transcript	48	0	Intron	2.38 × 10^−^6	0.017245
SOX2-OT	3	ENSG00000242808	Dependence	Sox2 Overlapping Transcript	88	0	Intron	8.12 × 10^−6^	0.019185
SOX2-OT	3	ENSG00000242808	Withdrawal	Sox2 Overlapping Transcript	116	0	Intron	1.40 × 10^−5^	0.024849
SOX2-OT	3	ENSG00000242808	Withdrawal	Sox2 Overlapping Transcript	146	0	Intron	9.74 × 10^−7^	0.007679
SOX2-OT	3	ENSG00000242808	Withdrawal	Sox2 Overlapping Transcript	211	0	Intron	1.11 × 10^−5^	0.023974
Klf4	9	ENSG00000136826	Dependence	Kruppel-like factor 4	117	12,186	Upstream	1.41 × 10^−5^	0.024968
MycBP2	13	ENSG00000005810	Dependence	Myc Binding Protein 2	49	0	Intron	2.50 × 10^−6^	0.010960
MycBP2	13	ENSG00000005810	Withdrawal	Myc Binding Protein 2	153	0	Intron	1.58 × 10^−6^	0.009647
Myc	8	ENSG00000136826	Withdrawal	Myc proto-oncogene	227	23,489	Downstream	1.49 × 10^−5^	0.027466

**Table 4:** Cannabis Impacts on Centrosomal Proteins, Schrott EWAS Data.

Nearest Gene Name	Nearest Gene Number	Chromosome Number	Relative Location	Distance to Nearest Gene (Bases)	Number of Annotations	*p*-Value	Bonferroni-Adjusted *p*-Value
**Centrosomal Proteins**							
CENPIP1	ENSG00000224778	13	Upstream	1100	1	2.38 × 10^−9^	0.000279
CENPF	ENSG00000117724	1	Downstream	72,569	3	2.98 × 10^−8^	0.001109
CNEPVL3	ENSG00000224109	X	Downstream	2146	1	2.80 × 10^−6^	0.001153
CENPK	ENSG00000123219	5	Intron	0	1	8.01 × 10^−6^	0.019098
CNEPP	ENSG00000188312	9	Intron	0	2	8.26 × 10^−6^	0.019330
CNEPJ	ENSG00000151849	13	Exon	0	1	4.66 × 10^−7^	0.005279
CNEPUP1	ENSG00000255075	11	Upstream	8401	1	2.81 × 10^−6^	0.012567
INCENP	ENSG00000149503	11	Intron	0	1	3.07 × 10^−6^	0.013077
CNEPO	ENSG00000138092	2	Exon	0	1	6.25 × 10^−6^	0.018393
CNEPI	ENSG00000102384	X	Intron	0	2	7.54 × 10^−6^	0.020123
CNEPL	ENSG00000120334	1	Intron	0	1	8.22 × 10^−6^	0.020943
CNEPX	ENSG00000169689	17	Exon	0	1	9.35 × 10^−6^	0.022176
CNEPC	ENSG00000145241	4	Intron	0	1	9.60 × 10^−6^	0.002248
CENPV	ENSG00000166582	17	Upstream	13,237	2	1.63 × 10^−5^	0.002861
CENPN	ENSG00000166451	16			86	7.73 × 10^−20^	
**Others**							
KNL1	ENSG00000137812	15	3UTR	0	1	7.71 × 10^−7^	0.006173
ZWINT	ENSG00000122952	10	Downstream	58,081	1	6.00 × 10^−6^	0.016644
NUF2	ENSG00000143228	1	Intron	0	1	1.12 × 10^−6^	0.007421
SPC24	ENSG00000161888	19	3UTR	0	1	1.61 × 10^−6^	0.009713
**Sumoylation**							
SUMO1	ENSG00000112701	2	Intron	0	1	1.25 × 10^−5^	0.023445
ZNF451	ENSG00000226803	6	Intron	0	1	2.22 × 10^−6^	0.011398
SENP6	ENSG00000112701	6	Intron	0	1	3.12 × 10^−6^	0.013217
SENP7	ENSG00000138468	3	Intron	0	1	4.73 × 10^−6^	0.014903
SENP7	ENSG00000138468	3	Intron	0	1	1.16 × 10^−5^	0.024458

**Table 6:** Comparative Lists of Significantly Cannabinoid-Associated Congenital Anomalies in Europe and USA.

No.	Europe				USA			
	Congenital Anomaly	Term	Model	*p*-Value	Congenital Anomaly	Term	Model	*p*-Value
1	Abdominal Wall Defects	pm.Resin.Daily	Categorical	3.01 × 10^−120^				
2	All Anomalies	Daily_Use	Categorical	<2.2 × 10^−320^				
3	Amniotic band	pm.Resin.Daily	Categorical	1.09 × 10^−47^				
4	Anencephalus and similar	Resin_THC	Categorical	1.53 × 10^−212^				
5	Annular Pancreas	Daily_Use	Categorical	1.52 × 10^−13^				
6	Anophthalmos	Daily_Use	Categorical	1.06 × 10^−6^				
7	Ano-rectal atresia and stenosis	pm.Resin.Daily	Categorical	4.03 × 10^−39^	Large intestinal and Rectal atresia/stenosis	Cannabidiol_Estimates	Continuous	0.0040
8	Anotia	Herb_THC	Categorical	4.63 × 10^−13^	Anotia/microtia	LM_Cannabis	Continuous	7.57 × 10^−4^
9	Aortic atresia/interrupted aortic arch	LM.Cann_Resin_THC	Categorical	5.71 × 10^−25^	Interrupted aortic arch	LM_Cannabis	Continuous	3.40 × 10^−6^
10	Aortic Valve stenosis/atresia	Herb_THC	Categorical	7.14 × 10^−13^	Aortic valve stenosis	LM_Cannabis	Continuous	0.0019
11	Arhinencephaly/holoprosencephaly	LM_Herb.Daily	Continuous	0.0052				
12	Arterial Truncus	pm.Herb.Daily	Categorical	9.92 × 10^−7^				
13	Atrial septal defect (ASD)	Herb_THC	Categorical	<2.2 × 10^−320^	Atrial septal defect (ASD)	LM_Cannabis	Continuous	0.0378
14	Atrioventricular septal defect (AVSD)	pm.Resin.Daily	Categorical	1.65 × 10^−101^	Atrioventricular septal defect (AVSD)	LM_Cannabis	Categorical	0.0470
15	Bilateral renal agenesis including Potter syndrome	Herb_THC	Categorical	1.08 × 10^−47^	Renal agenesis/hypoplasia	LM_Cannabis	Continuous	7.34 × 10^−4^
16	Bile duct atresia	Daily_Use	Categorical	1.00 × 10^−40^	Biliary atresia	Cannabidiol_Estimates	Continuous	2.43 × 10^−4^
17	Bladder Extrophy/Epispadias	pm.Resin.Daily	Categorical	1.56 × 10^−18^	Bladder extrophy	LM_Cannabis	Continuous	0.0170
18	Choanal Atresia	Herb_THC	Categorical	7.34 × 10^−94^	Choanal atresia	Δ9THC_Estimates	Continuous	0.0033
19	Chromosomal	Daily_Use	Categorical	<2.2 × 10^−320^	Chromosomal	LM_Cannabis	Mixed Effects	9.38 × 10^−30^
20	Cleft lip with or without palate	Herb_THC	Categorical	1.80 × 10^−101^	Cleft lip with and without cleft palate	Cannabidiol_Estimates	Categorical	0.0159
21	Cleft palate	Herb_THC	Categorical	1.79 × 10^−34^	Cleft palate alone	LM_Cannabis	Continuous	0.0014
22					Cloacal exstrophy	LM_Cannabis	Categorical	2.13 × 10^−86^
23	Club foot-talipes equinovarus	Daily_Use	Categorical	4.23 × 10^−292^	Clubfoot	LM_Cannabis	Continuous	3.16 × 10^−5^
24	Coarctation Aorta	Daily_Use	Categorical	5.78 × 10^−33^	Coarctation of the aorta	LM_Cannabis	Categorical	9.74 × 10^−45^
25	Congenital cataract	Daily_Use	Categorical	4.88 × 10^−66^	Congenital cataract	LM_Cannabis	Continuous	0.0479
26	Congenital glaucoma	Daily_Use	Categorical	1.52 × 10^−43^				
27	Congenital Heart	pm.Herb.Daily	Categorical	<2.2 × 10^−320^				
28	Conjoined twins	Daily_Use	Categorical	8.62 × 10^−14^				
29	Craniosynostosis	Daily_Use	Categorical	5.72 × 10^−155^				
30	Cystic adenomatous malformation of lung	Daily_Use	Categorical	4.05 × 10^−80^				
31	Diaphragmatic Hernia	Daily_Use	Categorical	8.77 × 10^−57^	Diaphragmatic hernia	LM_Cannabis	Categorical	2.11 × 10^−8^
32	Digestive system	pm.Herb.Daily	Categorical	1.61 × 10^−264^				
33	Double outlet right ventricle	pm.Herb.Daily	Categorical	1.28 × 10^−46^	Double outlet right ventricle	LM_Cannabis	Categorical	7.31 × 10^−4^
34	Down Syndrome	Daily_Use	Categorical	<2.2 × 10^−320^	Trisomy 21 (Down syndrome)	LM_Cannabis	Categorical	4.02 × 10^−26^
35	Duodenal stenosis/atresia	Herb_THC	Categorical	1.50 × 10^−10^				
36	Ear, face and neck	Daily_Use	Categorical	3.38 × 10^−44^				
37	Ebstein’s Anomaly	pm.Resin.Daily	Categorical	3.23 × 10^−17^				
38	Edward syndrome/Trisomy 18	Daily_Use	Categorical	<2.2 × 10^−320^	Edward syndrome/Trisomy 18	LM_Cannabis	Categorical	1.06 × 10^−61^
39	Encephalocele	pm.Resin.Daily	Categorical	4.76 × 10^−21^	Encephalocele	LM_Cannabis	Continuous	0.0013
40					Epispadias	LM_Cannabis	Continuous	0.0111
41	Eye	Daily_Use	Categorical	2.27 × 10^−175^				
42	Fetal alcohol syndrome	pm.Resin.Daily	Categorical	5.88 × 10^−57^				
43	Gastroschisis	Herb_THC	Categorical	6.55 × 10^−39^				
44	Genetic syndromes + microdeletions	pm.Herb.Daily	Categorical	1.38 × 10^−228^	Deletion 22q11.2	LM_Cannabis	Continuous	0.0024
45	Genital	pm.Herb.Daily	Categorical	2.55 × 10^−243^				
46	Hip dislocation and/or dysplasia	Daily_Use	Categorical	<2.2 × 10^−320^	Congenital hip dislocation	LM_Cannabis	Categorical	7.27 × 10^−70^
47	Hirschsprung’s disease	Daily_Use	Categorical	2.54 × 10^−88^	Hirschsprung disease (congenital megacolon)	LM_Cannabis	Categorical	6.69 × 10^−6^
48	Holoprosencephaly/Arhinencephaly	LM_Cannabis	Categorical	1.22 × 10^−72^	Holoprosencephaly	LM_Cannabis	Categorical	2.90 × 10^−12^
49	Hydrocephalus	pm.Herb.Daily	Categorical	1.76 × 10^−110^				
50	Hydronephrosis	Herb_THC	Categorical	<2.2 × 10^−320^				
51	Hypoplastic Left Heart	Daily_Use	Categorical	3.37 × 10^−61^	Hypoplastic left heart syndrome	LM_Cannabis	Continuous	0.0047
52	Hypoplastic right heart	Resin_THC	Categorical	2.85 × 10^−59^				
53	Hypospadias	pm.Herb.Daily	Categorical	2.92 × 10^−177^	Hypospadias	LM_Cannabis	Continuous	1.16 × 10^−5^
54	Klinefelter syndrome	Daily_Use	Categorical	1.75 × 10^−41^				
55					Large intestinal and Rectal atresia/stenosis	Cannabidiol_Estimates	Continuous	0.0040
56	Lateral anomalies	LM.Cann_Herb_THC	Categorical	2.36 × 10^−48^				
57	Limb anomalies	pm.Herb.Daily	Categorical	<2.2 × 10^−320^				
58	Limb reductions	Daily_Use	Categorical	8.20 × 10^−65^	Limb deficiencies (reduction defects)	LM_Cannabis	Continuous	0.0134
59					Lower limb Reduction deformity	LM_Cannabis	Continuous	0.0420
60	Maternal infections resulting in malformations	Daily_Use	Categorical	4.15 × 10^−87^				
61	Microphthalmos/Anophthalmos	Daily_Use	Categorical	1.25 × 10^−55^	Microphthalmos/Anophthalmos	Δ9THC_Estimates	Continuous	0.0045
62	Mitral valve anomalies	pm.Herb.Daily	Categorical	8.99 × 10^−58^				
63	Multicystic renal dysplasia	pm.Resin.Daily	Categorical	6.70 × 10^−251^				
64	Nervous system	pm.Herb.Daily	Categorical	<2.2 × 10^−320^				
65	Neural Tube Defects	Resin_THC	Categorical	9.97 × 10^−269^				
66					Obstructive genitourinary defect	Cannabidiol_Estimates	Categorical	2.22 × 10^−15^
67	Oesophageal stenosis/atresia	Daily_Use	Categorical	3.49 × 10^−44^	Oesophageal atresia/tracheoesophageal fistula	LM_Cannabis	Continuous	4.83 × 10^−6^
68	Omphalocele	pm.Resin.Daily	Categorical	4.94 × 10^−131^	Omphalocele	LM_Cannabis	Continuous	0.0025
69	Oro-facial clefts	Herb_THC	Categorical	3.99 × 10^−133^				
70	Patau syndrome/trisomy 13	Daily_Use	Categorical	1.08 × 10^−144^	Patau syndrome/trisomy 13	LM_Cannabis	Continuous	2.08 × 10^−7^
71	PDA as only CHD in term infants (>=37 weeks)	pm.Herb.Daily	Categorical	2.14 × 10^−20^				
72	Polydactyly	pm.Resin.Daily	Categorical	1.46 × 10^−292^				
73	Posterior urethral valve and/or prune belly	pm.Resin.Daily	Categorical	1.28 × 10^−42^	Congenital posterior urethral valves	LM_Cannabis	Continuous	1.18 × 10^−4^
74	Pulmonary valve atresia	Daily_Use	Categorical	1.42 × 10^−27^	Pulmonary valve atresia	Cannabidiol_Estimates	Categorical	1.02 × 10^−5^
75	Pulmonary valve stenosis	Daily_Use	Categorical	2.09 × 10^−95^				
76	Respiratory	pm.Herb.Daily	Categorical	2.57 × 10^−203^				
77	Severe CHD	Herb_THC	Categorical	1.81 × 10-317				
78	Severe microcephaly	pm.Herb.Daily	Categorical	3.17 × 10^−148^				
79	Single ventricle	Daily_Use	Categorical	1.03 × 10^−25^	Single ventricle	LM_Cannabis	Categorical	0.0060
80	Situs inversus	Daily_Use	Categorical	1.42 × 10^−44^				
81	Skeletal dysplasias	Daily_Use	Categorical	5.12 × 10^−74^				
82	Small Intestine stenosis/atresia	pm.Herb.Daily	Categorical	8.23 × 10^−31^	Small intestinal atresia/stenosis	Cannabidiol_Estimates	Continuous	3.39 × 10^−6^
83	Spina Bifida	Resin_THC	Categorical	3.93 × 10^−84^	Spina bifida without anencephalus	Δ9THC_Estimates	Continuous	0.0008
84	Syndactyly	pm.Resin.Daily	Categorical	3.47 × 10^−16^				
85	Teratogenic syndromes with malformations	Daily_Use	Categorical	1.42 × 10^−139^				
86	Tetralogy of Fallot	Daily_Use	Categorical	3.12 × 10^−47^	Tetralogy of Fallot	LM_Cannabis	Continuous	0.0168
87	Total Anomalous Pulmonary Venous Return	Herb_THC	Categorical	4.07 × 10^−09^	Total anomalous pulmonary venous connection	LM_Cannabis	Continuous	0.0299
88	Transposition of great vessels	Resin_THC	Categorical	9.96 × 10^−33^	Transposition of great arteries	Cannabidiol_Estimates	Continuous	0.0479
89	Turner syndrome	Daily_Use	Categorical	1.10 × 10^−146^	Turner syndrome	LM_Cannabis	Categorical	7.69 × 10^−49^
90	Tricuspid valve stenosis/atresia	Daily_Use	Categorical	6.86 × 10^−24^				
91	Urinary	pm.Resin.Daily	Categorical	<2.2 × 10^−320^				
92	Valproate syndrome	Daily_Use	Categorical	1.57 × 10^−7^				
93	Vascular disruption anomalies	Herb_THC	Categorical	3.46 × 10^−101^				
94	VATER/VACTERL	pm.Herb.Daily	Categorical	2.43 × 10^−36^				
95	Ventricular septal defect (VSD)	pm.Resin.Daily	Categorical	<2.2 × 10^−320^	Ventricular septal defect	LM_Cannabis	Continuous	0.0021

Abbreviations: pm—Past month cannabis use. LM.Cann—Last Month Cannabis Use. Herb_THC—THC concentration of cannabis herb. Resin_THC—THC concentration of cannabis herb. DailyUse—Percent using daily or almost daily. LM_Herb.Daily = LM.Cann × DailyUse. LM.Cann_Herb_THC = LM.Cann × Herb_THC. LM.Cann_Resin_THC = LM.Cann × Resin_THC. pm.Herb.Daily = pm × Herb_THC × Daily_Use. pm.Resin.Daily = pm × Resin_THC × Daily_Use.

**Table 7:** Epigenomic Hits for Limb Congenital Anomalies Extended Exploration, Schrott EWAS Database.

Gene Acronym	Gene Name	Gene Number	Functional Annotation	Status	Page Number	Number of Genes Annotated	*p*-Value
**Meis1**	Meis Homeobox 1	ENSG00000143995		Withdrawal	194	**37**	7.55 × 10^−6^
Meis1	Meis Homeobox 1	ENSG00000143995	Cancer growth	Withdrawal	325	149	7.17 × 10^−6^
Meis1	Meis Homeobox 1	ENSG00000143995	Sensory organ development	Withdrawal	327	18	1.30 × 10^−5^
Meis1	Meis Homeobox 1	ENSG00000143995	Eye formation	Withdrawal	328	15	2.81 × 10^−5^
Meis1	Meis Homeobox 1	ENSG00000143995	Cancer	Withdrawal	329	151	4.32 × 10^−5^
Meis1	Meis Homeobox 1	ENSG00000143995	Lens formation	Withdrawal	333	4	9.17 × 10^−5^
Meis1	Meis Homeobox 1	ENSG00000143995	Cancer	Withdrawal	334	88	1.22 × 10^−4^
Meis1	Meis Homeobox 1	ENSG00000143995	Eye formation	Withdrawal	334	11	1.23 × 10^−4^
**Meis2**	Meis Homeobox 2	ENSG00000134138		Withdrawal	134	**97**	2.36 × 10^−7^
Meis2	Meis Homeobox 2	ENSG00000134138		Withdrawal	181	1	0.016676
Meis2	Meis Homeobox 2	ENSG00000134138		Withdrawal	209	1	0.023289
Meis2	Meis Homeobox 2	ENSG00000134138	Upper Aerodigestive SCC	Withdrawal	325	40	1.28 × 10^−6^
Meis2	Meis Homeobox 2	ENSG00000134138	Upper Aerodigestive SCC	Withdrawal	325	53	3.59 × 10^−6^
Meis2	Meis Homeobox 2	ENSG00000134138	Cranial nerve abnormality	Withdrawal	325	7	6.34 × 10^−6^
Meis2	Meis Homeobox 2	ENSG00000134138	Cancer	Withdrawal	325	149	7.17 × 10^−6^
**FGFs**	Fibroblast Growth Factor			Withdrawal		**175**	
FGFR1OP	FGF Receptor 1 Oncogene Partner	ENSG00000213066		Withdrawal	13	1	0.002226
FGF5	Fibroblast Growth Factor 5	ENSG00000138675		Withdrawal	21	1	0.004362
FGF14	Fibroblast Growth Factor 14	ENSG00000102466		Withdrawal	25	1	0.005329
FGFR2	Fibroblast Growth Factor Receptor 2	ENSG00000066468		Withdrawal	28	1	0.005981
FGF14	Fibroblast Growth Factor 14	ENSG00000102466		Dependence	30	1	8.68 × 10^−7^
FGF12	Fibroblast Growth Factor 12	ENSG00000114279		Dependence	41	1	0.009199
FGF12	Fibroblast Growth Factor 12	ENSG00000114279		Dependence	54	1	0.001187
FGF3	Fibroblast Growth Factor 3	ENSG00000186895		Dependence	81	1	0.017663
FGFRL1	FGF Receptor Like 3	ENSG00000127418		Dependence	86	1	0.018855
FGF14	Fibroblast Growth Factor 14	ENSG00000102466		Dependence	106	1	0.002259
**FGF4**	Fibroblast Growth Factor 4	ENSG00000122642		Dependence	17	**7**	2.34 × 10^−7^
FGF4	Fibroblast Growth Factor 4	ENSG00000122642	KEGG: Rap1 signaling		236	41	0.000353
FGF4	Fibroblast Growth Factor 4	ENSG00000122642	KEGG: actin cytoskeleton		237	37	0.004586
FGF4	Fibroblast Growth Factor 4	ENSG00000122642	KEGG: melanoma		237	15	0.021590
FGF4	Fibroblast Growth Factor 4	ENSG00000122642	KEGG: MAP kinase pathway		237	39	0.029222
FGF4	Fibroblast Growth Factor 4	ENSG00000122642	KEGG: Cancer pathways		238	54	0.067770
FGF4	Fibroblast Growth Factor 4	ENSG00000122642	KEGG: Ras signaling		328	38	0.008745
**RXRA**	Retinoid X Receptor Alpha	ENSG00000186350		Withdrawal	125	1	1.48 × 10^−8^
RXRG	Retinoid X Receptor Gamma	ENSG00000143171		Withdrawal	136	1	3.40 × 10^−7^
RXRA	Retinoid X Receptor Alpha	ENSG00000186350		Withdrawal	144	1	8.40 × 10^−7^
RARA	Retinoic Acid Receptor Alpha	ENSG00000131759		Dependence	44	1	1.95 × 10^−6^
RARB	Retinoic Acid Receptor Beta	ENSG00000077092		Dependence	73	1	5.54 × 10^−6^
RARB	Retinoic Acid Receptor Beta	ENSG00000077092		Withdrawal	124	1	7.94 × 10^−9^
RARB	Retinoic Acid Receptor Beta	ENSG00000077092		Withdrawal	168	1	3.25 × 10^−6^
RARB	Retinoic Acid Receptor Beta	ENSG00000077092		Withdrawal	190	1	6.89 × 10^−6^
RARB	Retinoic Acid Receptor Beta	ENSG00000077092		Withdrawal	215	1	1.20 × 10^−5^
RARA	Retinoic Acid Receptor Alpha	ENSG00000131759	KEGG: Cancer pathways		238	54	0.067777
**WNT’s**	Wnt’s			Withdrawal		**203**	
WNT7B	Wnt family member 7B	ENSG00000188064		Dependence	74	1	5.78 × 10^−6^
WNT7A	Wnt family member 7A	ENSG00000154764		Dependence	119	1	1.47 × 10^−0^
WNT7A	Wnt family member 7A	ENSG00000154764		Dependence	123	1	4.13 × 10^−9^
WNT3A	Wnt family member 3A	ENSG00000154342	Head and neck cancer	Withdrawal	239	356	7.73 × 10^−20^
WNT8B	Wnt family member 8B	ENSG00000075290	Head and neck cancer	Withdrawal	239	342	7.74 × 10^−20^
TBX4	T-Box transcription factor 4	ENSG00000121075		Dependence	52	1	2.72 × 10^−6^
TBX4	T-Box transcription factor 4	ENSG00000121075		Withdrawal	235	1	1.71 × 10^−5^
TBX5-AS1	T-Box transcription factor 5 Antisense 1	ENSG00000255399		Withdrawal	202	1	9.18 × 10^−6^
CHD7	Chromodomain Helicase DNA Binding Protein 7	ENSG00000171316		Dependence	37	**124**	1.37 × 10^−6^
CHD7	Chromodomain Helicase DNA Binding Protein 7	ENSG00000171316	Upper aerodigestive SCC	Withdrawal	325	40	1.28 × 10^−6^
CHD7	Chromodomain Helicase DNA Binding Protein 7	ENSG00000171316	Upper aerodigestive SCC	Withdrawal	325	115	1.65 × 10^−6^
CHD7	Chromodomain Helicase DNA Binding Protein 7	ENSG00000171316	Skin lesion	Withdrawal	325	53	3.59 × 10^−6^
CHD7	Chromodomain Helicase DNA Binding Protein 7	ENSG00000171316	Skin cancer	Withdrawal	325	113	4.79 × 10^−6^
CHD7	Chromodomain Helicase DNA Binding Protein 7	ENSG00000171316	Cancer	Withdrawal	325	149	7.17 × 10^−6^
CHD7	Chromodomain Helicase DNA Binding Protein 7	ENSG00000171316	Large bowel adenocarcinoma	Withdrawal	326	120	7.45 × 10^−6^
CHD7	Chromodomain Helicase DNA Binding Protein 7	ENSG00000171316	Cutaneous melanoma	Withdrawal	326	110	7.71 × 10^−6^
CHD7	Chromodomain Helicase DNA Binding Protein 7	ENSG00000171316	High grade astocytoma	Withdrawal	326	82	8.42 × 10^−6^
CHD7	Chromodomain Helicase DNA Binding Protein 7	ENSG00000171316	Abdominal adenocarcinoma	Withdrawal	326	135	8.46 × 10^−6^
CHD7	Chromodomain Helicase DNA Binding Protein 7	ENSG00000171316	Solid organ cancer	Withdrawal	327	150	9.16 × 10^−6^
CHD7	Chromodomain Helicase DNA Binding Protein 7	ENSG00000171316	Head and neck cancer	Withdrawal	327	137	9.54 × 10^−6^
CHD7	Chromodomain Helicase DNA Binding Protein 7	ENSG00000171316	Sensory organ development	Withdrawal	327	18	1.30 × 10^−5^
CHD7	Chromodomain Helicase DNA Binding Protein 7	ENSG00000171316	Carcinoma	Withdrawal	327	148	1.38 × 10^−5^
CHD7	Chromodomain Helicase DNA Binding Protein 7	ENSG00000171316	Upper aerodigestive SCC	Withdrawal	327	44	1.60 × 10^−43^
MEGF8	Multiple EGF-like domains 8	ENSG00000105429	Skin lesion	Withdrawal	325	**105**	1.65 × 10^−6^
MEGF8	Multiple EGF-like domains 8	ENSG00000105429	Skin cancer	Withdrawal	325	113	4.79 × 10^−6^
MEGF8	Multiple EGF-like domains 8	ENSG00000105429	Cranial nerve abnormality	Withdrawal	325	7	6.34 × 10^−6^
MEGF8	Multiple EGF-like domains 8	ENSG00000105429	Cancer	Withdrawal	325	149	7.17 × 10^−6^
MEGF8	Multiple EGF-like domains 8	ENSG00000105429	Large bowel adenocarcinoma	Withdrawal	326	120	7.45 × 10^−6^
MEGF8	Multiple EGF-like domains 8	ENSG00000105429	Cutaneous melanoma	Withdrawal	326	110	7.71 × 10^−6^
MEGF8	Multiple EGF-like domains 8	ENSG00000105429	High grade astocytoma	Withdrawal	326	82	8.42 × 10^−6^
MEGF8	Multiple EGF-like domains 8	ENSG00000105429	Abdominal adenocarcinoma	Withdrawal	326	135	8.46 × 10^−6^
MEGF8	Multiple EGF-like domains 8	ENSG00000105429	Solid organ cancer	Withdrawal	327	150	9.16 × 10^−6^
MEGF8	Multiple EGF-like domains 8	ENSG00000105429	Head and neck cancer	Withdrawal	327	137	9.54 × 10^−6^
MEGF8	Multiple EGF-like domains 8	ENSG00000105429	Carcinoma	Withdrawal	327	148	1.38 × 10^−5^
MEGF8	Multiple EGF-like domains 8	ENSG00000105429	Carcinoma	Withdrawal	329	151	4.32 × 10^−5^
MEGF8	Multiple EGF-like domains 8	ENSG00000105429	Squamous cell tumor	Withdrawal	332	65	7.59 × 10^−5^
MEGF8	Multiple EGF-like domains 8	ENSG00000105429	Preaxial polydactyly	Withdrawal	333	3	9.19 × 10^−5^
TMEM107	Transmembrane protein 107	ENSG00000179029	Upper aerodigestive SCC	Withdrawal	325	**22**	1.28 × 10^−6^
TMEM107	Transmembrane protein 107	ENSG00000179029	Cancer	Withdrawal	325	149	7.17 × 10^−6^
TMEM107	Transmembrane protein 107	ENSG00000179029	Solid organ cancer	Withdrawal	327	150	9.16 × 10^−6^
TMEM107	Transmembrane protein 107	ENSG00000179029	Head and neck cancer	Withdrawal	327	137	9.54 × 10^−6^
TMEM107	Transmembrane protein 107	ENSG00000179029	Carcinoma	Withdrawal	327	148	1.38 × 10^−5^
TMEM107	Transmembrane protein 107	ENSG00000179029	Carcinoma	Withdrawal	329	151	4.32 × 10^−5^
TMEM107	Transmembrane protein 107	ENSG00000179029	Squamous cell tumor	Withdrawal	331	65	7.59 × 10^−5^
TMEM107	Transmembrane protein 107	ENSG00000179029	Preaxial polydactyly	Withdrawal	333	3	9.19 × 10^−5^
TMEM107	Transmembrane protein 107	ENSG00000179029	Squamous cell tumor	Withdrawal	334	64	1.45 × 10^−4^
TMEM107	Transmembrane protein 107	ENSG00000179029	Head and neck cancer	Withdrawal	335	127	1.75 × 10^−4^
TMEM107	Transmembrane protein 107	ENSG00000179029	Cancer	Withdrawal	337	79	2.83 × 10^−4^
TMEM107	Transmembrane protein 107	ENSG00000179029	Head abnormalities	Withdrawal	338	21	3.27 × 10^−4^
TMEM107	Transmembrane protein 107	ENSG00000179029	Haemopoietic stimulation	Withdrawal	338	23	3.51 × 10^−4^
BMP4	Bone morphogenetic protein 4	ENSG00000125378	Upper aerodigestive SCC	Withdrawal	325	**166**	1.28 × 10^−6^
BMP4	Bone morphogenetic protein 4	ENSG00000125378	Upper aerodigestive SCC	Withdrawal	325	115	1.65 × 10^−6^
BMP4	Bone morphogenetic protein 4	ENSG00000125378	Cranial nerve abnormality	Withdrawal	325	7	6.34 × 10^−6^
BMP4	Bone morphogenetic protein 4	ENSG00000125378	Cancer	Withdrawal	325	149	7.17 × 10^−6^
BMP4	Bone morphogenetic protein 4	ENSG00000125378	Large bowel adenocarcinoma	Withdrawal	326	120	7.45 × 10^−6^
BMP4	Bone morphogenetic protein 4	ENSG00000125378	Abdominal adenocarcinoma	Withdrawal	326	135	8.46 × 10^−6^
BMP4	Bone morphogenetic protein 4	ENSG00000125378	Solid organ cancer	Withdrawal	327	150	9.16 × 10^-=6^
BMP4	Bone morphogenetic protein 4	ENSG00000125378	Head and neck cancer	Withdrawal	327	137	9.54 × 10^−6^
BMP4	Bone morphogenetic protein 4	ENSG00000125378	Sensory organ development	Withdrawal	327	18	1.30 × 10^−5^
BMP4	Bone morphogenetic protein 4	ENSG00000125378	Carcinoma	Withdrawal	327	148	1.38 × 10^−5^
BMP4	Bone morphogenetic protein 4	ENSG00000125378	Upper aerodigestive SCC	Withdrawal	327	44	1.60 × 10^−5^
BMP4	Bone morphogenetic protein 4	ENSG00000125378	Carcinoma	Withdrawal	328	119	2.47 × 10^−5^
BMP4	Bone morphogenetic protein 4	ENSG00000125378	Eye formation	Withdrawal	328	15	2.81 × 10^−5^
BMP4	Bone morphogenetic protein 4	ENSG00000125378	Upper GIT carcinoma	Withdrawal	328	75	3.42 × 10^−5^
BMP4	Bone morphogenetic protein 4	ENSG00000125378	GIT adenocarcinoma	Withdrawal	328	121	3.56 × 10^−5^
GREM1	GREM1, DAN family BMP antagonist	ENSG00000126873		Withdrawal	171	1	3.61 × 10^−6^
GREM2	GREM2, DAN family BMP antagonist	ENSG00000180875		Withdrawal	85	1	9.90 × 10^−6^
GLI3	GLI zinc finger family 3	ENSG00000106571	Skin lesion	Withdrawal	325	**183**	1.28 × 10^−6^
GLI3	GLI zinc finger family 3	ENSG00000106571	Head and neck squamous carcinoma	Withdrawal	325	53	1.65 × 10^−6^
GLI3	GLI zinc finger family 3	ENSG00000106571	Skin cancer	Withdrawal	325	113	3.59 × 10^−6^
GLI3	GLI zinc finger family 3	ENSG00000106571	Lung adenocarcinoma	Withdrawal	325	42	4.79 × 10^−6^
GLI3	GLI zinc finger family 3	ENSG00000106571	Cancer	Withdrawal	325	149	7.17 × 10^−6^
GLI3	GLI zinc finger family 3	ENSG00000106571	Large bowel adenocarcinoma	Withdrawal	326	120	7.45 × 10^−6^
GLI3	GLI zinc finger family 3	ENSG00000106571	Cutaneous melanoma	Withdrawal	326	110	7.71 × 10^−6^
GLI3	GLI zinc finger family 3	ENSG00000106571	High grade astocytoma	Withdrawal	326	82	8.42 × 10^−6^
GLI3	GLI zinc finger family 3	ENSG00000106571	Abdominal adenocarcinoma	Withdrawal	326	135	8.46 × 10^−6^
GLI3	GLI zinc finger family 3	ENSG00000106571	Solid organ cancer	Withdrawal	327	150	9.16 × 10^−6^
GLI3	GLI zinc finger family 3	ENSG00000106571	Head and neck cancer	Withdrawal	327	137	9.54 × 10^−6^
GLI3	GLI zinc finger family 3	ENSG00000106571	Sensory organ development	Withdrawal	327	18	1.30 × 10^−5^
GLI3	GLI zinc finger family 3	ENSG00000106571	Carcinoma	Withdrawal	327	148	1.38 × 10^−5^
GLI3	GLI zinc finger family 3	ENSG00000106571	Upper aerodigestive SCC	Withdrawal	327	44	1.60 × 10^−43^

Key: The first entry in each type of gene is in bold. This signifies the gene class. Its initial entry signifies the number of entries for that gene in the data set.

**Table 8:** Summary Epigenomic Hits for All Congenital Anomalies.by Organ System, Schrott EWAS Database.

System	Mean *p*-Value	Median *p*-Value
Gastrointestinal	0.0011	7.45 × 10^−6^
Chromosomes	0.0018	1.31 × 10^−4^
Neurological	0.0035	6.15 × 10^−4^
Cardiovascular	0.0011	0.0011
Face	0.0021	0.0014
Body Wall	0.0018	0.0016
General	0.0026	0.0017
Uronephrology	0.0021	0.0022
Limb	0.0036	0.0037

**Table 9:** Contrast of Epigenomic Hits for All Congenital Anomalies.by Organ Target, Cannabis Dependence vs. Withdrawal, Schrott EWAS Database.

Target	Cannabis Dependence				Cannabis Withdrawal			
	Number of Annotations	Cumulative Genes	Minimum *p*-Value	Median *p*-Value	Number of Annotations	Cumulative Genes	Minimum *p*-Value	Median *p*-Value
Gastrointestinal	8	2561	4.60 × 10^−16^	1.13 × 10^−15^	-	-	-	-
Large Intestine	5	1240	7.65 × 10^−15^	6.40 × 10^−14^	3	363	7.45 × 10^−6^	6.80 × 10^−5^
Esophagus	4	393	3.15 × 10^−13^	9.40 × 10^−4^	3	69	0.0020	0.0028
Neurological	8	710	5.33 × 10^−8^	4.45 × 10^−4^	1	2	7.20 × 10^−4^	7.20 × 10^−4^
Heart	5	53	8.83 × 10^−8^	1.57 × 10^−4^	-	-	-	-
Liver	2	404	1.28 × 10^−7^	1.79 × 10^−7^	-	-	-	-
Brain	6	750	1.39 × 10^−7^	1.86 × 10^−5^	1	3	1.16 × 10^−4^	1.16 × 10^−4^
Pancreas	8	769	9.10 × 10^−7^	1.25 × 10^−5^	4	112	0.0052	0.0061
Embryo	9	285	8.20 × 10^−6^	3.61 × 10^−4^	-	-	-	-
Atrioventricular valves	3	13	9.04 × 10^−6^	4.00 × 10^−5^	-	-	-	-
Neurons	14	336	9.27 × 10^−6^	1.88 × 10^−4^	3	11	0.0020	0.0031
DNA	12	373	1.50 × 10^−5^	0.0011	5	33	3.58 × 10^−4^	0.0070
Chromosomes	3	16	1.60 × 10^−5^	7.90 × 10^−5^	1	1	0.0070	0.0070
Cardiovascular	4	85	2.10 × 10^−5^	0.0019	-	-	-	-
Synapse	15	308	3.12 × 10^−5^	0.0018	7	36	1.43 × 10^−4^	0.0013
Microtubules	1	58	3.30 × 10^−5^	3.30 × 10^−5^	1	24	0.0045	0.0045
Embryo	6	93	3.60 × 10^−5^	0.0018	2	8	0.0023	0.0046
Ventricle	4	23	5.10 × 10^−5^	6.09 × 10^−4^	-	-	-	-
Body	5	132	7.80 × 10^−5^	0.0016	2	51	1.93 × 10^−4^	3.74 × 10^−4^
Eye	6	65	7.90 × 10^−5^	0.0010	13	73	2.80 × 10^−5^	6.89 × 10^−4^
Cerebrum	2	153	1.20 × 10^−4^	7.35 × 10^−4^	4	22	7.41 × 10^−4^	0.0020
Head	1	47	1.20 × 10^−4^	1.20 × 10^−4^	-	-	-	-
Bone	7	50	1.40 × 10^−4^	0.0018	14	48	1.93 × 10^−4^	0.0070
Sensory	1	29	1.64 × 10^−4^	1.64 × 10^−4^	-	-	-	-
Body Axis	1	1	1.93 × 10^−4^	1.93 × 10^−4^	-	-	-	-
Urinary system	1	17	2.20 × 10^−4^	2.20 × 10^−4^	1	8	0.0044	0.0044
Kidney	5	84	4.29 × 10^−4^	0.0022	1	4	0.0042	0.0042
Breast	1	3	5.73 × 10^−4^	5.73 × 10^−4^	2	9	0.0021	0.0023
Granulocytes	1	3	5.73 × 10^−4^	5.73 × 10^−4^	-	-	-	-
Ear	6	36	7.20 × 10^−4^	0.0021	5	21	1.65 × 10^−4^	8.04 × 10^−4^
Atria	4	15	8.55 × 10^−4^	0.0017	-	-	-	-
Body trunk	1	50	0.0015	0.0015	-	-	-	-
Myogenesis	2	4	0.0018	0.0018	-	-	-	-
Vertebra	1	3	0.0049	0.0049	-	-	-	-
Limb	-	-	-	-	6	18	9.20 × 10^−5^	0.0037
Nose	-	-	-	-	1	3	0.0011	0.0011
Ovarian reserve	-	-	-	-	1	2	0.0031	0.0031
Mitochondria	-	-	-	-	1	1	0.0070	0.0070
Palate	-	-	-	-	1	1	0.0070	0.0070

**Table 10:** Comparative Lists of Significantly Cannabinoid-Associated Cancers in Europe and USA.

No.	Europe			USA			
	Model	Cancer	Minimum *p*-Value	Model	Correlate	Cancer	Minimum *p*-Value
1	Categorical	Acute Lymphoid Leukemia	8.70 × 10^−24^	Categorical	Δ9THC	Acute Lymphoid Leukemia	7.65 × 10^−25^
2	Continuous	Acute Myeloid Leukemia	2.11 × 10^−4^	Categorical	Δ9THC	Acute Myeloid Leukemia	3.11 × 10^−110^
3				Categorical	Cannabidiol	All_Cancer	<2.2 × 10^−320^
4	Categorical	Anus	6.71 × 10^−35^				
5	Categorical	Bladder	<2.2 × 10^−320^	Categorical	Cannabidiol	Bladder	<2.2 × 10^−320^
6	Continuous	Brain.Medulloblastoma	5.64 × 10^−42^	Categorical	Cannabidiol	Brain	5.67 × 10^−33^
7	Categorical	Breast	4.03 × 10^−17^	Categorical	Δ9THC	Breast	8.06 × 10^−146^
8	Continuous	Chronic Lymphoid Leukemia	1.20 × 10^−34^	Categorical	Cannabidiol	Chronic Lymphoid Leukemia	2.98 × 10^−12^
9	Continuous	Chronic Myeloid Leukemia	1.32 × 10^−32^	Categorical	Δ9THC	Chronic Myeloid Leukemia	1.52 × 10^−12^
10	Categorical	Colorectum	6.14 × 10^−242^	Categorical	Cannabidiol	Colorectum	<2.2 × 10^−320^
11	Categorical	Corpus uteri	2.28 × 10^−4^				
12	Categorical	Esophagus	1.12 × 10^−110^	Categorical	Cannabidiol	Esophagus	2.31 × 10^−43^
13	Categorical	Gallbladder	2.24 × 10^−4^				
14	Continuous	Hepatocellular Cancer	2.29 × 10^−42^				
15	Categorical	Hodgkin lymphoma	1.80 × 10^−8^	Categorical	Cannabidiol	Hodgkins	1.22 × 10^−30^
16	Categorical	Kaposi sarcoma	1.16 × 10^−7^	Categorical	Cannabidiol	Kaposi	4.75 × 10^−29^
17	Categorical	Kidney	7.46 × 10^−5^	Continuous	Cannabinol	Kidney	0.0067
18	Categorical	Larynx	<2.2 × 10^−320^				
19	Categorical	Liver	<2.2 × 10^−320^	Categorical	Δ9THC	Liver	<2.2 × 10^−320^
20	Categorical	Lung	1.45 × 10^−8^	Categorical	Cannabidiol	Lung	6.87 × 10^−194^
21	Categorical	Melanoma of skin	<2.2 × 10^−320^	Categorical	Cannabidiol	Melanoma	<2.2 × 10^−320^
22	Categorical	Mesothelioma	3.37 × 10^−111^				
23	Categorical	Multiple myeloma	6.92 × 10^−8^	Categorical	Δ9THC	Multiple myeloma	1.73 × 10^−30^
24	Categorical	Non-Hodgkin lymphoma	1.60 × 10^−44^	Categorical	Cannabidiol	Non-Hodgkin lymphoma	3.15 × 10^−145^
25	Continuous	Oropharynx	7.02 × 10^−21^	Continuous	Δ9THC	Oropharynx	3.21 × 10^−6^
26	Categorical	Ovary.Germ Cell Tumor	1.07 × 10^−38^	Categorical	Cannabidiol	Ovary	2.49 × 10^−312^
27	Categorical	Pancreas	4.09 × 10^−9^	Categorical	Δ9THC	Pancreas	4.57 × 10^−166^
28	Categorical	Penis	1.64 × 10^−19^				
29	Categorical	Prostate	<2.2 × 10^−320^	Categorical	Cannabidiol	Prostate	<2.2 × 10^−320^
30				Categorical	Cannabidiol	Stomach	2.30 × 10^−192^
31	Categorical	Testis	3.83 × 10^−81^	Continuous	Cannabinol	Testis	1.47 × 10^−5^
32	Continuous	Testis.Non-Seminoma Germ	1.25 × 10^−75^				
33	Categorical	Testis.Seminoma	5.14 × 10^−58^				
34	Categorical	Thyroid	<2.2 × 10^−320^	Categorical	Δ9THC	Thyroid	<2.2 × 10^−320^
35	Continuous	Vulva	8.88 × 10^−44^				

**Table 11:** Contrast of Cannabis Dependence and Withdrawal Significance Levels and Gene Numbers, Schrott Data.

Cancer	Minimum *p*-Value Dependence	Minimum *p*-Value Withdrawal	*p*-Value Ratio Dependence/Withdrawal	Total Gene Number Dependence	Total Gene Number Withdrawal	Gene Number Ratio Dependence/Withdrawal
Thyroid	1.21 × 10^−17^	0.0014	1.17 × 10^14^	637	115	5.54
Melanoma	3.70 × 10^−15^	7.71 × 10^−6^	2.08 × 10^9^	579	225	2.57
Urinary	2.54 × 10^−14^	2.16 × 10^−4^	8.50 × 10^9^	1191	679	1.75
Esophagus	3.15 × 10^−13^	6.80 × 10^−5^	2.16 × 10^8^	465	117	3.97
Stomach	3.15 × 10^−13^	6.80 × 10^−5^	2.16 × 10^8^	443	102	4.34
Colorectal	7.27 × 10^−13^	6.17 × 10^−4^	8.49 × 10^8^	1734	452	3.84
Testis	1.14 × 10^−8^	6.75 × 10^−4^	5.92 × 10^4^	304	60	5.07
Liver	1.17 × 10^−8^	NA	NA	890	NA	NA
Prostate	2.88 × 10^−8^	5.33 × 10^−4^	1.85 × 10^4^	399	158	2.53
Breast	3.25 × 10^−8^	0.0013	3.91 × 10^4^	674	177	3.81
Brain	5.33 × 10^−8^	8.42 × 10^−6^	157.97	2779	947	2.93
Oropharynx	1.25 × 10^−7^	1.60 × 10^−5^	128.00	195	44	4.43
Pancreas	9.10 × 10^−7^	0.0052	5.73 × 10^3^	769	112	6.87
ALL	4.08 × 10^−5^	6.01 × 10^−4^	14.73	23	118	0.19
NHL	4.08 × 10^−5^	6.11 × 10^−4^	14.98	322	43	7.49
Ovary	1.16 × 10^−4^	0.0070	60.43	529	1	529.00
CML	2.13 × 10^−4^	0.0021	9.95	11	11	1.00
AML	8.96 × 10^−4^	6.26 × 10^−4^	0.70	11	36	0.31
Kidney	0.00101	NA	NA	89	NA	NA
Myeloma	NA	0.0016	NA	NA	10	NA

Key: CML—Chronic Myeloid Leukemia; CLL—Chronic Lymphoid Leukemia; NHL—Non-Hodgkins Lymphoma.

## Data Availability

All data generated or analyzed during this study are included in this published article and its supplementary information files. Data along with the relevant R code have been made publicly available on the Mendeley Database Repository and can be accessed from this URL https://data.mendeley.com/datasets/sngdkpg8gy/1 (doi:10.17632/sngdkpg8gy.1) (accessed on 10 December 2022).

## Supplementary Materials

The following supporting information can be downloaded at: https://www.mdpi.com/article/10.3390/ijerph192416721/s1. Table S1: Histone (Lysine) Methyltransferases; Table S2: Histone (L:ysine) Demethylases; Table S3: Histone Acetyltransferases; Table S4: Histone Deacetylases; Table S5: Other Stem Cell Factors; Table S6: Funtional Annotations of Kit; Table S7: Age-Related Immunometabolic changes; Table S8: Oocyte-Centrosome DNA Methylation Alterations; Table S9: Funtional Annotations of Kinesins; Table S10: Funtional Annotations of Tubulins; Table S11: Funtional Annotations of Tubulins-2; Table S12: Funtional Annotations of CENPN; Table S13: Funtional Annotations of RAD51/52; Table S14: Funtional Annotations of DSCAM; Table S15: Funtional Annotations of DGALP2; Table S16: Funtional Annotations of Slit; Table S17: Funtional Annotations of Robo; Table S18: Funtional Annotations of SRGAP2; Table S19: Funtional Annotations of Alcohol Dehydrogenase (ALDH); Table S20: Funtional Annotations of Retinoid Receptors; Table S21: Funtional Annotations of Notch Receptors; Table S22: Funtional Annotations of VEGF, EFNB2; Table S23: *p*-Values of Teratological Significance from Schrott; Table S24: Annotations from the Schrott Database for Central Nervous System Abnormalities; Table S25: Annotations from the Schrott Database for Cardiovascular System Abnormalities; Table S26: Annotations from the Schrott Database for Orofacial Abnormalities; Table S27: Annotations from the Schrott Database for General Abnormalities; Table S28: Annotations from the Schrott Database for Limb Abnormalities; Table S29: Annotations from the Schrott Database for Gastrointestinal System Abnormalities; Table S30: Annotations from the Schrott Database for Chromomsomal Abnormalities; Table S31: Annotations from the Schrott Database for Uronephrological System Abnormalities; Table S32: Annotations from the Schrott Database for Body Wall Abnormalities; Table S33: Summary of Teratological Findings of Schrott Database by Organ System & Target; Table S34: Summary of Significance of Schrott DNA Methylation hits by Target; Table S35: Significance Levels for Cancers from Schrott Dataset; Table S36: Summary of Significance Levels for Overall Cancers from Schrott Dataset—Ordered by Minimum *p*-Value; Table S37: Summary of Significance Levels for Overall Cancers from Schrott Dataset—Ordered by Median *p*-Value; Table S38: Summary of Significance Levels for Cancers in Cannabis Dependency from Schrott Dataset-Ordered by Minimum *p*-Value; Table S39: Summary of Significance Levels for Cancers in Cannabis Dependency from Schrott Dataset—Ordered by Median *p*-Value; Table S40: Summary of Significance Levels for Cancers in Cannabis Withdrawal from Schrott Dataset-Ordered by Minimum *p*-Value; Table S41: Summary of Significance Levels for Cancers in Cannabis Withdrawal from Schrott Dataset-Ordered by Median *p*-Value; Table S42:Contrast Between Gene Numebrs and Significance Levels in Withdrawal and Dependency Ordered by Gene Number Ratio. Figure S1: Overall results-negative logarithm of *p*-Values for congenital anomalies from Schrott Database by organ system. Figure S2: Overall results-negative logarithm of *p*-Values for congenital anomalies from Schrott Database by organ target. Figure S3: Overall results–Boxplot of negative logarithm of grouped *p*-Values for congenital anomalies from Schrott Database comparing cannabis dependency with cannabis withdrawal. Figure S4: Number of genes annotated in the Schrott database for target organs by dependency status in (A) cannabis dependence and (B) withdrawal. Figure S5: (A) Numbers of gene annotations, (B) numbers of genes affected and (C) negative logarithm of *p*-value by cancer type-overall Schrott data. Figure S6: Direct comparison between *p*-values for cannabis cancer relationships between (A) cannabis dependence and (B) cannabis withdrawal, Schrott data.
