# Supplementary material for: Epigenomic and Other Evidence for Cannabis-Induced Aging Contextualized in a Synthetic Epidemiologic Overview of Cannabinoid-Related Teratogenesis and Cannabinoid-Related Carcinogenesis
Source: Int J Environ Res Public Health. 2022 Dec 13;19(24):16721. doi: 10.3390/ijerph192416721 (PMC9778714; doi:10.3390/ijerph192416721)
Supplement: Supplementary file 1 [file ijerph-19-16721-s001.zip › ijerph-1994175-supplementary/Supplementary Material - Cannabis_Aging.pdf]

SUPPLEMENTARY TABLES

Table of Contents

Table Number	Subject
S1	Histone (Lysine) Methyltransferases
S2	Histone (L:ysine) Demethylases
S3	Histone Acetyltransferases
S4	Histone Deacetylases
S5	Other Stem Cell Factors
S6	Funtional Annotations of Kit
S7	Age-Related Immunometabolic changes
S8	Oocyte-Centrosome DNA Methylation Alterations
S9	Funtional Annotations of Kinesins
S10	Funtional Annotations of Tubulins
S11	Funtional Annotations of Tubulins - 2
S12	Funtional Annotations of CENPN
S13	Funtional Annotations of RAD51/52
S14	Funtional Annotations of DSCAM
S15	Funtional Annotations of DGALP2
S16	Funtional Annotations of Slit
S17	Funtional Annotations of Robo
S18	Funtional Annotations of SRGAP2
S19	Funtional Annotations of Alcohol Dehydrogenase (ALDH)
S20	Funtional Annotations of Retinoid Receptors
S21	Funtional Annotations of Notch Receptors
S22	Funtional Annotations of VEGF, EFNB2

S23	P-Values of Teratological Significance from Schrott
S24	Annotations from the Schrott Database for Central Nervous System Abnormalities
S25	Annotations from the Schrott Database for Cardiovascular System Abnormalities
S26	Annotations from the Schrott Database for Orofacial Abnormalities
S27	Annotations from the Schrott Database for General Abnormalities
S28	Annotations from the Schrott Database for Limb Abnormalities
S29	Annotations from the Schrott Database for Gastrointestinal System Abnormalities
S30	Annotations from the Schrott Database for Chromomsomal Abnormalities
S31	Annotations from the Schrott Database for Uronephrological System Abnormalities
S32	Annotations from the Schrott Database for Body Wall Abnormalities
S33	Summary of Teratological Findings of Schrott Database by Organ System & Target
S34	Summary of Significance of Schrott DNA Methylation hits by Target
S35	Significance Levels for Cancers from Schrott Dataset
S36	Summary of Significance Levels for Overall Cancers from Schrott Dataset - Ordered by Minimum P-Value
S37	Summary of Significance Levels for Overall Cancers from Schrott Dataset - Ordered by Median P-Value
S38	Summary of Significance Levels for Cancers in Cannabis Dependency from Schrott Dataset - Ordered by Minimum P-Value
S39	Summary of Significance Levels for Cancers in Cannabis Dependency from Schrott Dataset - Ordered by Median P-Value
S40	Summary of Significance Levels for Cancers in Cannabis Withdrawal from Schrott Dataset - Ordered by Minimum P-Value
S41	Summary of Significance Levels for Cancers in Cannabis Withdrawal from Schrott Dataset - Ordered by Median P-Value
S42	Contrast Between Gene Numebrs and Significance Levels in Withdrawal and Dependency Ordered by Gene Number Ratio

Supplementary Table S1.: Cannabis Impacts on Histone Methyl transferase Enzymes, , Schrott EWAS Data.

Nearest Gene Name	Nearest Gene Number	Status	Page in Supplement	Chromosome Number	Relative Location	Distance to Nearest Gene (Bases)	P-Value	Bonferroni-Adjusted P-Value
KMT2C	ENSG00000055609	Withdrawal	168	7	Intron	0	3.28E-06	0.013565
CAMKMT	ENSG00000143919	Withdrawal	175	2	Intron	0	4.20E-06	0.015130
KMT5B	ENSG00000055609	Withdrawal	211	11	Intron	0	1.10E-05	0.023897
KMT2E	ENSG00000055609	Withdrawal	214	7	5UTR	0	1.17E-05	0.024460
KMT2A (MLL1)	ENSG00000055609	Withdrawal	325	11			1.28E-06	-
KMT2A (MLL1)	ENSG00000055609	Withdrawal	325	11			1.65E-06	-
KMT2A (MLL1)	ENSG00000055609	Withdrawal	325	11			3.59E-06	-
KMT2A (MLL1)	ENSG00000055609	Withdrawal	325	11			4.79E-06	-
KMT2A (MLL1)	ENSG00000055609	Withdrawal	325	11			5.84E-06	-
KMT2A (MLL1)	ENSG00000055609	Withdrawal	325	11			7.17E-06	-
KMT2A (MLL1)	ENSG00000055609	Withdrawal	326	11			7.45E-06	-
KMT2A (MLL1)	ENSG00000055609	Withdrawal	326	11			7.71E-06	-
KMT2A (MLL1)	ENSG00000055609	Withdrawal	326	11			8.42E-06	-
KMT2A (MLL1)	ENSG00000055609	Withdrawal	326	11			8.46E-06	-
KMT2A (MLL1)	ENSG00000055609	Withdrawal	326	11			9.16E-06	-
KMT2A (MLL1)	ENSG00000055609	Withdrawal	326	11			9.54E-06	-
KMT2A (MLL1)	ENSG00000055609	Withdrawal	326	11			1.27E-05	-
KMT2A (MLL1)	ENSG00000055609	Withdrawal	326	11			1.38E-05	-
KMT2A (MLL1)	ENSG00000055609	Withdrawal	326	11			1.60E-05	-
KMT2A (MLL1)	ENSG00000055609	Withdrawal	326	11			2.06E-05	-
KMT2A (MLL1)	ENSG00000055609	Withdrawal	326	11			2.47E-05	-
KMT2A (MLL1)	ENSG00000055609	Withdrawal	326	11			3.42E-05	-

Supplementary Table S2.: Cannabis Impacts on Histone Demethylase Enzymes, Schrott EWAS Data.

Nearest Gene Name	Nearest Gene Number	Status	Page in Supplement	Chromosome Number	Relative Location	Distance to Nearest Gene (Bases)	P-Value	Bonferroni-Adjusted P-Value
KDM3B	ENSG00000120733	Dependence	9	5	Intron	0	2.78E-08	0.001068
KDM4C	ENSG00000107077	Dependence	11	9	Intron	0	6.88E-08	0.001754
KDM2A	ENSG00000173120	Dependence	14	11	Intron	0	1.17E-07	0.002352
KDM4B	ENSG00000127663	Dependence	14	19	Intron	0	1.37E-07	0.002528
KDM7A	ENSG00000006459	Dependence	41	7	Intron	0	1.67E-06	0.009025
KDM4B	ENSG00000127663	Dependence	48	19	Intron	0	2.40E-06	0.010755
KDM5A	ENSG00000073614	Dependence	50	12	Intron	0	2.62E-06	0.011194
KDM2A	ENSG00000173120	Dependence	56	11	Intron	0	3.25E-06	0.012379
KDM4C	ENSG00000107077	Dependence	107	9	Intron	0	1.17E-05	0.022772
KDM5A	ENSG00000073614	Dependence	123	12	Intron	0	1.12E-09	0.000216
KDM4C	ENSG00000107077	Withdrawal	128	9	Intron	0	5.49E-08	0.001756
KDM4C	ENSG00000107077	Withdrawal	150	9	Intron	0	1.30E-06	0.008774
KDM4B	ENSG00000127663	Dependence	185	19	Intron	0	5.89E-06	0.017834
KDM4B	ENSG00000127663	Dependence	187	19	Intron	0	6.22E-06	0.018347
KDM2B	ENSG00000098094	Dependence	80	12	Intron	0	8.92E-06	0.021771
KDM5B	ENSG00000117139	Dependence	209	1	Intron	0	1.05E-05	0.023365
KDM7A	ENSG00000006459	Dependence	218	7	Intron	0	1.26E-05	0.025401
KDM3B	ENSG00000123733	Dependence	223	5	Intron	0	1.40E-05	0.026649
KDM1A	ENSG00000103365	Withdrawal	228	16	Intron	0	1.53E-05	0.027842
KDM2B	ENSG00000098094	Dependence	239	12	-	-	7.73E-20	-
KDM2B	ENSG00000098094	Dependence	239	12	-	-	7.74E-20	-

Supplementary Table S3.: Cannabis Impacts on Histone Acetyltransferase Enzymes, Schrott EWAS Data.

**Nearest Gene Name**	**Nearest Gene Number**	**Status**	**Page in Supplement**	**Chromosome Number**	**Relative Location**	**Distance to Nearest Gene (Bases)**	**P-Value**	**Bonferroni-Adjusted P-Value**
KAT2A	ENSG00000108773	Dependence	41	17	Intron	0	1.68E-06	0.009061
KAT6A	ENSG00000083168	Dependence	47	8	Intron	0	2.28E-06	0.010489
KAT6A	ENSG00000083168	Dependence	72	8	Intron	0	5.45E-06	0.015911
KAT6B	ENSG00000156650	Dependence	108	10	Intron	0	1.20E-05	0.023003
KAT6B	ENSG00000156650	Dependence	145	10	Intron	0	8.48E-07	0.007138
KAT2B	ENSG00000083168	Withdrawal	145	3	Intron	0	8.98E-07	0.007355
KAT6B	ENSG00000156650	Withdrawal	157	10	Exon	0	1.93E-06	0.010641
KATNAL1	ENSG00000102781	Withdrawal	169	13	Intron	0	3.35E-06	0.013700
KAT6A	ENSG00000083168	Withdrawal	179	8	Intron	0	4.79E-06	0.016136
KAT6A	ENSG00000083168	Withdrawal	179	8	Intron	0	5.10E-06	0.016631
KAT6A	ENSG00000083168	Withdrawal	195	8	Intron	0	7.81E-06	0.020441

Supplementary Table S4.: Cannabis Impacts on Histone Deacetylase Enzymes, Schrott EWAS Data.

Nearest Gene Name	Nearest Gene Number	Status	Page in Supplement	Chromosome Number	Relative Location	Distance to Nearest Gene (Bases)	P-Value	Bonferroni-Adjusted P-Value
HDAC4	ENSG00000068024	Dependence	23	2	Intron	0	4.48E-07	0.004704
HDAC4	ENSG00000108840	Dependence	78	17	Intron	0	6.30E-06	0.017003
HDAC7	ENSG00000068024	Dependence	84	12	Intron	0	7.25E-06	0.018159
HDAC4	ENSG00000068024	Dependence	115	2	Intron	0	1.36E-05	0.024469
HDAC5	ENSG00000108840	Dependence	117	17	Exon	0	1.42E-05	0.025057
HDAC4	ENSG00000068024	Withdrawal	126	2	Intron	0	3.15E-08	0.001319
HDAC9	ENSG00000048052	Withdrawal	134	7	Intron	0	2.35E-07	0.003687
HDAC9	ENSG00000048052	Withdrawal	138	7	Intron	0	4.44E-07	0.005163
HDAC2-AS2	ENSG00000228624	Withdrawal	154	6	Intron	0	1.61E-06	0.009713
HDAC4	ENSG00000068024	Withdrawal	219	2	Intron	0	1.29E-05	0.025639
HDAC9	ENSG00000048052	Withdrawal	225	7	Intron	0	1.44E-05	0.026972

Supplementary Table S5.: Cannabis Impacts on Other Stem Cell Factors, Schrott EWAS Data.

Nearest Gene Name	Chromosome Number	Nearest Gene Number	Dependency Status	Functional Annotation	Page	Distance from Nearest Gene	Relative Position	P-Value	Bonferroni Adjusted P-Value
**RAS**									
RasL12	15	ENSG00000103710	Dependence	Ras like family 12	17	0	Intron	2.09E-07	0.003191
RRAS2	11	ENSG00000133818	Dependence	Ras related 2	22	0	Intron	4.14E-07	0.004416
RASGEF1B	4	ENSG00000138670	Dependence	Ras GEF Domain Family 1B	23	0	Intron	4.40E-07	0.004689
RASA2	3	ENSG00000155903	Dependence	Ras P21 protein activator	23	0	Intron	4.64E-07	0.004796
RASEF	9	ENSG00000165105	Dependence	Ras and EF-hand containing	26	40281	Downstream	6.31E-07	0.005559
RASGRF1	15	ENSG00000058335	Dependence	Ras Guanine nucleotide Releasing Factor	28	0	Exon	7.30E-07	0.006022
RASD2	22	ENSG00000100302	Dependence	Ras D family member 2	52	21082	Downstream	2.73E-06	0.011384
RASSF3	12	ENSG00000153179	Dependence	Ras Domain Family member 3	56	0	Intron	3.23E-06	0.012342
RASAL3	19	ENSG00000105122	Dependence	Ras Activator like 3	56	0	Intron	3.27E-06	0.012415
RASGEF1A	10	ENSG00000198915	Dependence	Ras GEF Domain Family 1A	78	0	Intron	6.27E-06	0.016972
RRAS	19	ENSG00000126458	Dependence	Ras related	90	0	Intron	8.44E-06	0.019488
RASA2	7	ENSG00000105808	Dependence	Ras P21 protein activator	93	0	Intron	8.91E-06	0.020018
RASGEF1B	4	ENSG00000138670	Dependence	Ras GEF Domain Family 1B	93	0	Intron	9.05E-06	0.020169
RASAL2	1	ENSG00000075391	Dependence	Ras activator like 2	97	0	Intron	9.73E-06	0.020864
RASGRP1	15	ENSG00000172575	Dependence	Ras guanyl releasing protein 2	99	0	Intron	1.01E-05	0.021246
RASGRF2	5	ENSG00000113319	Dependence	Ras guanyl nucleortide releasing factor 2	114	0	Intron	1.33E-05	0.024247
RASAL2	1	ENSG00000075391	Withdrawal	Ras activator like 2	130	0	Intron	1.14E-07	0.002516
RASSF8	12	ENSG00000123094	Withdrawal	Ras Domain Family member 8	132	0	Intron	1.72E-07	0.003135
RASGEF1C	5	ENSG00000146090	Withdrawal	Ras GEF domain Family member 1C	133	0	Intron	1.99E-07	0.003406
**Catenins**									
CTNNBIP1	1	ENSG00000178585	Dependence	β-Catenin binding protein 1	119	0	Intron	1.47E-05	0.025467
CTNNBIP1	1	ENSG00000178585	Withdrawal	β-Catenin binding protein 1	173	0	Intron	3.85E-06	0.014531
CTNNBL1	20	ENSG00000132792	Withdrawal	β-Catenin like 1	216	0	Intron	1.22E-05	0.025042
CTNNBL1	20	ENSG00000132792	Withdrawal	β-Catenin like 1	226	0	Exon	1.48E-05	0.027346
CTNND2	5	ENSG00000169862	Dependence	Δ-Catenin 2	16	0	Intron	1.77E-07	0.002917
CTNND2	5	ENSG00000169862	Dependence	Δ-Catenin 2	239			7.73E-20	
CTNND2	5	ENSG00000169862	Dependence	Δ-Catenin 2	239			7.74E-20	
CTNND2	5	ENSG00000169862	Dependence	Δ-Catenin 2	240			3.34E-19	
CTNND2	5	ENSG00000169862	Dependence	Δ-Catenin 2	240			3.64E-19	
MYBL1	8	ENSG00000185697	Dependence	Myb proto-oncogene like 1	177	0	Intron	4.41E-06	0.015546
**KIT**									
KIT	4	ENSG00000157404	Withdrawal	Kit proto-oncogene	155	0	Intron	1.69E-06	0.009933
KITLG	12	ENSG00000049130	Withdrawal	Kit Ligand	193	0	Intron	7.35E-06	0.019897
GDF3	12	ENSG00000184344	Withdrawal	Growth Differentiation factor 3	224	4362	Upstream	1.41E-05	0.026693
PAX7	1	ENSG00000009709	Dependence	Paired box 7	91	0	Intron	1.13E-05	0.024114
PAX7	1	ENSG00000009709	Dependence	Paired box 7	100	0	Intron	1.02E-05	0.021366
LIN28B	6	ENSG00000187772	Dependence	Lin-28 homologue B	116	0	Intron	1.40E-05	0.024911
LIN28B	6	ENSG00000187772	Withdrawal	Lin-28 homologue B	170	0	Intron	3.42E-06	0.013828

Supplementary Table S6.: Cannabis Impacts on Kit Stem Cell Factors, Selected from 185 Hits, Schrott EWAS Data.

Nearest Gene Name	Page	Functional Annotation	Number Genes Identified	P-Value
KITLG	239	Head and neck cancer	356	7.73E-20
KITLG	239	Head and neck cancer	342	7.74E-20
KITLG	240	Head and neck cancer	333	3.34E-19
KITLG	240	Abdominal carcinoma	362	3.64E-19
KITLG	241	Solid cancer	381	6.93E-18
KITLG	241	Thyroid cancer	318	1.21E-17
KITLG	242	Thyroid cancer	319	1.26E-17
KITLG	242	Endocrine tumour	321	1.44E-17
KITLG	243	Adenocarcinoma	351	1.91E-17
KITLG	243	Secretory cancer	339	1.92E-17
KITLG	244	Abdominal adenocarcinoma	346	3.72E-17
KITLG	244	Malignant cancer	313	5.76E-17
KITLG	245	Carcinoma	376	6.96E-17
KITLG	245	Solid cancer	379	7.21E-17
KITLG	246	Tumour frequency	315	7.77E-17
KITLG	246	Abdominal cancer	364	8.91E-17
KITLG	247	Endocrine tumour	327	1.38E-16
KITLG	247	Carcinoma development	310	1.72E-16

Supplementary Table S7.: Cannabis Impacts on Immunometabolic Stem Cell Factors, Schrott EWAS Data.

Nearest Gene Name	Chromosome Number	Nearest Gene Number	Dependency Status	Functional Annotation	Page	Distance from Nearest Gene	Relative Position	P-Value	Bonferroni Adjusted P-[illegible]
**Calorie Restriction Immunometabolic Mediator**									
PLA2G7	6	ENSG00000146070	Withdrawal	Phospholipase A2 Group VII	213	0	Intron	1.15E-05	0.024308
**CXCL9, Chemokine of Immune Aging Index**									
CXCL13	4	ENSG00000156234	Dependence	CXC Motif chemokine ligand 13	90	0	Intron	8.44E-06	0.019488
CXCL13	4	ENSG00000156234	Withdrawal		213	1069	Downstream	1.14E-05	0.024271
**NAD Metabolism**									
NAMPT	10	ENSG00000229644	Dependence	Nicotinamide Phosphoribosyl Transferase	10	41224	Upstream	5.98E-07	0.005441

Supplementary Table S8.: Cannabis Impacts on Oocyte Meiotic Machinery, Schrott EWAS Data.

Nearest Gene Name	Chromosome Number	Nearest Gene Number	Dependency Status	Functional Annotation	Page	Distance from Nearest Gene	Relative Position	P-Value	Bonferroni Adjusted P-Value
**Centrosomal Organizers**									
NUMA1	11	ENSG00000137497	Withdrawal	Nuclear Mitotic Apparatuis Protein 1	212	0	Exon	1.13E-05	0.024154
CEP152	15	ENSG00000103995	Withdrawal	Centrosomal protein 152	195	0	Intron	7.64E-06	0.020250
CEP55	10	ENSG00000138180	Dependence	Centrosomal protein 55	27	13562	Downstream	6.71E-07	0.005734
**Motor Proteins**									
**Dynein - Dynactin**									
DYNC1H1	14	ENSG00000197102	Withdrawal	Dynein cytoplasmic 1 heavy chain 1	135	0	Exon	2.69E-07	0.003989
DYNC1H1	14	ENSG00000197102	Withdrawal	Dynein cytoplasmic 1 heavy chain 1	150	0	Intron	1.33E-06	0.008933
DYNC1H1	14	ENSG00000197102	Withdrawal	Dynein cytoplasmic 1 heavy chain 1	186	0	Intron	6.04E-06	0.018039
DCTN4	5	ENSG00000132912	Dependence	Dynactin subunit 4	37	0	Intron	1.38E-06	0.008195
DCTN4	5	ENSG00000132912	Withdrawal	Dynactin subunit 4	131	327	Downstream	1.25E-07	0.002634
DCTN4	5	ENSG00000132912	Withdrawal	Dynactin subunit 4	151	0	Intron	1.40E-06	0.009142
DCTN4	5	ENSG00000132912	Withdrawal	Dynactin subunit 4	232	0	Intron	1.63E-05	0.028618
**Kinesin**									
KIFAP3	1	ENSG00000075945	Dependence	Kinesin Associated protein 3	21	0	Intron	3.63E-07	0.004266
KIF26B	1	ENSG00000162849	Dependence	Kinesin family member 26B	41	0	Intron	1.69E-06	0.009091
KIF2A	5	ENSG00000068796	Dependence	Kinesin family member 2A	46	0	Intron	2.20E-06	0.010274
KIF26B	1	ENSG00000162849	Dependence	Kinesin family member 26B	47	0	Intron	2.26E-06	0.010440
KIF27	1	ENSG00000165115	Dependence	Kinesin family member 27	56	0	Exon	3.24E-06	0.012349
KIFC3	16	ENSG00000140859	Withdrawal	Kinesin family member C3	173	0	3 Untranslated region	3.87E-06	0.014555
KIF14	1	ENSG00000118193	Withdrawal	Kinesin family member 14	200	33155	Downstream	8.63E-06	0.021429
KIF13B	1	ENSG00000197892	Dependence	Kinesin family member 13B	60	0	Intron	3.76E-06	0.013269
KIF13B	6	ENSG00000197892	Dependence	Kinesin family member 13B	77	0	Intron	6.16E-06	0.016839
KIF3C	2	ENSG00000137497	Dependence	Kinesin family member 3C	79	0	Intron	6.44E-06	0.017205
KIF26B	1	ENSG00000162849	Dependence	Kinesin family member 26B	83	16530	Downstream	7.21E-06	0.018129
KIFC2	8	ENSG00000167702	Withdrawal	Kinesin family member C2	221	0	Exon	1.35E-05	0.026224

Supplementary Table S9.: Cannabis Impacts on Oocyte Meiotic Machinery,
Selected Kinesin Motors from 218 hits, Schrott EWAS Data.

Nearest Gene Name	Page	Functional Annotation	Number Genes Identified	P-Value
KIF13A	239	Head and neck cancer	356	7.73E-20
KIF26A	239	Head and neck cancer	356	7.73E-20
KIF13A	239	Head and neck cancer	342	7.74E-20
KIF26A	239	Head and neck cancer	342	7.74E-20
KIF13A	240	Head and neck cancer	333	3.34E-19
KIF26A	240	Head and neck cancer	333	3.34E-19
KIF13A	240	Abdominal carcinoma	362	3.64E-19
KIF26A	240	Abdominal carcinoma	362	3.64E-19
KIF13A	241	Solid cancer	381	6.93E-18
KIF26A	241	Solid cancer	381	6.93E-18
KIF13A	241	Thyroid cancer	318	1.21E-17
KIF26A	241	Thyroid cancer	318	1.21E-17
KIF13A	242	Thyroid cancer	319	1.26E-17
KIF26A	242	Thyroid cancer	319	1.26E-17
KIF13A	242	Endocrine tumour	321	1.44E-17
KIF26A	242	Endocrine tumour	321	1.44E-17
KIF13A	243	Adenocarcinoma	351	1.91E-17
KIF26A	243	Adenocarcinoma	351	1.91E-17
KIF13A	243	Secretory Structure carcinoma	339	1.92E-17
KIF26A	243	Secretory Structure carcinoma	339	1.92E-17
KIF13A	244	Abdominal carcinoma	346	3.72E-17
KIF26A	244	Abdominal carcinoma	346	3.72E-17
KIF13A	244	Malignant cancer	313	3.76E-17
KIF26A	244	Malignant cancer	313	3.76E-17
KIF13A	245	Carcinoma	376	6.96E-17
KIF26A	245	Carcinoma	376	6.96E-17
KIF13A	245	Non-melanoma solid carcinoma	379	7.21E-17
KIF26A	245	Non-melanoma solid carcinoma	379	7.21E-17

Supplementary Table S10.: Cannabis Impacts on Tubulin and Tubulin Code Machinery, Schrott EWAS Data.

Nearest Gene Name	Chromosome Number	Nearest Gene Number	Dependency Status	Functional Annotation	Function	Page	Distance from Nearest Gene	Relative Position	P-Value	Bonferroni Adjusted P-
**Tubulins**										
TUBB4A	19	ENSG00000104833	Dependence	Tubulin Beta 4A Class Iva		115	2568	Upstream	1.36E-05	0.024542
TUBB8	10	ENSG00000261456	Withdrawal	Tubulin Beta 8 Class VIII		183	0	Intron	5.43E-06	0.017137
TUBD1	17	ENSG00000108423	Withdrawal	Tubulin Delta 1		224	0	Exon	1.42E-05	0.026777
**Tubulin Acetylation**										
ATAT1	6	ENSG00000137343	Withdrawal	Alpha Tubulin Acetyltransferase 1		212	0	Intron	1.13E-05	0.024125
**Tubulin Post-Transational Modifications**										
**Tyrosinylation / Detyrosinylation**										
TTL	2	ENSG00000144999	Withdrawal	Tubulin Tyrosine Ligase	Tyrosinates detyrosinated α-tubulin	224	0	Intron	1.14E-05	0.026704
VASH2	1	ENSG00000143494	Dependence	Vasohibin 2	Removes C-terminal tyrosine from α-tubulin	17	0	Intron	2.60E-07	0.003175
VASH1	14	ENSG00000071246	Withdrawal	Vasohibin 1		174	0	Untranslated regio	3.98E-06	0.014735
**Polyglutaminylation**										
TTLL7	1	ENSG00000137941	Dependence	Tubulin Tyrosine ligase like 7	Polyglutamate ligation	27	0	Intron	7.11E-07	0.005943
TTLL5	14	ENSG00000119685	Dependence	Tubulin Tyrosine ligase like 5	Initiates polyglutamylation	47	0	Intron	2.24E-06	0.010392
TTLL11	9	ENSG00000175746	Dependence	Tubulin Tyrosine ligase like 11	Polyglutamate ligation of sperm flagella	47	0	Intron	2.25E-06	0.010416
TTLL9	20	ENSG00000131044	Dependence	Tubulin Tyrosine ligase like 9	Polyglutamate ligation of sperm flagella	48	0	Intron	2.34E-06	0.010613
TTLL5	14	ENSG00000071246	Dependence	Tubulin Tyrosine ligase like 5	Initiates polyglutamylation	53	2604	Upstream	2.87E-06	0.011632
TTLL9	20	ENSG00000131044	Dependence	Tubulin Tyrosine ligase like 9	Polyglutamate ligation of sperm flagella	57	0	Intron	3.39E-06	0.012645
TTLL6	17	ENSG00000170703	Dependence	Tubulin Tyrosine ligase like 6	Polyglutamate ligation	57	332	Downstream	3.43E-06	0.012715
TTLL11	9	ENSG00000175746	Dependence	Tubulin Tyrosine ligase like 11	Polyglutamate ligation	72	0	Intron	5.43E-06	0.015901
TTLL10	1	ENSG00000162571	Dependence	Tubulin Tyrosine ligase like 10	Protein glycation	85	0	Intron	7.59E-06	0.018620
TTLL11	9	ENSG00000175746	Dependence	Tubulin Tyrosine ligase like 11	Polyglutamate ligation	95	87764	Downstream	9.38E-06	0.020509
TTLL11	9	ENSG00000175746	Dependence	Tubulin Tyrosine ligase like 11	Polyglutamate ligation	121	0	Intron	1.51E-05	0.025795
TTLL1	22	ENSG00000100271	Withdrawal	TTL Family tubulin Polyglutamylase (	Polyglutamate ligation	157	0	Intron	1.98E-06	0.010753
TTLL10	1	ENSG00000162571	Withdrawal	Tubulin Tyrosine ligase like 10	Protein glycation	179	1682	Downstream	4.86E-06	0.016245
TTLL5	14	ENSG00000119685	Withdrawal	Tubulin Tyrosine ligase like 5	Initiates polyglutamylation	181	0	Intron	5.19E-06	0.016760
TTLL11-IT1	9	ENSG00000237548	Withdrawal	Tubulin Tyrosine ligase like 11 lnc RNA		183	0	Intron	5.38E-06	0.019046
TTLL12	22	ENSG00000100304	Withdrawal	Tubulin Tyrosine ligase like 12	Inhibits detyrosination and polyglutaminylati	230	375	Downstream	1.57E-05	0.028092

Supplementary Table S11.: Cannabis Impacts on β-Tubulin, Selected from 86 hits, Schrott EWAS Data.

Nearest Gene Name	Nearest Gene Full Name	Gene Number	Page	Functional Annotation	Number Genes Identified	P-Value
TUBB6	Tubulin Beta 6 Class V	ENSG00000176014	239	Head and neck cancer	356	7.73E-20
TUBB6	Tubulin Beta 6 Class V	ENSG00000176014	239	Head and neck cancer	342	7.74E-20
TUBB6	Tubulin Beta 6 Class V	ENSG00000176014	240	Head and neck cancer	333	3.34E-19
TUBB6	Tubulin Beta 6 Class V	ENSG00000176014	240	Abdominal carcinoma	362	3.64E-19
TUBB6	Tubulin Beta 6 Class V	ENSG00000176014	241	Solid cancer	381	6.93E-18
TUBB6	Tubulin Beta 6 Class V	ENSG00000176014	241	Thyroid cancer	318	1.21E-17
TUBB6	Tubulin Beta 6 Class V	ENSG00000176014	242	Thyroid cancer	319	1.26E-17
TUBB6	Tubulin Beta 6 Class V	ENSG00000176014	242	Endocrine tumour	321	1.44E-17
TUBB6	Tubulin Beta 6 Class V	ENSG00000176014	243	Adenocarcinoma	351	1.91E-17
TUBB6	Tubulin Beta 6 Class V	ENSG00000176014	243	carcinoma	339	1.92E-17
TUBB6	Tubulin Beta 6 Class V	ENSG00000176014	244	Abdominal carcinoma	346	3.72E-17
TUBB6	Tubulin Beta 6 Class V	ENSG00000176014	244	Malignant cancer	313	3.76E-17
TUBB6	Tubulin Beta 6 Class V	ENSG00000176014	245	Carcinoma	376	6.96E-17
TUBB6	Tubulin Beta 6 Class V	ENSG00000176014	245	carcinoma	379	7.21E-17
TUBB6	Tubulin Beta 6 Class V	ENSG00000176014	246	Cancer frequency	315	7.70E-18
TUBB6	Tubulin Beta 6 Class V	ENSG00000176014	246	Abdominal carcinoma	364	8.91E-17
TUBB6	Tubulin Beta 6 Class V	ENSG00000176014	247	Endocrine tumour	327	1.38E-16
TUBB6	Tubulin Beta 6 Class V	ENSG00000176014	247	Carcinoma development	310	1.72E-16

Supplementary Table S12.: Cannabis Impacts on Centrosomal Protein N,
Selected from 86 hits, Schrott EWAS Data.

Nearest Gene Name	Nearest Gene Number	Chromosome Number	Dependency Status	Functional Annotation	Page	Number Genes Identified	P-Value
CENPN	ENSG00000166451	16	Dependence	Head and Neck Cancer	239	356	7.73E-20
CENPN	ENSG00000166451	16	Dependence	Head and Neck Cancer	239	342	7.74E-20
CENPN	ENSG00000166451	16	Dependence	Head and Neck Cancer	240	333	3.34E-19
CENPN	ENSG00000166451	16	Dependence	Abdominal carcinoma	240	362	3.64E-19
CENPN	ENSG00000166451	16	Dependence	Cancer	241	381	6.93E-18
CENPN	ENSG00000166451	16	Dependence	Thyroid carcinoma	241	318	1.21E-17
CENPN	ENSG00000166451	16	Dependence	Secretory structure cancer	243	339	1.92E-17
CENPN	ENSG00000166451	16	Dependence	Malignant Cancer	244	313	5.67E-17
CENPN	ENSG00000166451	16	Dependence	Carcinoma	245	376	6.96E-17
CENPN	ENSG00000166451	16	Dependence	Non-melanoma solid cancer	245	379	7.21E-17
CENPN	ENSG00000166451	16	Dependence	Frequency of Tumour	246	315	7.73E-17
CENPN	ENSG00000166451	16	Dependence	Abdominal cancer	246	364	8.91E-17
CENPN	ENSG00000166451	16	Dependence	Endocrine gland cancer	247	327	1.38E-16
CENPN	ENSG00000166451	16	Dependence	Cancer development	247	310	1.72E-16
CENPN	ENSG00000166451	16	Dependence	Epithelial Tumourigensis	248	312	1.74E-16
CENPN	ENSG00000166451	16	Dependence	Endocrine cancer	249	323	3.74E-16
CENPN	ENSG00000166451	16	Dependence	Gastrointestinal cancer	249	325	4.60E-16
CENPN	ENSG00000166451	16	Dependence	Abdominal cancer	250	365	5.17E-16
CENPN	ENSG00000166451	16	Dependence	Digestive system cancer	250	349	7.78E-16
CENPN	ENSG00000166451	16	Dependence	Digestive system cancer	251	382	8.26E-16

Supplementary Table S13.: Cannabis Impacts on RAD51/52, Schrott EWAS Data.

Nearest Gene Name	Nearest Gene Number	Status	Page in Supplement	Chromosome Number	Relative Location	Distance to Nearest Gene (Bases)	P-Value	Bonferroni-Adjusted P-Value
**RAD51 - Homologous Recombination**								
RAD51B	ENSG00000182185	Dependence	28	14	Intron	0	7.33E-07	0.006027
RAD51B	ENSG00000182185	Dependence	94	14	Intron	0	9.09E-06	0.020192
RAD51B	ENSG00000182185	Withdrawal	131	14	Intron	0	1.33E-07	0.002722
RAD51B	ENSG00000182185	Withdrawal	135	14	Intron	0	2.98E-07	0.004182
RAD51B	ENSG00000182185	Withdrawal	154	14	Intron	0	1.68E-06	0.009923
RAD51B	ENSG00000182185	Withdrawal	158	14	Intron	0	2.13E-06	0.000899
RAD51B	ENSG00000182185	Withdrawal	174	14	Intron	0	4.02E-06	0.014760
RAD51B	ENSG00000182185	Withdrawal	188	14	Intron	0	6.38E-06	0.018632
RAD51B	ENSG00000182185	Withdrawal	222	14	Intron	0	1.36E-05	0.262866
**RAD52 - Microhomology-Mediated End Joining**								
RAD52	ENSG00000002016	Dependence	102	12	Intron	0	1.07E-05	0.021847

Supplementary Table S14.: Cannabis Impacts on DSCAM, Schrott EWAS Data.

Nearest Gene Name	Chromosome Number	Page	Distance to Nearest Gene	Relative Location	Addictive Status	Nearest Gene Number	P-Value	Bonferroni - Adjusted P-Value
DSCAML1	11	5	0	Intron	Dependence	ENSG00000177103	2.22E-09	0.000267
DSCAM	21	63	0	Intron	Dependence	ENSG00000171587	4.14E-06	0.013908
DSCAM	21	73	0	Intron	Dependence	ENSG00000171587	5.57E-06	0.016085
DSCAM	21	82	0	Intron	Dependence	ENSG00000171587	7.08E-06	0.017985
DSCAM	21	113	28301	Downstream	Dependence	ENSG00000171587	1.31E-05	0.024044
DSCAM	21	114	0	Intron	Dependence	ENSG00000171587	1.35E-05	0.024385
DSCAML1	21	132	0	Intron	Withdrawal	ENSG00000177103	1.47E-07	0.002867
DSCAM	21	163	0	Intron	Withdrawal	ENSG00000171587	2.61E-06	0.012188
DSCAM	21	163	0	Intron	Withdrawal	ENSG00000171587	2.62E-06	0.012197
DSCAM	21	180	0	Intron	Withdrawal	ENSG00000171587	5.00E-06	0.016505
DSCAM	21	181	0	Intron	Withdrawal	ENSG00000171587	5.10E-06	0.016631
DSCAM	21	203	0	Intron	Withdrawal	ENSG00000171587	9.30E-06	0.022132
DSCAM	21	214	0	Intron	Withdrawal	ENSG00000171587	1.16E-05	0.024457
DSCAM	21	230	0	Intron	Withdrawal	ENSG00000171587	1.57E-05	0.028126

Supplementary Table S15.: Cannabis Impacts on DLGAP2, Schrott EWAS Data.

Nearest Gene Name	Chromosome Number	Page	Distance to Nearest Gene	Relative Location	Addictive Status	Nearest Gene Number	P-Value	Bonferroni - Adjusted P-Value
DLGAP2	8	30	0	Intron	Dependence	ENSG00000198101	8.64E-07	0.006523
DLGAP2	8	77	0	Intron	Dependence	ENSG00000198101	6.24E-06	0.016936
DLGAP2	8	123	0	Exon	Withdrawal	ENSG00000198101	1.38E-09	0.000244
DLGAP2	8	147	0	Exon	Withdrawal	ENSG00000198101	1.32E-09	0.000244
DLGAP2	8	163	0	Intron	Withdrawal	ENSG00000198101	1.03E-06	0.007912
DLGAP2	8	197	0	Intron	Withdrawal	ENSG00000198101	8.16E-06	0.020881
DLGAP2	8	214	0	Intron	Withdrawal	ENSG00000198101	1.18E-05	0.024556
DLGAP2	8	230	0	Intron	Withdrawal	ENSG00000198101	1.59E-05	0.028299

Supplementary Table S16.: Cannabis Impacts on Slit Signalling, Schrott EWAS Data.

Nearest Gene Name	Chromosome Number	Page	Number of Annotations	Distance to Nearest Gene	Relative Location	Addictive Status	Nearest Gene Number	P-Value	Bonferroni - Adjusted P-Value
ARHGAP19-SLIT1	10	25	1	0	Intron	Dependence	ENSG00000269891	5.46E-07	0.005200
ARHGAP19-SLIT1	10	207	1	0	Intron	Withdrawal	ENSG00000269891	1.03E-05	0.023094
SLIT2	4	145	1	14853	Downstream	Withdrawal	ENSG00000145147	8.65E-07	0.007217
SLIT3	5	72	1	0	Intron	Dependence	ENSG00000184347	5.44E-06	0.015907
SLITRK1	13	107	162	33311	Downstream	Dependence	ENSG00000178235	1.18E-05	0.022816

Supplementary Table S17.: Cannabis Impacts on Robo, Schrott EWAS Data.

Nearest Gene Name	Chromosome Number	Page	Distance to Nearest Gene	Relative Location	Addictive Status	Nearest Gene Number	P-Value	Bonferroni - Adjusted P-Value
**ROBO1**								
ROBO1	3	22	0	Intron	Dependence	ENCG00000169855	4.08E-07	0.004487
ROBO1	3	34	0	Intron	Dependence	ENCG00000169855	1.10E-06	0.007347
ROBO1	3	179	0	Intron	Withdrawal	ENCG00000169855	4.75E-06	0.016077
ROBO1	3	75	0	Intron	Dependence	ENCG00000169855	5.83E-06	0.016430
ROBO1	3	82	1176	Downstream	Dependence	ENCG00000169855	7.09E-06	0.017986
ROBO1	3	103	0	Intron	Dependence	ENCG00000169855	1.10E-05	0.022061
ROBO1	3	118	90264	Upstream	Dependence	ENCG00000169855	1.46E-05	0.025364
**ROBO2**								
ROBO2	3	8	0	Intron	Dependence	ENCG00000185008	1.72E-08	0.000855
ROBO2	3	17	0	Intron	Dependence	ENCG00000185008	2.30E-07	0.003353
ROBO2	3	139	0	Intron	Withdrawal	ENCG00000185008	4.68E-07	0.005298
ROBO2	3	149	0	Intron	Withdrawal	ENCG00000185008	1.20E-06	0.008443
ROBO2	3	167	0	Intron	Withdrawal	ENCG00000185008	3.15E-06	0.013257
ROBO2	3	66	0	Intron	Dependence	ENCG00000185008	4.52E-06	0.014551
ROBO2	3	200	0	Intron	Withdrawal	ENCG00000185008	8.80E-06	0.021641
ROBO2	3	204	0	Intron	Withdrawal	ENCG00000185008	9.48E-06	0.022315
ROBO2	3	106	0	Intron	Dependence	ENCG00000185008	1.16E-05	0.022679
ROBO2	3	223	0	Intron	Withdrawal	ENCG00000185008	1.40E-05	0.026641
ROBO2	3	149	0	Intron	Withdrawal	ENCG00000185008	1.20E-06	0.084427
**ROBO4**								
ROBO4	11	230	0	Intron	Withdrawal	ENCG00000154133	1.59E-05	0.028295

Nearest Gene Name	Chromosome	Page	Fold Enrichment	Status	Nearest Gene	P-Value	P-Bonferroni	P-False Discovery Rate
ROBO1	3	237	1.7082	Dependence	ENCG00000169855	1.12E-02	0.9504	0.001219
ROBO2	3	237	1.7082	Dependence	ENCG00000185008	1.12E-02	0.9504	0.001219

Supplementary Table S18.: Cannabis Impacts on SRGAP2, Schrott EWAS Data.

Nearest Gene Name	Chromosome Number	Page	Distance to Nearest Gene	Relative Location	Addictive Status	Nearest Gene Number	P-Value	Bonferroni - Adjusted P-Value
**SRGAP2C**								
SRGAP2C	1	131	0	Intron	Withdrawal	ENSG00000171943	1.42E-07	0.002813
SRGAP2C	1	132	0	Intron	Withdrawal	ENSG00000171943	1.77E-07	0.003195
**SRGAP2B**								
SRGAP2B	1	94	0	Intron	Dependence	ENSG00000196369	9.24E-06	0.020367
SRGAP2B	1	151	0	Intron	Withdrawal	ENSG00000196369	1.41E-06	0.009159
SRGAP2B	1	152	0	Intron	Withdrawal	ENSG00000196369	1.46E-06	0.009282
SRGAP2B	1	163	0	Intron	Withdrawal	ENSG00000196369	2.56E-06	0.012098
SRGAP2B	1	174	0	Intron	Withdrawal	ENSG00000196369	3.97E-06	0.014721

Supplementary Table S19.: Cannabis Impacts on ALDH, Schrott EWAS Data.

Nearest Gene Name	Nearest Gene	Status	Page in Supplement	Chromosome Number	Relative Location	Distance to Nearest Gene (Bases)	P-Value	Bonferroni-Adjusted P-Value
ALDH1A1	ENSG00000165092	Dependence	48	9	Intron	0	2.36E-06	0.010662
ALDH1A2	ENSG00000128918	Dependence	55	15	Downstream	672	3.10E-06	0.012043
ALDH1A2	ENSG00000128918	Dependence	77	15	Intron	0	6.23E-06	0.016917
ALDH1L1	ENSG00000144908	Dependence	101	3	Intron	0	1.06E-05	0.021663
ALDH18A1	ENSG00000059573	Withdrawal	138	10	Downstream	283	4.42E-07	0.005163
ALDH18A1	ENSG00000059573	Withdrawal	152	10	Intron	0	1.47E-06	0.009315
ALDH1A2	ENSG00000128918	Withdrawal	157	15	Intron	0	1.96E-06	0.010692
ALDH1A2	ENSG00000128918	Withdrawal	191	15	Intron	0	7.01E-06	0.019426
ALDH1A2	ENSG00000128918	Withdrawal	192	15	Intron	0	7.11E-06	0.019685
ALDH1A2	ENSG00000128918	Withdrawal	196	15	Downstream	38465	7.95E-06	0.020609
ALDH1A1	ENSG00000165092	Withdrawal	234	9	Intron	0	1.70E-05	0.029127
CDH8	ENSG00000150394	Dependence	65	16	Intron	0	4.37E-06	0.014300
PCDH17	ENSG00000118946	Dependence	239	13			7.73E-20	

Supplementary Table S20.: Cannabis Impacts on Retinoid Receptor Signalling, Schrott EWAS Data.

Nearest Gene Name	Nearest Gene Number	Status	Page in Supplement	Chromosome Number	Relative Location	Distance to Nearest Gene (Bases)	P-Value	Bonferroni-Adjusted P-Value
RXRA	ENSG00000261150	Withdrawal	125	9	Intron	0	1.48E-08	0.000884
RXRG	ENSG00000143171	Withdrawal	136	1	Downstream	2668	3.40E-07	0.004522
RXRA	ENSG00000186350	Withdrawal	144	9	Intron	0	8.40E-07	0.007110
RORB	ENSG00000186350	Dependence	88	9	Intron	0	8.03E-06	0.019099
RARB	ENSG00000077092	Dependence	3	73	Intron	0	5.54E-06	0.016036
RARB	ENSG00000077092	Withdrawal	3	73	Intron	0	7.94E-09	0.000631
RARB	ENSG00000077092	Withdrawal	3	73	Intron	0	3.25E-06	0.013509
RARB	ENSG00000077092	Withdrawal	3	73	Intron	0	6.98E-06	0.019332
RARB	ENSG00000077092	Withdrawal	3	73	Intron	0	1.20E-05	0.024854

Supplementary Table S21.: Cannabis Impacts on Notch Signalling, Schrott EWAS Data.

Nearest Gene Name	Nearest Gene Number	Page	Annotation	Chromosome Number	Dependency Status	Distance to Nearest Gene	Relative Position	P-Value	Bonferroni Adjusted P-Value
JAG1	ENSG00000101384	36	Notch Ligand	20	Dependence	0	Intron	1.31E-06	0.007992
NOTCH2	ENSG00000134250	55	Notch Pathway	1	Dependence	0	Intron	3.07E-06	0.007992
NOTCH1	ENSG00000101384	56	Notch Pathway	9	Dependence	0	Intron	3.29E-06	0.012443
NOTCH2	ENSG00000134250	64	Notch Pathway	1	Dependence	0	Intron	4.19E-06	0.013994
NOTCH3	ENSG00000074181	86	Notch Pathway	19	Dependence	3797	Downstream	7.65E-06	0.018677
NOTCH2NLC	ENSG00000286219	165	Notch Pathway	1	Withdrawal	0	Intron	2.85E-06	0.012636
RBPJ	ENSG00000168214	107	Notch Pathway	4	Dependence	0	Intron	1.17E-05	0.022698
RBPJP2	ENSG00000274181	121	Notch Pathway	9	Dependence	19867	Upstream	1.50E-05	0.025700
RBPJ	ENSG00000168214	125	Notch Pathway	4	Withdrawal	0	Intron	1.34E-08	0.000859

Nearest Gene Name	Nearest Gene Number	Page	Annotation	Chromosome Number	Dependency Status	Function	Number Genes Identified	P-Value
PSENEN	ENSG00000205155	325	Skin Lesion	19	Withdrawal	Notch Processing	115	1.65E-06
PSENEN	ENSG00000205155	325	Skin cancer	19	Withdrawal	Notch Processing	113	4.79E-06
PSENEN	ENSG00000205155	326	Cutaneous melanc	19	Withdrawal	Notch Processing	110	7.71E-06
PSENEN	ENSG00000205155	327	Solid Organ	19	Withdrawal	Notch Processing	150	9.16E-06
PSENEN	ENSG00000205155	329	Cancer	19	Withdrawal	Notch Processing	151	4.32E-05
PSENEN	ENSG00000205155	329	Melanoma	19	Withdrawal	Notch Processing	115	6.21E-05
PSENEN	ENSG00000205155	335	Solid Organ	19	Withdrawal	Notch Processing	149	1.63E-04
PSENEN	ENSG00000205155	353	Protein	19	Withdrawal	Notch Processing	4	4.69E-03
PSENEN	ENSG00000205155	356	Organismal death	19	Withdrawal	Notch Processing	39	6.70E-03

Supplementary Table S22.: Cannabis Impacts on VEGF Signalling, Schrott EWAS Data.

Nearest Gene Name	Chromosome Number	Nearest Gene Number	Dependency Status	Functional Annotation	Page	Distance from Nearest Gene	Relative Position	P-Value	Bonferroni Adjusted P-Value
VEGFA	6	ENSG00000112715	Dependence	Vascular Endothelial Growth Factor A	60	0	Exon	3.68E-06	0.013108
EFNB2	8	ENSG00000125266	Withdrawal	Ephrin B2	179	0	Intron	4.86E-06	0.016245

KEGG Pathways

Nearest Gene Name	Page	Functional Annotation	Number Genes Identified	P-Value	Bonferroni Adjusted P-Value
VEFGA	236	Rap1 signalling	41	0.000353	0.092548
VEFGA	236	Focal adhesion	39	0.000934	0.226633
VEFGA	237	Ras signalling	38	0.008745	0.910668
VEFGA	238	Cancer pathways	54	0.0677703	1.000000

SupplementaryTable S23.: Comparative Lists of Significantly Cannabinoid-Associated Congenital Anomalies in Europe and USA

No.	Europe				USA			
	Congenital Anomaly	Term	Model	P_Value	Congenital Anomaly	Term	Model	P_Value
1	Abdominal Wall Defx	pm.Resin.Daily	Categorical	3.01E-120				
2	All Anomalies	Daily_Use	Categorical	<2.2E-320				
3	Amniotic band	pm.Resin.Daily	Categorical	1.09E-47				
4	Anencephalus and similar	Resin_THC	Categorical	1.53E-212				
5	Annular Pancreas	Daily_Use	Categorical	1.52E-13				
6	Anophthalmos	Daily_Use	Categorical	1.06E-06				
7	Ano-rectal atresia and stenosis	pm.Resin.Daily	Categorical	4.03E-39				
8	Anotia	Herb_THC	Categorical	4.63E-13	Anotia/microtia	LM_Cannabis	Continuous	7.57E-04
9	Aortic atresia/interrupted aortic arch	LM.Cann_Resin_THC	Categorical	5.71E-25	Interrupted aortic arch	LM_Cannabis	Continuous	3.40E-06
10	Aortic Valve stenosis / atresia	Herb_THC	Categorical	7.14E-13	Aortic valve stenosis	LM_Cannabis	Continuous	0.0019
11	Arhinencephaly/holoprosencephaly	LM_Herb.Daily	Continuous	0.0052				
12	Arterial Truncus	pm.Herb.Daily	Categorical	9.92E-07				
13	Atrial septal defect (ASD)	Herb_THC	Categorical	<2.2E-320	Atrial septal defect (ASD)	LM_Cannabis	Continuous	0.0378
14	Atrioventricular septal defect (AVSD)	pm.Resin.Daily	Categorical	1.65E-101	Atrioventricular septal defect (AVSD)	LM_Cannabis	Categorical	0.0470
15	Bilateral renal agenesis including Potter syndrome	Herb_THC	Categorical	1.08E-47	Renal agenesis/hypoplasia	LM_Cannabis	Continuous	7.34E-04
16	Bile duct atresia	Daily_Use	Categorical	1.00E-40	Biliary atresia	Cannabidiol_Estimates	Continuous	2.43E-04
17	Bladder Extrophy / Epispadias	pm.Resin.Daily	Categorical	1.56E-18	Bladder extrophy	LM_Cannabis	Continuous	0.0170
18	Choanal Atresia	Herb_THC	Categorical	7.34E-94	Choanal atresia	Δ9THC_Estimates	Continuous	0.0033
19	Chromosomal	Daily_Use	Categorical	<2.2E-320				
20	Cleft lip with or without palate	Herb_THC	Categorical	1.80E-101	Cleft lip with and without cleft palate	Cannabidiol_Estimates	Categorical	0.0159
21	Cleft palate	Herb_THC	Categorical	1.79E-34	Cleft palate alone	LM_Cannabis	Continuous	0.0014
22					Cloacal exstrophy	LM_Cannabis	Categorical	2.13E-86
23	Club foot - talipes equinovarus	Daily_Use	Categorical	4.23E-292	Clubfoot	LM_Cannabis	Continuous	3.16E-05
24	Coarctation Aorta	Daily_Use	Categorical	5.78E-33	Coarctation of the aorta	LM_Cannabis	Categorical	9.74E-45
25	Congenital cataract	Daily_Use	Categorical	4.88E-66	Congenital cataract	LM_Cannabis	Continuous	0.0479
26	Congenital glaucoma	Daily_Use	Categorical	1.52E-43				
27	Congenital Heart	pm.Herb.Daily	Categorical	<2.2E-320				
28	Conjoined twins	Daily_Use	Categorical	8.62E-14				
29	Craniosynostosis	Daily_Use	Categorical	5.72E-155				
30	Cystic adenomatous malformation of lung	Daily_Use	Categorical	4.05E-80				

31	Diaphragmatic Hernia	Daily_Use	Categorical	8.77E-57	Diaphragmatic hernia	LM_Cannabis	Categorical	2.11E-08
32	Digestive system	pm.Herb.Daily	Categorical	1.61E-264				
33	Double outlet right ventricle	pm.Herb.Daily	Categorical	1.28E-46	Double outlet right ventricle	LM_Cannabis	Categorical	7.31E-04
34	Down Syndrome	Daily_Use	Categorical	<2.2E-320	Trisomy 21 (Down syndrome)	LM_Cannabis	Categorical	4.02E-26
35	Duodenal stenosis / atresia	Herb_THC	Categorical	1.50E-10				
36	Ear, face and neck	Daily_Use	Categorical	3.38E-44				
37	Ebstein's Anomaly	pm.Resin.Daily	Categorical	3.23E-17				
38	Edward syndrome / Trisomy 18	Daily_Use	Categorical	<2.2E-320	Edward syndrome / Trisomy 18	LM_Cannabis	Categorical	1.06E-61
39	Encephalocele	pm.Resin.Daily	Categorical	4.76E-21	Encephalocele	LM_Cannabis	Continuous	0.0013
40					Epispadias	LM_Cannabis	Continuous	0.0111
41	Eye	Daily_Use	Categorical	2.27E-175				
42	Fetal alcohol syndrome	pm.Resin.Daily	Categorical	5.88E-57				
43	Gastroschisis	Herb_THC	Categorical	6.55E-39				
44	Genetic syndromes + microdeletions	pm.Herb.Daily	Categorical	1.38E-228	Deletion 22q11.2	LM_Cannabis	Continuous	0.0024
45	Genital	pm.Herb.Daily	Categorical	2.55E-243				
46	Hip dislocation and/or dysplasia	Daily_Use	Categorical	0.00E+00	Congenital hip dislocation	LM_Cannabis	Categorical	7.27E-70
47	Hirschsprung's disease	Daily_Use	Categorical	2.54E-88	Hirschsprung disease (congenital megacolon)	LM_Cannabis	Categorical	6.69E-06
48	Holoprosencephaly / Arhinencephaly	LM_Cannabis	Categorical	1.22E-72	Holoprosencephaly	LM_Cannabis	Categorical	2.90E-12
49	Hydrocephalus	pm.Herb.Daily	Categorical	1.76E-110				
50	Hydronephrosis	Herb_THC	Categorical	0.00E+00				
51	Hypoplastic Left Heart	Daily_Use	Categorical	3.37E-61	Hypoplastic left heart syndrome	LM_Cannabis	Continuous	0.0047
52	Hypoplastic right heart	Resin_THC	Categorical	2.85E-59				
53	Hypospadias	pm.Herb.Daily	Categorical	2.92E-177	Hypospadias	LM_Cannabis	Continuous	1.16E-05
54	Klinefelter syndrome	Daily_Use	Categorical	1.75E-41				
55					Large intestinal and Rectal atresia/stenosis	Cannabidiol_Estimates	Continuous	0.0040
56	Lateral anomalies	LM.Cann_Herb_THC	Categorical	2.36E-48				
57	**Limb anomalies**	pm.Herb.Daily	Categorical	<2.2E-320				
58	**Limb reductions**	Daily_Use	Categorical	8.20E-65	**Limb deficiencies (reduction defects)**	LM_Cannabis	Continuous	0.0134
59					**Lower limb Reduction deformity**	LM_Cannabis	Continuous	0.0420
60	Maternal infections resulting in malformations	Daily_Use	Categorical	4.15E-87				
61	Microphthalmos / Anophthalmos	Daily_Use	Categorical	1.25E-55	Microphthalmos / Anophthalmos	Δ9THC_Estimates	Continuous	0.0045
62	Mitral valve anomalies	pm.Herb.Daily	Categorical	8.99E-58				
63	Multicystic renal dysplasia	pm.Resin.Daily	Categorical	6.70E-251				
64	Nervous system	pm.Herb.Daily	Categorical	<2.2E-320				
65	Neural Tube Defects	Resin_THC	Categorical	9.97E-269				

66					Obstructive genitourinary defect	Cannabidiol_Estimates	Categorical	2.22E-15
67	Oesophageal stenosis / atresia	Daily_Use	Categorical	3.49E-44	Oesophageal atresia/tracheoesophageal fistula	LM_Cannabis	Continuous	4.83E-06
68	Omphalocele	pm.Resin.Daily	Categorical	4.94E-131	Omphalocele	LM_Cannabis	Continuous	0.0025
69	Oro-facial clefts	Herb_THC	Categorical	3.99E-133				
70	Patau syndrome/trisomy 13	Daily_Use	Categorical	1.08E-144	Patau syndrome/trisomy 13	LM_Cannabis	Continuous	2.08E-07
71	PDA as only CHD in term infants (>=37 weeks)	pm.Herb.Daily	Categorical	2.14E-20				
72	Polydactyly	pm.Resin.Daily	Categorical	1.46E-292				
73	Posterior urethral valve and/or prune belly	pm.Resin.Daily	Categorical	1.28E-42	Congenital posterior urethral valves	LM_Cannabis	Continuous	1.18E-04
74	Pulmonary valve atresia	Daily_Use	Categorical	1.42E-27	Pulmonary valve atresia	Cannabidiol_Estimates	Categorical	1.02E-05
75	Pulmonary valve stenosis	Daily_Use	Categorical	2.09E-95				
76	Respiratory	pm.Herb.Daily	Categorical	2.57E-203				
77	Severe CHD	Herb_THC	Categorical	1.81E-317				
78	Severe microcephaly	pm.Herb.Daily	Categorical	3.17E-148				
79	Single ventricle	Daily_Use	Categorical	1.03E-25	Single ventricle	LM_Cannabis	Categorical	0.0060
80	Situs inversus	Daily_Use	Categorical	1.42E-44				
81	Skeletal dysplasias	Daily_Use	Categorical	5.12E-74				
82	Small Intestine stenosis / atresia	pm.Herb.Daily	Categorical	8.23E-31	Small intestinal atresia/stenosis	Cannabidiol_Estimates	Continuous	3.39E-06
83	Spina Bifida	Resin_THC	Categorical	3.93E-84	Spina bifida without anencephalus	Δ9THC_Estimates	Continuous	0.0008
84	Syndactyly	pm.Resin.Daily	Categorical	3.47E-16				
85	Teratogenic syndromes with malformations	Daily_Use	Categorical	1.42E-139				
86	Tetralogy of Fallot	Daily_Use	Categorical	3.12E-47	Tetralogy of Fallot	LM_Cannabis	Continuous	0.0168
87	Total Anomalous Pulmonary Venous Return	Herb_THC	Categorical	4.07E-09	Total anomalous pulmonary venous connection	LM_Cannabis	Continuous	0.0299
88	Transposition of great vessels	Resin_THC	Categorical	9.96E-33	Transposition of great arteries	Cannabidiol_Estimates	Continuous	0.0479
89	Turner syndrome	Daily_Use	Categorical	1.10E-146	Turner syndrome	LM_Cannabis	Categorical	7.69E-49
90	Tricuspid valve stenosis / atresia	Daily_Use	Categorical	6.86E-24				
91	Urinary	pm.Resin.Daily	Categorical	<2.2E-320				
92	Valproate syndrome	Daily_Use	Categorical	1.57E-07				
93	Vascular disruption anomalies	Herb_THC	Categorical	3.46E-101				
94	VATER/VACTERL	pm.Herb.Daily	Categorical	2.43E-36				
95	Ventricular septal defect (VSD)	pm.Resin.Daily	Categorical	<2.2E-320	Ventricular septal defect	LM_Cannabis	Continuous	0.0021

Supplementary Table S24.: Epigenomic Hits for Central Nervous System Congenital Anomalies, Schrott EWAS Database

Target	Annotation	Status	Page	Number of Genes Annotated	P-Value
Brain	Glioma	Dependence	284	227	1.39E-07
	Brain lesion	Dependence	285	230	1.63E-07
	Brain tumour	Dependence	286	228	2.29E-07
	Brain size	Dependence	351	6	0.003630
	Brain formation	Dependence	356	12	0.006790
	Morphology of CNS	Dependence	300	47	0.000037
	Forebrain patterning	Withdrawal	333	3	0.000116
Neurological	Glioma	Dependence	281	235	5.33E-08
	Glioma	Dependence	284	227	1.39E-07
	Brain lesion	Dependence	285	230	1.63E-07
	Morphology enteric ganglia	Dependence	308	3	2.74E-04
	Morphology of spiral ganglia	Dependence	312	4	0.000615
	Morphology of spiral ganglia	Withdrawal	342	2	0.000720
	Sciatic nerve myelination	Dependence	314	3	0.000777
	Hair cell morphology	Dependence	316	5	0.001190
	Stratum pyramidale morphology	Dependence	316	3	0.001310
Synapse	Glutaminergic synapse	Dependence	236	29	3.11E-04
	Neuronal synapse	Dependence	236	53	7.10E-05
	Dopaminergic Synapse	Dependence	236	29	3.12E-05
	Dopaminergic Synapse	Dependence	236	27	0.007806
	Retrograde endocannabinoid signalling	Dependence	236	24	0.000511
	Long term depression	Dependence	236	17	0.000582
	Long term potentiation	Dependence	236	17	0.001768
	Morphine addiction	Dependence	236	20	0.004349
	Serotonergic Synapse	Dependence	237	21	0.018174
	Cholinergic Synapse	Dependence	237	19	0.060483
	GABAergic synapse	Dependence	238	15	0.081486
	Synaptic transmission	Dependence	308	20	0.000291
	Synaptic Organization	Dependence	310	8	0.000338
	Inhibitory post-synaptic potential	Dependence	321	5	0.001890
	Maturation of synapse	Dependence	323	4	0.002480
	Synaptic transmission	Withdrawal	334	12	0.000143
	Long term potentiation excitatory synapses	Withdrawal	337	2	0.000291
	Development of synapses	Withdrawal	344	8	0.001210
	Excitatory post-synaptic potential	Withdrawal	344	6	0.001250
	Synaptic transmission	Withdrawal	346	4	0.002020
	Density of synapse	Withdrawal	352	3	0.004640
	Binding presynaptic membrane	Withdrawal	357	1	0.007010

Cerebrum	Cerebral disorder	Dependence	317	106	0.001350
	Development of Cerebral cortex	Withdrawal	351	6	0.004070
	Head development	Dependence	304	47	0.000120
	Head size	Withdrawal	342	8	0.000741
	Viability of cerebral cortex cells	Withdrawal	349	4	0.002780
	Movement of cerebral cortex cells	Withdrawal	344	4	0.001200
Neurons	Abnormal development of sensory neurons	Dependence	322	7	0.002290
	Neurite growth	Dependence	306	26	0.000155
	Abnormal neuronal morphology	Dependence	306	26	0.000182
	Neuronal growth	Dependence	295	25	0.000014
	Proliferation of neurons	Dependence	296	35	0.000194
	Neuronogenesis	Dependence	299	43	0.000026
	Neuronal Morphology	Dependence	296	35	9.27E-06
	Neuronal development	Dependence	299	43	0.000026
	Neuronal outgrowth	Dependence	298	25	0.000014
	Synapse growth	Dependence	308	27	0.000263
	Axonogenesis	Dependence	318	14	0.001640
	Axon regeneration	Dependence	322	7	0.002170
	Movement of brain cells	Dependence	319	8	0.001740
	Movement of brain cells	Withdrawal	347	5	0.002040
	Neuronogenesis	Withdrawal	349	2	0.003080
	Apoptosis cortical neurons	Withdrawal	356	4	0.006650
	Developmental synapse formation	Dependence	308	15	0.000291
Eye	Eye formation	Dependence	302	25	0.000079
	Eye formation	Withdrawal	328	15	0.000028
	Eye morphology	Dependence	308	18	0.000243
	Eye morphology	Withdrawal	334	11	0.000123
	Eye morphology - Anterior Segment	Dependence	318	7	0.001630
	Eye morphology	Withdrawal	340	9	0.000554
	Eye morphogenesis	Dependence	338	5	0.000390
	Photoreceptor abnormalities	Withdrawal	340	5	0.000611
	Morphogenesis camera type eye	Withdrawal	341	4	0.000689
	Morphology of eye cells	Withdrawal	342	6	0.000856
	Retinal Photoresponse	Withdrawal	354	2	0.005500
	Abnormal Ocular Posterior Segment	Withdrawal	342	2	0.000720

Supplementary Table S25.: Epigenomic Hits for Cardiovascular System Congenital Anomalies, Schrott EWAS Database

Target	Annotation	Status	Page	Number of Genes Annotated	P-Value
Cardiovascular	Morphology of CVS	Dependence	321	39	0.001920
	Abnormal CVS	Dependence	324	35	0.002740
Heart	Quantity of cardiomyocytes	Dependence	321	4	0.001960
	Hypoplasia of heart chamber	Dependence	299	7	0.000021
	Hypoplasia of the heart	Dependence	288	10	8.83E-08
	Hypoplasia of the heart	Dependence	299	7	0.000021
	Cardiogenesis	Dependence	318	26	0.001640
Atria	Atrial Morphogenesis	Dependence	314	4	0.000855
	Morphogenesis of Atrial Septum	Dependence	318	3	0.001650
	Atrial hypoplasia	Dependence	319	2	0.001770
	Abnormal atrial morphology	Dependence	322	6	0.002100
Atrioventricular valves	Abnormalities of atrioventricular cushions	Dependence	296	6	9.04E-06
	Lack of atrioventricular canal cushion	Dependence	300	4	0.000040
	Morphogenesis of AV valve	Dependence	321	3	0.002040
Ventricle	Formation of ventricular septum	Dependence	315	7	0.001060
	Ventricular septal defects, Type 3	Dependence	301	3	0.000051
	Hypoplasia of ventricle	Dependence	306	6	0.000157
	Hypoplasia of trabeculae carnae	Dependence	321	4	0.001960
	Ventricular septum formation	Dependence	315	7	0.001060
Vessels	Angiogenesis	Dependence	289	54	1.73E-06
	CVS Development	Dependence	294	56	7.32E-06
	Vasculogenesis	Dependence	302	42	6.65E-05
	Sprouting angiogenesis	Dependence	324	7	0.002690
	Vascular endothelial cell movement	Dependence	316	13	0.001210
	Endothelial cell migration	Dependence	317	19	0.001450
	Vascular Endothelial cell migration	Dependence	317	12	0.001530
	Early onset hypertension	Dependence	357	1	0.007010
	Blood vessel breakdown	Dependence	357	1	0.007010
	Pulmonary vein angiogenesis	Dependence	357	1	0.007010

Supplementary Table S26.: Epigenomic Hits for Face Congenital Anomalies, Schrott EWAS Database

Target	Annotation	Status	Page Number	Number of Genes Annotated	P-Value
Head	Head development	Dependence	304	47	0.000120
Sensory	Sensory organs	Dependence	306	29	0.000164
Palate	Palate	Withdrawal	358	1	0.007010
Nose	Nose	Withdrawal	343	3	0.001080
Eye	Eye morphology - Anterior Segment	Dependence	318	7	0.001630
	Morphogenesis camera type eye	Withdrawal	341	4	0.000689
	Morphology of eye cells	Withdrawal	342	6	0.000856
	Cornea	Dependence	323	3	0.002470
	Iris Abnormalities	Withdrawal	354	2	0.005500
	Lens	Withdrawal	354	3	0.005510
	Lens	Withdrawal	333	4	0.000092
Ear	Ear	Withdrawal	342	7	0.000804
	Inner ear	Dependence	323	9	0.002470
	Inner ear	Dependence	316	8	0.001650
	Abnormal Morphology of Cochlea	Dependence	324	6	0.002720
	Cochlea	Withdrawal	353	4	0.004870
	Cochlea Duct	Dependence	324	6	0.003620
	Spiral Ganglion	Withdrawal	312	4	0.000720
	Spiral Ganglion - small	Dependence	342	2	0.000720
	Vestibular Ganglion	Withdrawal	318	3	0.000165
	Hair cell morphology disruption	Dependence	316	5	0.001650
	Vestibulocochlear nerve	Withdrawal	344	3	0.001200

Supplementary Table S27.: Epigenomic Hits for General Congenital Anomalies, Schrott EWAS Database

Target	Annotation	Status	Page Number	Number of Genes	P-Value
Embyro	Cell growth	Dependence	295	27	8.20E-06
	Cell growth	Dependence	296	83	0.000011
	Connective tissue growth	Dependence	297	37	0.000012
	Epithelial growth	Dependence	300	30	0.000036
	Embyronic growth	Dependence	305	27	0.001420
	Organismal growth	Dependence	310	39	0.000361
	Morphogensis of embryo	Dependence	310	15	0.003680
	Morphogensis of embryo	Dependence	300	12	0.003580
	Differentiation of embryonic cells	Dependence	316	15	0.001240
DNA	DNA transcription	Dependence	298	89	0.000015
	RNA translation	Dependence	312	71	0.000540
	DNA transcription	Dependence	314	60	0.000765
	Activation endogenous DNA promoter	Dependence	317	49	0.001360
	Inhibition endogenous DNA promoter	Withdrawal	338	26	0.000358
	Activation endogenous DNA promoter	Withdrawal	357	1	0.007010
	DNA replication, recombination and repair	Dependence	317	12	0.001530
	DNA binding	Dependence	323	24	0.002360
	DNA synthesis and repair	Dependence	323	20	0.002590
	GTP Hydrolysis	Withdrawal	344	4	0.001130
	Chromosomal assembly	Withdrawal	358	1	0.007010
	Thymidine catabolism	Withdrawal	344	1	0.007010
	Chromosomal synapsis	Dependence	298	7	0.000016
	Chromosomal homologous pairing	Dependence	302	4	0.000079
	Chromosomal synapsis	Dependence	306	5	0.000182
	DNA recombination	Dependence	317	12	0.001530
	DNA synthesis	Dependence	323	20	0.002590
Mitochondria	Mitochondria	Withdrawal	357	1	0.007010
Microtubules	Microtubule dynamics	Dependence	300	58	0.000033
	Microtubules	Withdrawal	352	24	0.004450
Body trunk	Body trunk	Dependence	317	50	0.001450
Body Axis	Body Axis	Dependence	302	1	0.000193
Ovarian reserve	Diminished ovarian reserve	Withdrawal	349	2	0.003080
Breast	Breast cell morphogenesis	Dependence	312	3	0.000573
	Formation mammary gland	Withdrawal	347	7	0.002060
	Differentiation of mammary cells	Withdrawal	348	2	0.002580
Granulocytes	Growth of colony forming granulocytes	Dependence	312	3	0.000573
Myogenesis	Germ cell tumour	Dependence	320	2	0.001770
	Carcinoma cell lines	Dependence	320	2	0.001770
Vertebra	Vertebral formation	Dependence	353	3	0.004920

Vertebra	Vertebral formation	Dependence	[illegible]	[illegible]	[illegible]
Bone	Bone marow cell homing	Dependence	318	3	0.001650
	Abnormal bone morphology	Dependence	319	20	0.001680
	Bone marrow derived macrophages	Dependence	319	2	0.001770
	Development of bone marrow cell lines	Dependence	323	5	0.002440
	Loss bone tissue	Dependence	323	3	0.002470
	Movement of Bone marrow cells	Dependence	323	4	0.002480
	Abnormal bone morphology	Dependence	324	13	0.000140
	Femoral bone mineral density	Withdraw al	326	4	0.000193
	Endochondral bone ossification	Withdraw al	337	4	0.000277
	Abnormal bone morphology	Withdraw al	339	15	0.000431
	Bone ossification	Withdraw al	343	6	0.001110
	Bone mineralization	Withdraw al	349	6	0.002390
	Bone marrow cell proliferation	Withdraw al	353	5	0.004660
	Bone cell line attachment	Withdraw al	356	1	0.007010
	Bone mineralization of diaphysis	Withdraw al	357	1	0.007010
	Activation bone cell lines	Withdraw al	357	1	0.007010
	Bone mineral content trabecular layer	Withdraw al	357	1	0.007010
	Bone marrow cell spreading	Withdraw al	357	1	0.007010
	Chondrogensis of chondroprogenitors	Withdraw al	357	1	0.007010
	Bone mineralization of metaphysis	Withdraw al	357	1	0.007010
	Bone mineralization of diaphysis	Withdraw al	358	1	0.007010

Supplementary Table S28.: Epigenomic Hits for Limb Congenital Anomalies, Schrott EWAS Database

Target	Annotation	Status	Page Number	Number of Genes Annotated	P-Value
Limb	Limb morphogenesis in embryo	Withdrawal	352	4	0.004190
	Limb morphogenesis in embryo	Withdrawal	354	5	0.005470
	Apoptosis of limb bud cells	Withdrawal	356	1	0.007010
	Preaxial polydactyly	Withdrawal	333	3	0.000092
	Polysyndactyly	Withdrawal	345	2	0.001700
	Polydactyly	Withdrawal	350	3	0.003300

Supplementary Table S29.: Epigenomic Hits for Gastrointestinal Congenital Anomalies, Schrott EWAS Database

Target	Annotation	Status	Page Number	Number of Genes Annotated	P-Value
Gastrointestinal	Gastrointestinal cancer	Dependence	249	325	4.60E-16
	Gastrointestinal cancer	Dependence	250	349	7.70E-16
	Gastrointestinal tumour	Dependence	252	252	1.01E-15
	Gastrointestinal tumour	Dependence	253	333	1.02E-15
	Gastrointestinal cancer	Dependence	253	332	1.24E-15
	Gastrointestinal tumour	Dependence	255	352	1.49E-15
	Gastrointestinal cancer	Dependence	257	312	6.45E-15
	Large Intestinal cancer	Dependence	257	306	7.65E-15
Esophagus	Gastroesophageal cancer	Dependence	262	169	3.15E-13
	Gastroesophageal cancer	Dependence	265	171	1.53E-12
	Esophageal cancer	Dependence	320	22	0.001880
	Esophageal lesion	Dependence	324	31	0.002750
	Esophageal cancer	Withdrawal	347	15	0.002020
	Esophageal lesion	Withdrawal	348	16	0.002770
	Gastroesophageal adenocarcinoma	Withdrawal	354	38	0.005250
Large Intestine	Large Intestinal carcinoma	Dependence	257	306	7.65E-15
	Large Intestinal carcinoma	Dependence	258	304	1.11E-14
	Large Intestinal carcinoma	Dependence	260	314	6.40E-14
	Large Intestinal carcinoma	Dependence	261	313	7.15E-14
	Large Intestinal adenocarcinoma	Withdrawal	326	120	7.45E-06
	Large Intestinal neoplasm	Withdrawal	331	122	0.000068
	Large Intestinal carcinoma	Withdrawal	333	121	0.000108
	Enteric ganglia	Dependence	308	3	0.000274
Liver	Hepatobiliary carcinoma	Dependence	284	176	1.28E-07
	Hepatobiliary carcinoma	Dependence	286	228	2.29E-07
Pancreas	Pancreatobiliary cancer	Dependence	288	124	9.10E-07
	Pancreatic cancer	Dependence	290	181	2.64E-06
	Pancreatic cancer	Dependence	297	90	0.000012
	Pancreatic tumour	Dependence	297	95	0.000012
	Pancreatic tumour	Dependence	298	106	0.000013
	Pancreatic adenocarcinoma	Dependence	300	85	0.000033
	Pancreatic ductal adenocarcinoma	Dependence	301	82	0.000060
	Pancreatic cell line migration	Dependence	323	6	0.002390
	Pancreatic cell line invasion	Withdrawal	353	3	0.005210
	Pancreatic cell line G1 phase	Withdrawal	353	2	0.006200
	Pancreatic cancer	Withdrawal	355	36	0.006460
	Pancreatic cancer	Withdrawal	355	71	0.005920

Supplementary Table S30.: Epigenomic Hits for Chromosomal Congenital Anomalies, Schrott EWAS Database

Target	Annotation	Status	Page Number	Number of Genes Annotated	P-Value
Chromosomes	Homologous pairing of chromosomes	Dependence	302	4	0.000079
	Assembly of Chromosomes	Withdrawal	358	1	0.007010
	Chromosomal synapsis	Dependence	298	7	0.000016
	Chromosomal synapsis	Dependence	306	5	0.000182

Supplementary Table S31.: Epigenomic Hits for Uronephrological Congenital Anomalies, Schrott EWAS Database

Target	Annotation	Status	Page Number	Number of Genes Annotated	P-Value
Urinary system	Urinary system morphology	Dependence	322	17	0.000220
	Familial anopmalies Urinary tract	Withdrawal	352	8	0.004400
Kidney	Renal agenesis	Dependence	311	5	0.000429
	Morphology of renal tubule	Dependence	313	8	0.000606
	Morphology of kidney	Dependence	322	16	0.002160
	Renal lesion	Dependence	322	48	0.002200
	Abnormal Morphology of renal tubule	Dependence	323	7	0.002550
	Proximal tubular toxicity	Withdrawal	352	4	0.004190

Supplementary Table S32.: Epigenomic Hits for Body Wall Congenital Anomalies, Schrott EWAS Database

Target	Annotation	Status	Page Number	Number of Genes Annotated	P-Value
Body	Body trunk development	Dependence	317	50	0.001450
	Body trunk development	Withdrawal	340	26	0.000555
	Body axis development	Dependence	302	50	0.000078
	Body axis development	Withdrawal	336	25	0.000193
	Myogenesis germ cell tumour lines	Dependence	320	2	0.001770
	Myogenesis carcinoma tumour lines	Dependence	320	2	0.001770
	Development of abdomen	Dependence	317	28	0.001620
Embryo	Morphogenesis embryonic tissue	Dependence	300	12	0.000036
	Embryonic growth	Dependence	305	27	0.000142
	Embryonic morphogenesis	Dependence	310	15	0.003680
	Differentiation embryonic cells	Dependence	315	15	0.001240
	Differentiation embryonic stem cells	Dependence	322	8	0.002320
	Embryonic growth	Dependence	324	16	0.002810
	Quantity of embryo	Withdrawal	348	6	0.002320
	Stimulation of embryonic tissue	Withdrawal	356	2	0.006950

Supplementary Table S33.: Summary Teratology Epigenomic Hits for All Congenital Anomalies, Schrott EWAS Database

System	Target	Annotation	Status	Page	Number of Genes Annotated	P-Value
Cardiovascular	Cardiovascular	Morphology of CVS	Dependence	321	39	0.001920
Cardiovascular	Cardiovascular	Morphology of CVS	Dependence	324	35	0.002740
Cardiovascular	Cardiovascular	Quantity of cardiomyocytes	Dependence	321	4	0.001960
Cardiovascular	Cardiovascular	Hypoplasia of heart chamber	Dependence	299	7	0.000021
Cardiovascular	Heart	Hypoplasia of the heart	Dependence	288	10	8.83E-08
Cardiovascular	Heart	Hypoplasia of the heart	Dependence	299	7	0.000021
Cardiovascular	Heart	Hypoplasia of the heart ventricle	Dependence	306	6	0.000157
Cardiovascular	Heart	Hypoplasia of trabeculae carnae	Dependence	321	4	0.001960
Cardiovascular	Heart	Cardiogenesis	Dependence	318	26	0.001640
Cardiovascular	Atria	Atrial Morphogenesis	Dependence	314	4	0.000855
Cardiovascular	Atria	Morphogenesis of Atrial Septum	Dependence	318	3	0.001650
Cardiovascular	Atria	Atrial hypoplasia	Dependence	319	2	0.001770
Cardiovascular	Atria	Abnormal atrial morphology	Dependence	322	6	0.002100
Cardiovascular	Atrioventricular valves	Abnormalities of atrioventricular cushions	Dependence	296	6	9.04E-06
Cardiovascular	Atrioventricular valves	Lack of atrioventricular canal cushion	Dependence	300	4	0.000040
Cardiovascular	Atrioventricular valves	Morphogenesis of AV valve	Dependence	321	3	0.002040
Cardiovascular	Ventricle	Formation of ventricular septum	Dependence	315	7	0.001060
Cardiovascular	Ventricle	Ventricular septal defects, Type 3	Dependence	301	3	0.000051
Cardiovascular	Ventricle	Hypoplasia of ventricle	Dependence	306	6	0.000157
Cardiovascular	Ventricle	Ventricular septum formation	Dependence	315	7	0.001060

Neurological	Brain	Glioma	Dependence	284	227	1.39E-07
Neurological	Brain	Brain lesion	Dependence	285	230	1.63E-07
Neurological	Brain	Brain tumour	Dependence	286	228	2.29E-07
Neurological	Brain	Brain size	Dependence	351	6	0.003630
Neurological	Brain	Brain formation	Dependence	356	12	0.006790
Neurological	Brain	Morphology of CNS	Dependence	300	47	0.000037
Neurological	Brain	Forebrain patterning	Withdrawal	333	3	0.000116
Neurological	Neurological	Glioma	Dependence	281	235	5.33E-08
Neurological	Neurological	Glioma	Dependence	284	227	1.39E-07
Neurological	Neurological	Brain lesion	Dependence	285	230	1.63E-07
Neurological	Neurological	Morphology enteric ganglia	Dependence	308	3	2.74E-04
Neurological	Neurological	Morphology of spiral ganglia	Dependence	312	4	0.000615
Neurological	Neurological	Morphology of spiral ganglia	Withdrawal	342	2	0.000720
Neurological	Neurological	Sciatic nerve myelination	Dependence	314	3	0.000777
Neurological	Neurological	Hair cell morphology	Dependence	316	5	0.001190
Neurological	Neurological	Stratum pyramidale morphology	Dependence	316	3	0.001310
Neurological	Cerebrum	Cerebral disorder	Dependence	317	106	0.001350
Neurological	Cerebrum	Development of Cerebral cortex	Withdrawal	351	6	0.004070
Neurological	Cerebrum	Head development	Dependence	304	47	0.000120
Neurological	Cerebrum	Head size	Withdrawal	342	8	0.000741
Neurological	Cerebrum	Viability of cerebral cortex cells	Withdrawal	349	4	0.002780
Neurological	Cerebrum	Movement of cerebral cortex cells	Withdrawal	344	4	0.001200
Neurological	Neurons	Abnormal development of sensory neurons	Dependence	322	7	0.002290
Neurological	Neurons	Neurite growth	Dependence	306	26	0.000155
Neurological	Neurons	Abnormal neuronal morphology	Dependence	306	26	0.000182
Neurological	Neurons	Neuronal growth	Dependence	295	25	0.000014
Neurological	Neurons	Proliferation of neurons	Dependence	296	35	0.000194
Neurological	Neurons	Neuronogenesis	Dependence	299	43	0.000026
Neurological	Neurons	Neuronal Morphology	Dependence	296	35	9.27E-06

Neurological	Neurons	Neuronal development	Dependence	299	43	0.000026
Neurological	Neurons	Neuronal outgrowth	Dependence	298	25	0.000014
Neurological	Neurons	Synapse growth	Dependence	308	27	0.000263
Neurological	Neurons	Axonogenesis	Dependence	318	14	0.001640
Neurological	Neurons	Axon regeneration	Dependence	322	7	0.002170
Neurological	Neurons	Movement of brain cells	Dependence	319	8	0.001740
Neurological	Neurons	Movement of brain cells	Withdrawal	347	5	0.002040
Neurological	Neurons	Neuronogenesis	Withdrawal	349	2	0.003080
Neurological	Neurons	Apoptosis cortical neurons	Withdrawal	356	4	0.006650
Neurological	Neurons	Developmental synapse formation	Dependence	308	15	0.000291
Neurological	Eye	Eye formation	Dependence	302	25	0.000079
Neurological	Eye	Eye formation	Withdrawal	328	15	0.000028
Neurological	Eye	Eye morphology	Dependence	308	18	0.000243
Neurological	Eye	Eye morphology	Withdrawal	334	11	0.000123
Neurological	Eye	Eye morphology - Anterior Segment	Dependence	318	7	0.001630
Neurological	Eye	Eye morphology	Withdrawal	340	9	0.000554
Neurological	Eye	Eye morphogenesis	Dependence	338	5	0.000390
Neurological	Eye	Photoreceptor abnormalities	Withdrawal	340	5	0.000611
Neurological	Eye	Morphogenesis camera type eye	Withdrawal	341	4	0.000689
Neurological	Eye	Morphology of eye cells	Withdrawal	342	6	0.000856
Neurological	Eye	Retinal Photoresponse	Withdrawal	354	2	0.005500
Neurological	Eye	Abnormal Ocular Posterior Segment	Withdrawal	342	2	0.000720
Face	Head	Head development	Dependence	304	47	0.000120
Face	Sensory	Sensory organs	Dependence	306	29	0.000164
Face	Palate	Palate	Withdrawal	358	1	0.007010
Face	Nose	Nose	Withdrawal	343	3	0.001080
Face	Eye	Eye morphology - Anterior Segment	Dependence	318	7	0.001630
Face	Eye	Morphogenesis camera type eye	Withdrawal	341	4	0.000689
Face	Eye	Morphology of eye cells	Withdrawal	342	6	0.000856

Face	Eye	Cornea	Dependence	323	3	0.002470
Face	Eye	Iris Abnormalities	Withdrawal	354	2	0.005500
Face	Eye	Lens	Withdrawal	354	3	0.005510
Face	Eye	Lens	Withdrawal	333	4	0.000092
Face	Ear	Ear	Withdrawal	342	7	0.000804
Face	Ear	Inner ear	Dependence	323	9	0.002470
Face	Ear	Inner ear	Dependence	316	8	0.001650
Face	Ear	Abnormal Morphology of Cochlea	Dependence	324	6	0.002720
Face	Ear	Cochlea	Withdrawal	353	4	0.004870
Face	Ear	Cochlea Duct	Dependence	324	6	0.003620
Face	Ear	Spiral Ganglion	Withdrawal	312	4	0.000720
Face	Ear	Spiral Ganglion - small	Dependence	342	2	0.000720
Face	Ear	Vestibular Ganglion	Withdrawal	318	3	0.000165
Face	Ear	Hair cell morphology disruption	Dependence	316	5	0.001650
Face	Ear	Vestibulocochlear nerve	Withdrawal	344	3	0.001200
General	Embryo	Cell growth	Dependence	295	27	8.20E-06
General	Embryo	Cell growth	Dependence	296	83	0.000011
General	Embryo	Connective tissue growth	Dependence	297	37	0.000012
General	Embryo	Epithelial growth	Dependence	300	30	0.000036
General	Embryo	Embryonic growth	Dependence	305	27	0.001420
General	Embryo	Organismal growth	Dependence	310	39	0.000361
General	Embryo	Morphogenesis of embryo	Dependence	310	15	0.003680
General	Embryo	Morphogenesis of embryo	Dependence	300	12	0.003580
General	Embryo	Differentiation of embryonic cells	Dependence	316	15	0.001240
General	DNA	DNA transcription	Dependence	298	89	0.000015
General	DNA	RNA transcription	Dependence	312	71	0.000540
General	DNA	DNA transcription	Dependence	314	60	0.000765
General	DNA	Activation endogenous DNA promoter	Dependence	317	49	0.001360
General	DNA	Inhibition endogenous DNA promoter	Withdrawal	338	26	0.000358

General	DNA	Activation endogenous DNA promoter	Withdrawal	357	1	0.007010
General	DNA	DNA replication, recombination and repair	Dependence	317	12	0.001530
General	DNA	DNA binding	Dependence	323	24	0.002360
General	DNA	DNA synthesis and repair	Dependence	323	20	0.002590
General	DNA	GTP Hydrolysis	Withdrawal	344	4	0.001130
General	DNA	Chromosomal assembly	Withdrawal	358	1	0.007010
General	DNA	Thymidine catabolism	Withdrawal	344	1	0.007010
General	DNA	Chromosomal synapsis	Dependence	298	7	0.000016
General	DNA	Chromosomal homologous pairing	Dependence	302	4	0.000079
General	DNA	Chromosomal synapsis	Dependence	306	5	0.000182
General	DNA	DNA recombination	Dependence	317	12	0.001530
General	DNA	DNA synthesis	Dependence	323	20	0.002590
General	Mitochondria	Mitochondria	Withdrawal	357	1	0.007010
General	Microtubules	Microtubule dynamics	Dependence	300	58	0.000033
General	Microtubules	Microtubules	Withdrawal	352	24	0.004450
General	Body trunk	Body trunk	Dependence	317	50	0.001450
General	Body Axis	Body Axis	Dependence	302	1	0.000193
General	Ovarian reserve	Diminished ovarian reserve	Withdrawal	349	2	0.003080
General	Breast	Breast cell morphogenesis	Dependence	312	3	0.000573
General	Breast	Formation mammary gland	Withdrawal	347	7	0.002060
General	Breast	Differentiation of mammary cells	Withdrawal	348	2	0.002580
General	Granulocytes	Growth of colony forming granulocytes	Dependence	312	3	0.000573
General	Myogenesis	Germ cell tumour	Dependence	320	2	0.001770
General	Myogenesis	carcinoma cell lines	Dependence	320	2	0.001770
General	Vertebra	Vertebral formation	Dependence	353	3	0.004920
General	Bone	Bone marrow cell homing	Dependence	318	3	0.001650
General	Bone	Abnormal bone morphology	Dependence	319	20	0.001680
General	Bone	Bone marrow derived macrophages	Dependence	319	2	0.001770
General	Bone	Development of bone marrow cell lines	Dependence	323	5	0.002440

General	Bone	Loss bone tissue	Dependence	323	3	0.002470
General	Bone	Movement of Bone marrow cells	Dependence	323	4	0.002480
General	Bone	Abnormal bone morphology	Dependence	324	13	0.000140
General	Bone	Femoral bone mineral density	Withdrawal	326	4	0.000193
General	Bone	Endochondral bone ossification	Withdrawal	337	4	0.000277
General	Bone	Abnormal bone morphology	Withdrawal	339	15	0.000431
General	Bone	Bone ossification	Withdrawal	343	6	0.001110
General	Bone	Bone mineralization	Withdrawal	349	6	0.002990
General	Bone	Bone marrow cell proliferation	Withdrawal	353	5	0.004660
General	Bone	Bone cell line attachment	Withdrawal	356	1	0.007010
General	Bone	Bone mineralization of diaphysis	Withdrawal	357	1	0.007010
General	Bone	Activation bone cell lines	Withdrawal	357	1	0.007010
General	Bone	Bone mineral content trabecular layer	Withdrawal	357	1	0.007010
General	Bone	Bone marrow cell spreading	Withdrawal	357	1	0.007010
General	Bone	Chondrogenesis of chondroprogenitors	Withdrawal	357	1	0.007010
General	Bone	Bone mineralization of metaphysis	Withdrawal	357	1	0.007010
General	Bone	Bone mineralization of diaphysis	Withdrawal	358	1	0.007010
Limb	Limb	Limb morphogenesis in embryo	Withdrawal	352	4	0.004190
Limb	Limb	Limb morphogenesis in embryo	Withdrawal	354	5	0.005470
Limb	Limb	Apoptosis of limb bud cells	Withdrawal	356	1	0.007010
Limb	Limb	Preaxial polydactyly	Withdrawal	333	3	0.000092
Limb	Limb	Polysyndactyly	Withdrawal	345	2	0.001700
Limb	Limb	Polydactyly	Withdrawal	350	3	0.003300
Gastrointestinal	Gastrointestinal	Gastrointestinal cancer	Dependence	249	325	4.60E-16
Gastrointestinal	Gastrointestinal	Gastrointestinal cancer	Dependence	250	349	7.70E-16
Gastrointestinal	Gastrointestinal	Gastrointestinal tumour	Dependence	252	252	1.01E-15
Gastrointestinal	Gastrointestinal	Gastrointestinal tumour	Dependence	253	333	1.02E-15
Gastrointestinal	Gastrointestinal	Gastrointestinal cancer	Dependence	253	332	1.24E-15
Gastrointestinal	Gastrointestinal	Gastrointestinal tumour	Dependence	255	352	1.49E-15

Gastrointestinal	Gastrointestinal	Gastrointestinal cancer	Dependence	257	312	6.45E-15
Gastrointestinal	Gastrointestinal	Large Intestinal cancer	Dependence	257	306	7.65E-15
Gastrointestinal	Esophagus	Gastroesophageal cancer	Dependence	262	169	3.15E-13
Gastrointestinal	Esophagus	Gastroesophageal cancer	Dependence	265	171	1.53E-12
Gastrointestinal	Esophagus	Esophageal cancer	Dependence	320	22	0.001880
Gastrointestinal	Esophagus	Esophageal lesion	Dependence	324	31	0.002750
Gastrointestinal	Esophagus	Esophageal cancer	Withdrawal	347	15	0.002020
Gastrointestinal	Esophagus	Esophageal lesion	Withdrawal	348	16	0.002770
Gastrointestinal	Esophagus	Gastroesophageal adenocarcinoma	Withdrawal	354	38	0.005250
Gastrointestinal	Large Intestine	Large Intestinal carcinoma	Dependence	257	306	7.65E-15
Gastrointestinal	Large Intestine	Large Intestinal carcinoma	Dependence	258	304	1.11E-14
Gastrointestinal	Large Intestine	Large Intestinal carcinoma	Dependence	260	314	6.40E-14
Gastrointestinal	Large Intestine	Large Intestinal carcinoma	Dependence	261	313	7.15E-14
Gastrointestinal	Large Intestine	Large Intestinal adenocarcinoma	Withdrawal	326	120	7.45E-06
Gastrointestinal	Large Intestine	Large Intestinal neoplasm	Withdrawal	331	122	0.000068
Gastrointestinal	Large Intestine	Large Intestinal carcinoma	Withdrawal	333	121	0.000108
Gastrointestinal	Large Intestine	Enteric ganglia	Dependence	308	3	0.000274
Gastrointestinal	Liver	Hepatobiliary carcinoma	Dependence	284	176	1.28E-07
Gastrointestinal	Liver	Hepatobiliary carcinoma	Dependence	286	228	2.29E-07
Gastrointestinal	Pancreas	Pancreatobiliary cancer	Dependence	288	124	9.10E-07
Gastrointestinal	Pancreas	Pancreatic cancer	Dependence	290	181	2.64E-06
Gastrointestinal	Pancreas	Pancreatic cancer	Dependence	297	90	0.000012
Gastrointestinal	Pancreas	Pancreatic tumour	Dependence	297	95	0.000012
Gastrointestinal	Pancreas	Pancreatic tumour	Dependence	298	106	0.000013
Gastrointestinal	Pancreas	Pancreatic adenocarcinoma	Dependence	300	85	0.000033
Gastrointestinal	Pancreas	Pancreatic ductal adenocarcinoma	Dependence	301	82	0.000060
Gastrointestinal	Pancreas	Pancreatic cell line migration	Dependence	323	6	0.002390
Gastrointestinal	Pancreas	Pancreatic cell line invasion	Withdrawal	353	3	0.005210
Gastrointestinal	Pancreas	Pancreatic cell line G1 phase	Withdrawal	353	2	0.006200

Gastrointestinal	Pancreas	Pancreatic cancer	Withdrawal	355	36	0.006460
Gastrointestinal	Pancreas	Pancreatic cancer	Withdrawal	355	71	0.005920
Body Wall	Body	Body trunk development	Dependence	317	50	0.001450
Body Wall	Body	Body trunk development	Withdrawal	340	26	0.000555
Body Wall	Body	Body axis development	Dependence	302	50	0.000078
Body Wall	Body	Body axis development	Withdrawal	336	25	0.000193
Body Wall	Body	Myogenesis germ cell tumour lines	Dependence	320	2	0.001770
Body Wall	Body	Myogenesis carcinoma tumour lines	Dependence	320	2	0.001770
Body Wall	Body	Development of abdomen	Dependence	317	28	0.001620
Body Wall	Embryo	Morphogenesis embryonic tissue	Dependence	300	12	0.000036
Body Wall	Embryo	Embryonic growth	Dependence	305	27	0.000142
Body Wall	Embryo	Embryonic morphogenesis	Dependence	310	15	0.003680
Body Wall	Embryo	Differentiation embryonic cells	Dependence	315	15	0.00124
Body Wall	Embryo	Differentiation embryonic stem cells	Dependence	322	8	0.002320
Body Wall	Embryo	Embryonic growth	Dependence	324	16	0.002810
Body Wall	Embryo	Quantity of embryo	Withdrawal	348	6	0.002320
Body Wall	Embryo	Stimulation of embryonic tissue	Withdrawal	356	2	0.006950
Uronephrology	Urinary system	Urinary system morphology	Dependence	322	17	0.000220
Uronephrology	Urinary system	Familial anomalies Urinary tract	Withdrawal	352	8	0.004400
Uronephrology	Kidney	Renal agenesis	Dependence	311	5	0.000429
Uronephrology	Kidney	Morphology of renal tubule	Dependence	313	8	0.000606
Uronephrology	Kidney	Morphology of kidney	Dependence	322	16	0.002160
Uronephrology	Kidney	Renal lesion	Dependence	322	48	0.002200
Uronephrology	Kidney	Abnormal Morphology of renal tubule	Dependence	323	7	0.002550
Uronephrology	Kidney	Proximal tubular toxicity	Withdrawal	352	4	0.004190
Chromosomes	Chromosomes	Homologous pairing of chromosomes	Dependence	302	4	0.000079
Chromosomes	Chromosomes	Assembly of Chromosomes	Withdrawal	358	1	0.007010
Chromosomes	Chromosomes	Chromosomal synapsis	Dependence	298	7	0.000016
Chromosomes	Chromosomes	Chromosomal synapsis	Dependence	306	5	0.000182

Neurological	Synapse	Glutaminergic synapse	Dependence	236	29	3.11E-04
Neurological	Synapse	Neuronal synapse	Dependence	236	53	7.10E-05
Neurological	Synapse	Dopaminergic Synapse	Dependence	236	29	3.12E-05
Neurological	Synapse	Dopaminergic Synapse	Dependence	236	27	0.007806
Neurological	Synapse	Retrograde endocannabinoid signalling	Dependence	236	24	0.000511
Neurological	Synapse	Long term depression	Dependence	236	17	0.000582
Neurological	Synapse	Long term potentiation	Dependence	236	17	0.001768
Neurological	Synapse	Morphine addiction	Dependence	236	20	0.004349
Neurological	Synapse	Serotonergic Synapse	Dependence	237	21	0.018174
Neurological	Synapse	Cholinergic Synapse	Dependence	237	19	0.060483
Neurological	Synapse	GABAergic synapse	Dependence	238	15	0.081486
Neurological	Synapse	Synaptic transmission	Dependence	308	20	0.000291
Neurological	Synapse	Synaptic Organization	Dependence	310	8	0.000338
Neurological	Synapse	Inhibitory post-synaptic potential	Dependence	321	5	0.001890
Neurological	Synapse	Maturation of synapse	Dependence	323	4	0.002480
Neurological	Synapse	Synaptic transmission	Withdrawal	334	12	0.000143
Neurological	Synapse	Long term potentiation excitatory synapses	Withdrawal	337	2	0.000291
Neurological	Synapse	Development of synapses	Withdrawal	344	8	0.001210
Neurological	Synapse	Excitatory post-synaptic potential	Withdrawal	344	6	0.001250
Neurological	Synapse	Synaptic transmission	Withdrawal	346	4	0.002020
Neurological	Synapse	Density of synapse	Withdrawal	352	3	0.004640
Neurological	Synapse	Binding presynaptic membrane	Withdrawal	357	1	0.007010

Supplementary Table S34.: Summary Epigenomic Hits for All Congenital Anomalies by Organ Target, Schrott EWAS Database

Target	Mean P-Value	Median P-Value
Gastrointestinal	2.51E-15	1.13E-15
Liver	1.79E-07	1.79E-07
Large Intestine	5.72E-05	3.73E-06
Brain	0.0015	3.70E-05
Atrioventricular valves	6.96E-04	4.00E-05
Pancreas	0.0022	4.65E-05
Head	1.20E-04	1.20E-04
Chromosomes	0.0018	1.31E-04
Heart	7.56E-04	1.57E-04
Sensory	1.64E-04	1.64E-04
Body Axis	1.93E-04	1.93E-04
Neurons	0.0012	0.0003
Embyro	0.0011	0.0004
Granulocytes	5.73E-04	5.73E-04
Ventricle	5.82E-04	6.09E-04
Neurological	5.43E-04	6.15E-04
Eye	0.0015	0.0007
Nose	0.0011	0.0011
Cerebrum	0.0017	0.0013
DNA	0.0021	0.0014
Body	0.0011	0.0015
Body trunk	0.0015	0.0015
Synapse	0.0090	0.0015
Ear	0.0019	0.0017
Atria	0.0016	0.0017
Myogenesis	0.0018	0.0018
Cardiovascular	0.0017	0.0019
Esophagus	0.0021	0.0020
Breast	0.0017	0.0021
Kidney	0.0020	0.0022
Microtubules	0.0022	0.0022
Urinary system	0.0023	0.0023
Embryo	0.0024	0.0023
Bone	0.0037	0.0025
Ovarian reserve	0.0031	0.0031
Limb	0.0036	0.0037
Vertebra	0.0049	0.0049
Mitochondria	0.0070	0.0070
Palate	0.0070	0.0070

Supplementary Table S35.: P-Values for Cancers from Schrott EWAS Database

Cancer	Code	Status	Page	P_Value	Gene_Number
T-Lymphoblastic Leukaemia / Lymphoma	ALL	Dependence	300	4.08E-05	20
G1/G1 Phase Transition Leukaemia Cell Lines	ALL	Dependence	310	0.000406	3
Philadelphia Chromosome Negative Acute Lymphoblastic Leukaemia	ALL	Withdrawal	340	0.000601	4
B-Lymphoblastic Leukaemia / Lymphoma w Recurrent Genetic Abnormalities	ALL	Withdrawal	340	0.00611	5
Acute Leukemia	ALL	Withdrawal	341	0.000626	36
Precursor B-cell Acute Lymphoblastic Leukaemia	ALL	Withdrawal	341	0.000684	16
Acute Lymphoblastic Leukaemia	ALL	Withdrawal	348	0.00248	17
T-Lymphoblastic Leukaemia / Lymphoma	ALL	Withdrawal	348	0.00257	9
B-Cell Leukaemia	ALL	Withdrawal	349	0.00311	17
Chemotherapy Refractory ALL	ALL	Withdrawal	350	0.00362	2
E2A-PBX B-Cell ALL	ALL	Withdrawal	351	0.00412	3
Philadelphia-Like B-cell ALL	ALL	Withdrawal	352	0.00438	3
KMT2A-Rearrangement B-Cell ALL	ALL	Withdrawal	353	0.00464	3
Philadelphia Positive B-Cell ALL	ALL	Withdrawal	354	0.00582	3
Maturation Acute Myeloblastic Leukaemia Cell Lines	AML	Dependence	314	0.000896	2
Non-M3 Acute Myeloid Leukaemia	AML	Dependence	323	0.00257	9
Acute Leukemia	AML	Withdrawal	341	0.000626	36
Brain glioma	Brain	Dependence	284	1.39E-07	227
Brain tumour	Brain	Dependence	286	2.29E-07	228
High grade Astrocytoma	Brain	Dependence	294	5.95E-06	179
Brain astrocytoma	Brain	Dependence	294	6.45E-06	173
Brain cancer	Brain	Dependence	308	0.000269	98
Brain cell - cell movement	Brain	Dependence	319	0.00174	8

Brain astrocytoma	Brain	Withdrawal	330	5.12E-05	77
Brain tumour	Brain	Withdrawal	337	2.56E-04	94
Brain glioma	Brain	Withdrawal	339	0.00052	92
Brain cell - cell movement	Brain	Withdrawal	347	0.00204	5
Glioma	Brain	Dependence	281	5.33E-08	235
Glioma cancer	Brain	Dependence	282	6.69E-08	231
Brain glioma	Brain	Dependence	284	1.39E-07	227
Grade 3-4 Glioma cancer	Brain	Dependence	289	2.18E-06	194
Grade IV High grade glioma cancer	Brain	Dependence	293	5.70E-05	173
Gliomatosis cerebri	Brain	Dependence	307	0.000197	94
Grade IV High grade glioma cancer	Brain	Withdrawal	329	0.000047	77
Glioma	Brain	Withdrawal	330	0.000063	98
Grade 3-4 Glioma cancer	Brain	Withdrawal	337	0.000266	82
Glioma cancer	Brain	Withdrawal	338	0.000305	94
Glioma cancer	Brain	Withdrawal	339	0.00052	92
Astrocytoma	Brain	Dependence	291	3.52E-06	181
Astrocytoma	Brain	Dependence	293	5.70E-06	173
Astrocytoma High grade	Brain	Dependence	294	5.95E-06	179
Astrocytoma	Brain	Dependence	294	6.45E-06	179
Astrocytoma High grade	Brain	Withdrawal	326	8.42E-06	82
Astrocytoma Grade IV	Brain	Withdrawal	329	4.77E-05	77
Astrocytoma	Brain	Withdrawal	330	5.12E-05	77
Breast / Colorectal cancer	Breast	Dependence	280	3.25E-08	233
Breast / Pancreatic cancer	Breast	Dependence	290	2.64E-06	181
Breast / Ovarian cancer	Breast	Dependence	304	0.000116	121
Breast cancer cell lines morphogenesis	Breast	Dependence	312	0.000573	3
Breast / Ovarian cancer	Breast	Dependence	315	0.000965	133
Breast cancer cell lines metastatic potential	Breast	Dependence	316	0.00131	3

Breast cancer cell lines cell proliferation	Breast	Withdrawal	344	0.00127	14
Breast / Gynaecological cancer	Breast	Withdrawal	346	0.00201	91
Breast / Pancreatic cancer	Breast	Withdrawal	355	0.00592	71
Breast cancer cell lines cell division	Breast	Withdrawal	358	0.00701	1
Philadelphia Chromosome Positive Chronic Myeloid Leukaemia	CML	Dependence	307	0.000213	5
Apoptosis Chronic Myeloid Leukaemia Cell Lines	CML	Dependence	319	0.00177	2
Refractory Chronic Myeloid Leukaemia	CML	Dependence	321	0.00196	4
Imatinib Resistant CML in Blast Crisis	CML	Withdrawal	347	0.00212	2
Imatinib Resistant CML	CML	Withdrawal	349	0.00308	2
Imatinib Resistant CML in Accelerated Phase	CML	Withdrawal	349	0.00308	2
Accelerated Phase CML	CML	Withdrawal	354	0.00521	3
Imatinib Resistant Philadelphia positive CML	CML	Withdrawal	356	0.00695	2
Colorectal / Breast cancer	Colorectal	Dependence	280	3.25E-08	233
Colon carcinoma	Colorectal	Dependence	264	7.27E-13	195
Colorectal carcinoma	Colorectal	Dependence	266	1.59E-12	201
Colon carcinoma	Colorectal	Dependence	267	2.04E-12	199
Colon carcinoma	Colorectal	Dependence	268	2.30E-12	197
Colon carcinoma	Colorectal	Dependence	268	2.84E-12	191
Colon carcinoma	Colorectal	Dependence	275	7.38E-11	213
Colon carcinoma	Colorectal	Dependence	278	1.95E-10	214
Colon carcinoma	Colorectal	Withdrawal	341	0.000617	74
Colon carcinoma	Colorectal	Withdrawal	341	0.000687	71
Colon carcinoma	Colorectal	Withdrawal	342	0.000748	70
Colon carcinoma	Colorectal	Withdrawal	342	0.000867	72
Colon carcinoma	Colorectal	Withdrawal	344	0.00129	72
Colon carcinoma	Colorectal	Withdrawal	352	0.00476	78
Rectum tumour	Colorectal	Dependence	311	0.00045	32
Rectum tumour	Colorectal	Dependence	318	0.00155	30

Rectum tumour	Colorectal	Dependence	320	0.00182	29
Rectum tumour	Colorectal	Withdrawal	348	0.00229	15
Gastroesophageal carcinoma	Esophagus	Dependence	262	3.15E-13	169
Gastroesophageal carcinoma	Esophagus	Dependence	265	1.53E-12	171
Gastroesophageal carcinoma	Esophagus	Dependence	282	9.77E-08	103
Esophageal adenocarcinoma	Esophagus	Dependence	320	0.00188	22
Gastroesophageal carcinoma	Esophagus	Withdrawal	331	0.000068	64
Esophageal carcinoma	Esophagus	Withdrawal	347	0.00202	15
Gastroesophageal adenocarcinoma	Esophagus	Withdrawal	354	0.00525	38
Renal clear cell carcinoma	Kidney	Dependence	315	0.00101	41
Upper renal tract carcinoma	Kidney	Dependence	324	0.00283	48
Liver carcinoma	Liver	Dependence	283	1.17E-08	168
Liver carcinoma	Liver	Dependence	287	2.52E-07	171
Liver carcinoma	Liver	Dependence	186	5.13E-06	186
Liver tumour	Liver	Dependence	292	5.13E-06	186
Hepatobiliary system cancer	Liver	Dependence	286	2.47E-07	179
Melanoma - cutaneous	Melanoma	Dependence	256	3.70E-15	281
Melanoma	Melanoma	Dependence	258	2.36E-14	298
Melanoma	Melanoma	Withdrawal	326	7.71E-06	110
Melanoma	Melanoma	Withdrawal	330	6.21E-05	115
Myeloma - cell lines cell death	Myeloma	Withdrawal	345	0.00159	5
Myeloma - cell lines apoptosis	Myeloma	Withdrawal	355	0.00622	4
Myeloma - cell lines cell adhesion	Myeloma	Withdrawal	357	0.00701	1
B-Cell Non-Hodgkin Lymphoma	NHL	Dependence	303	9.67E-05	45
Non-Hodgkin Lymphoma	NHL	Dependence	307	0.000208	55
Lymphoma - Hodgkins / DLBCNHL	NHL	Dependence	314	0.000777	3
B-Cell Non-Hodgkin Lymphoma	NHL	Withdrawal	354	0.0057	19
T-Lymphoblastic Leukaemia / Lymphoma	NHL	Dependence	300	4.08E-05	20

B-cell Lymphoma	NHL	Dependence	301	5.17E-05	48
Lymphoma	NHL	Dependence	303	0.000101	64
Diffuse Lymphoma	NHL	Dependence	313	0.000629	35
Diffuse B-cell Lymphoma	NHL	Dependence	315	0.000927	32
High Grade Lymphoma	NHL	Dependence	324	0.00269	20
B-Cell Non-Hodgkin Lymphoma Recurrent Genetic Abnormalities	NHL	Withdrawal	340	0.000611	5
B-Cell Lymphoblastic Lymphoma	NHL	Withdrawal	343	0.000891	4
T-Cell Lymphoblastic Leukaemia / Lymphoma	NHL	Withdrawal	348	0.00257	9
KMT2A-Rearrangement B-Cell Lymphoma	NHL	Withdrawal	353	0.00464	3
B-Cell Acute Lymphoblastic Lymphoma	NHL	Withdrawal	355	0.00646	3
Upper aerodigestive tract carcinoma	Oropharynx	Dependence	283	1.25E-07	97
Upper aerodigestive tract carcinoma	Oropharynx	Dependence	285	1.82E-06	98
Upper aerodigestive tract carcinoma	Oropharynx	Withdrawal	327	1.60E-05	44
Ovarian / Breast	Ovary	Dependence	304	0.000116	121
Ovarian / Breast	Ovary	Dependence	315	0.000965	133
Ovarian cancer	Ovary	Dependence	305	0.00014	68
Ovarian Tumour	Ovary	Dependence	308	0.000257	74
Ovarian cancer	Ovary	Dependence	312	0.000562	71
Ovarian adenocarcinoma	Ovary	Dependence	314	0.000792	62
Ovarian cancer	Ovary	Withdrawal	356	0.00701	1
Pancreatic / Breast cancer	Pancreas	Dependence	290	2.64E-06	181
Pancreatic / Breast cancer	Pancreas	Withdrawal	355	0.00592	71
Pancreatobiliary tumour	Pancreas	Dependence	288	9.10E-07	124
Pancreatic cancer	Pancreas	Dependence	297	1.19E-05	95
Pancreatic cancer	Pancreas	Dependence	298	1.26E-06	106
Pancreatic cancer	Pancreas	Dependence	299	0.000021	90
Pancreatic cancer	Pancreas	Dependence	300	0.000033	85
Pancreatic cancer	Pancreas	Dependence	301	0.00006	82

Pancreatic cancer cell lines cell migration	Pancreas	Dependence	323	0.00239	6
Pancreatic cancer cell lines cell invasion	Pancreas	Withdrawal	353	0.00521	3
Pancreatic cancer cell lines G1 phase	Pancreas	Withdrawal	355	0.0062	2
Pancreatic cancer	Pancreas	Withdrawal	356	0.00646	36
Protate cancer	Prostate	Dependence	279	2.88E-08	148
Prostatic adenocarcinoma	Prostate	Dependence	287	8.39E-07	123
Prostatic carcinoma	Prostate	Dependence	288	9.39E-07	128
Protate cancer	Prostate	Withdrawal	340	5.33E-04	59
Prostatic adenocarcinoma	Prostate	Withdrawal	350	0.00349	48
Prostatic carcinoma	Prostate	Withdrawal	50	0.00363	50
Prostate cancer cell adhesion	Prostate	Withdrawal	357	0.00701	1
Gastroesophageal carcinoma	Stomach	Dependence	262	3.15E-13	169
Gastroesophageal carcinoma	Stomach	Dependence	265	1.53E-12	171
Gastroesophageal carcinoma	Stomach	Dependence	282	9.77E-08	103
Gastroesophageal carcinoma	Stomach	Withdrawal	331	0.000068	64
Gastroesophageal adenocarcinoma	Stomach	Withdrawal	354	0.00525	38
Male Genital Neoplasm	Testis	Dependence	279	1.14E-08	153
Malignant Male Genital Neoplasm	Testis	Dependence	279	1.60E-08	151
Male Genital Neoplasm	Testis	Withdrawal	341	6.75E-04	60
Thyroid	Thyroid	Dependence	241	1.21E-17	318
Thyroid	Thyroid	Dependence	242	1.26E-17	319
Thyroid	Thyroid	Withdrawal	345	0.00141	115
Genitourinary adenocarcinoma	Urinary	Dependence	259	2.54E-14	259
Genitourinary adenocarcinoma	Urinary	Dependence	271	1.08E-11	277
Malignant genitourinary solid tumour	Urinary	Dependence	277	1.08E-10	277
Genitourinary adenocarcinoma	Urinary	Dependence	277	1.15E-10	277
Urinary tract cancer	Urinary	Dependence	320	0.00189	84
Morphology of urinary system	Urinary	Dependence	322	0.0022	17

Genitourinary adenocarcinoma	Urinary	Withdrawal	336	0.000216	97
Genitourinary adenocarcinoma	Urinary	Withdrawal	338	0.000322	359
Malignant genitourinary solid tumour	Urinary	Withdrawal	339	0.000466	111
Genitourinary tumour	Urinary	Withdrawal	340	0.000547	112

Supplementary Table S36.: P-Values for Cannabinoid-Cancer Association – Overall Schrott Data, Ordered by Minimum P-Value

Cancer	Number of Annotations	Median Gene Number	Cumulative Gene Number	Minimum P-Value	Mean P-Value	Median P-Value
Thyroid	3	318	752	1.21E-17	4.70E-04	1.26E-17
Melanoma	4	198	804	3.70E-15	1.75E-05	3.86E-06
Urinary	10	186	1870	2.54E-14	5.64E-04	2.69E-04
Stomach	5	103	545	3.15E-13	0.0011	9.77E-08
Esophagus	7	64	582	3.15E-13	0.0013	6.80E-05
Colorectal	18	76	2186	7.27E-13	8.38E-04	5.34E-04
Testis	3	151	364	1.14E-08	2.25E-04	1.60E-08
Liver	5	179	890	1.17E-08	2.15E-06	2.52E-07
Prostate	7	59	557	2.88E-08	0.0021	0.0005
Breast	10	81	851	3.25E-08	0.0019	0.0011
Brain	28	98	3726	5.33E-08	2.31E-04	4.74E-05
Oropharynx	3	97	239	1.25E-07	5.98E-06	1.82E-06
Pancreas	12	84	881	9.10E-07	0.0022	0.0000
NHL	15	20	365	4.08E-05	0.0018	0.0008
ALL	14	4.5	141	4.08E-05	0.0028	0.0028
Ovary	7	71	530	1.16E-04	0.0014	0.0006
CML	8	2	22	2.13E-04	0.0030	0.0026
AML	3	9	47	6.26E-04	0.0014	0.0009
Kidney	2	45	89	0.00101	0.0019	0.0019
Myeloma	3	4	10	0.00159	0.0049	0.0062

Supplementary Table S37.: P-Values for Cannabinoid-Cancer Association – Overall Schrott Data, Ordered by Median P-Value

Cancer	Number of Annotations	Median Gene Number	Cumulative Gene Number	Minimum P-Value	Mean P-Value	Median P-Value
Thyroid	3	318	752	1.21E-17	4.70E-04	1.26E-17
Testis	3	151	364	1.14E-08	2.25E-04	1.60E-08
Stomach	5	103	545	3.15E-13	0.0011	9.77E-08
Liver	5	179	890	1.17E-08	2.15E-06	2.52E-07
Oropharynx	3	97	239	1.25E-07	5.98E-06	1.82E-06
Melanoma	4	198	804	3.70E-15	1.75E-05	3.86E-06
Pancreas	12	83.5	881	9.10E-07	0.0022	0.0000
Brain	28	98	3726	5.33E-08	2.31E-04	4.74E-05
Esophagus	7	64	582	3.15E-13	0.0013	6.80E-05
Urinary	10	185.5	1870	2.54E-14	5.64E-04	2.69E-04
Prostate	7	59	557	2.88E-08	0.0021	0.0005
Colorectal	18	76	2186	7.27E-13	8.38E-04	5.34E-04
Ovary	7	71	530	1.16E-04	0.0014	0.0006
NHL	15	20	365	4.08E-05	0.0018	0.0008
AML	3	9	47	6.26E-04	0.0014	0.0009
Breast	10	81	851	3.25E-08	0.0019	0.0011
Kidney	2	44.5	89	0.0010	0.0019	0.0019
CML	8	2	22	2.13E-04	0.0030	0.0026
ALL	14	4.5	141	4.08E-05	0.0028	0.0028
Myeloma	3	4	10	0.0016	0.0049	0.0062

Supplementary Table S38.: P-Values for Cannabinoid-Cancer Association – Cannabis Dependency, Schrott Data, Ordered by Minimum P-Value

Cancer	Number of Annotations	Median Gene Number	Cumulative Gene Number	Minimum P-Value	Mean P-Value	Median P-Value
Thyroid	6	115	637	1.21E-17	1.24E-17	1.24E-17
Melanoma	8	112.5	579	3.70E-15	1.36E-14	1.36E-14
Urinary	60	111.5	1191	2.54E-14	0.0007	1.12E-10
Esophagus	28	38	465	3.15E-13	4.70E-04	4.89E-08
Stomach	15	51	443	3.15E-13	3.26E-08	1.53E-12
Colorectal	198	72	1734	7.27E-13	3.47E-04	7.38E-11
Testis	6	60	304	1.14E-08	1.37E-08	1.37E-08
Liver	25	-	890	1.17E-08	2.15E-06	2.52E-07
Prostate	21	49	399	2.88E-08	6.02E-07	8.39E-07
Breast	60	42.5	674	3.25E-08	4.94E-04	3.45E-04
Brain	448	82	2779	5.33E-08	1.44E-04	5.82E-06
Oropharynx	6	44	195	1.25E-07	9.73E-07	9.73E-07
Pancreas	96	19.5	769	9.10E-07	3.15E-04	1.65E-05
ALL	28	4.5	23	4.08E-05	2.23E-04	2.23E-04
NHL	135	4.5	322	4.08E-05	6.13E-04	2.08E-04
Ovary	42	1	529	1.16E-04	4.72E-04	4.10E-04
CML	24	2	11	2.13E-04	0.0013	0.0018
AML	6	36	11	8.96E-04	0.0017	0.0017
Kidney	4	-	89	0.0010	0.0019	0.0019

Supplementary Table S39.: P-Values for Cannabinoid-Cancer Association – Cannabis Dependency, Schrott Data, Ordered by Median P-Value

Cancer	Number of Annotations	Median Gene Number	Cumulative Gene Number	Minimum P-Value	Mean P-Value	Median P-Value
Thyroid	6	115	637	1.21E-17	1.24E-17	1.24E-17
Melanoma	8	112.5	579	3.70E-15	1.36E-14	1.36E-14
Stomach	15	51	443	3.15E-13	3.26E-08	1.53E-12
Colorectal	198	72	1734	7.27E-13	3.47E-04	7.38E-11
Urinary	60	111.5	1191	2.54E-14	0.0007	1.12E-10
Testis	6	60	304	1.14E-08	1.37E-08	1.37E-08
Esophagus	28	38	465	3.15E-13	4.70E-04	4.89E-08
Liver	25	-	890	1.17E-08	2.15E-06	2.52E-07
Prostate	21	49	399	2.88E-08	6.02E-07	8.39E-07
Oropharynx	6	44	195	1.25E-07	9.73E-07	9.73E-07
Brain	448	82	2779	5.33E-08	1.44E-04	5.82E-06
Pancreas	96	19.5	769	9.10E-07	3.15E-04	1.65E-05
NHL	135	4.5	322	4.08E-05	6.13E-04	2.08E-04
ALL	28	4.5	23	4.08E-05	2.23E-04	2.23E-04
Breast	60	42.5	674	3.25E-08	4.94E-04	3.45E-04
Ovary	42	1	529	1.16E-04	4.72E-04	4.10E-04
AML	6	36	11	8.96E-04	0.0017	0.0017
CML	24	2	11	2.13E-04	0.0013	0.0018
Kidney	4	-	89	0.0010	0.0019	0.0019

Supplementary Table S40.: P-Values for Cannabinoid-Cancer Association – Cannabis Withdrawal, Schrott Data, Ordered by Minimum P-Value

Cancer	Number of Annotations	Median Gene Number	Cumulative Gene Number	Minimum P-Value	Mean P-Value	Median P-Value
Melanoma	8	112.5	225	7.71E-06	3.49E-05	3.49E-05
Brain	336	82	947	8.42E-06	3.48E-04	1.60E-04
Oropharynx	3	44	44	1.60E-05	1.60E-05	1.60E-05
Esophagus	21	38	117	6.80E-05	0.0024	0.0020
Stomach	10	51	102	6.80E-05	0.0027	0.0027
Urinary	40	111.5	679	2.16E-04	3.88E-04	3.94E-04
Prostate	28	49	158	5.33E-04	0.0037	0.0036
ALL	168	4.5	118	6.01E-04	0.0032	0.0034
NHL	90	4.5	43	6.11E-04	0.0035	0.0036
Colorectal	126	72	452	6.17E-04	0.0016	8.67E-04
AML	3	36	36	6.26E-04	6.26E-04	6.26E-04
Testis	3	60	60	6.75E-04	6.75E-04	6.75E-04
Breast	40	42.5	177	0.00127	0.0041	0.0040
Thyroid	3	115	115	0.00141	0.0014	0.0014
Myeloma	9	4	10	0.00159	0.0049	0.0062
CML	40	2	11	0.00212	0.0041	0.0031
Pancreas	48	19.5	112	0.00521	0.0059	0.0061
Ovary	7	1	1	0.00701	0.0070	0.0070

Supplementary Table S41.: P-Values for Cannabinoid-Cancer Association – Cannabis Withdrawal, Schrott Data, Ordered by Median P-Value

Cancer	Number of Annotations	Median Gene Number	Cumulative Gene Number	Minimum P-Value	Mean P-Value	Median P-Value
Oropharynx	3	44	44	1.60E-05	1.60E-05	1.60E-05
Melanoma	8	112.5	225	7.71E-06	3.49E-05	3.49E-05
Brain	336	82	947	8.42E-06	3.48E-04	1.60E-04
Urinary	40	111.5	679	2.16E-04	3.88E-04	3.94E-04
AML	3	36	36	6.26E-04	6.26E-04	6.26E-04
Testis	3	60	60	6.75E-04	6.75E-04	6.75E-04
Colorectal	126	72	452	6.17E-04	0.0016	8.67E-04
Thyroid	3	115	115	0.00141	0.0014	0.0014
Esophagus	21	38	117	6.80E-05	0.0024	0.0020
Stomach	10	51	102	6.80E-05	0.0027	0.0027
CML	40	2	11	0.00212	0.0041	0.0031
ALL	168	4.5	118	6.01E-04	0.0032	0.0034
Prostate	28	49	158	5.33E-04	0.0037	0.0036
NHL	90	4.5	43	6.11E-04	0.0035	0.0036
Breast	40	42.5	177	0.00127	0.0041	0.0040
Pancreas	48	19.5	112	0.00521	0.0059	0.0061
Myeloma	9	4	10	0.00159	0.0049	0.0062
Ovary	7	1	1	0.00701	0.0070	0.0070

Supplementary Table S42.: Contrast of Cannabis Dependence and Withdrawal Significance Levels and Gene Numbers, Schrott Data

Cancer	Minimum P-Value Dependence	Minimum P-Value Withdrawal	P-Value Ratio	Total Gene Number Dependence	Total Gene Number Withdrawal	Gene Number Ratio
Ovary	1.16E-04	0.0070	60.43	529	1	529.00
NHL	4.08E-05	6.11E-04	14.98	322	43	7.49
Pancreas	9.10E-07	0.0052	5.73E+03	769	112	6.87
Thyroid	1.21E-17	0.0014	1.17E+14	637	115	5.54
Testis	1.14E-08	6.75E-04	5.92E+04	304	60	5.07
Oropharynx	1.25E-07	1.60E-05	128.00	195	44	4.43
Stomach	3.15E-13	6.80E-05	2.16E+08	443	102	4.34
Esophagus	3.15E-13	6.80E-05	2.16E+08	465	117	3.97
Colorectal	7.27E-13	6.17E-04	8.49E+08	1734	452	3.84
Breast	3.25E-08	0.0013	3.91E+04	674	177	3.81
Brain	5.33E-08	8.42E-06	157.97	2779	947	2.93
Melanoma	3.70E-15	7.71E-06	2.08E+09	579	225	2.57
Prostate	2.88E-08	5.33E-04	1.85E+04	399	158	2.53
Urinary	2.54E-14	2.16E-04	8.50E+09	1191	679	1.75
CML	2.13E-04	0.0021	9.95	11	11	1.00
AML	8.96E-04	6.26E-04	0.70	11	36	0.31
ALL	4.08E-05	6.01E-04	14.73	23	118	0.19
Liver	1.17E-08	NA	NA	890	NA	NA
Kidney	0.00101	NA	NA	89	NA	NA
Myeloma	NA	0.0016	NA	NA	10	NA

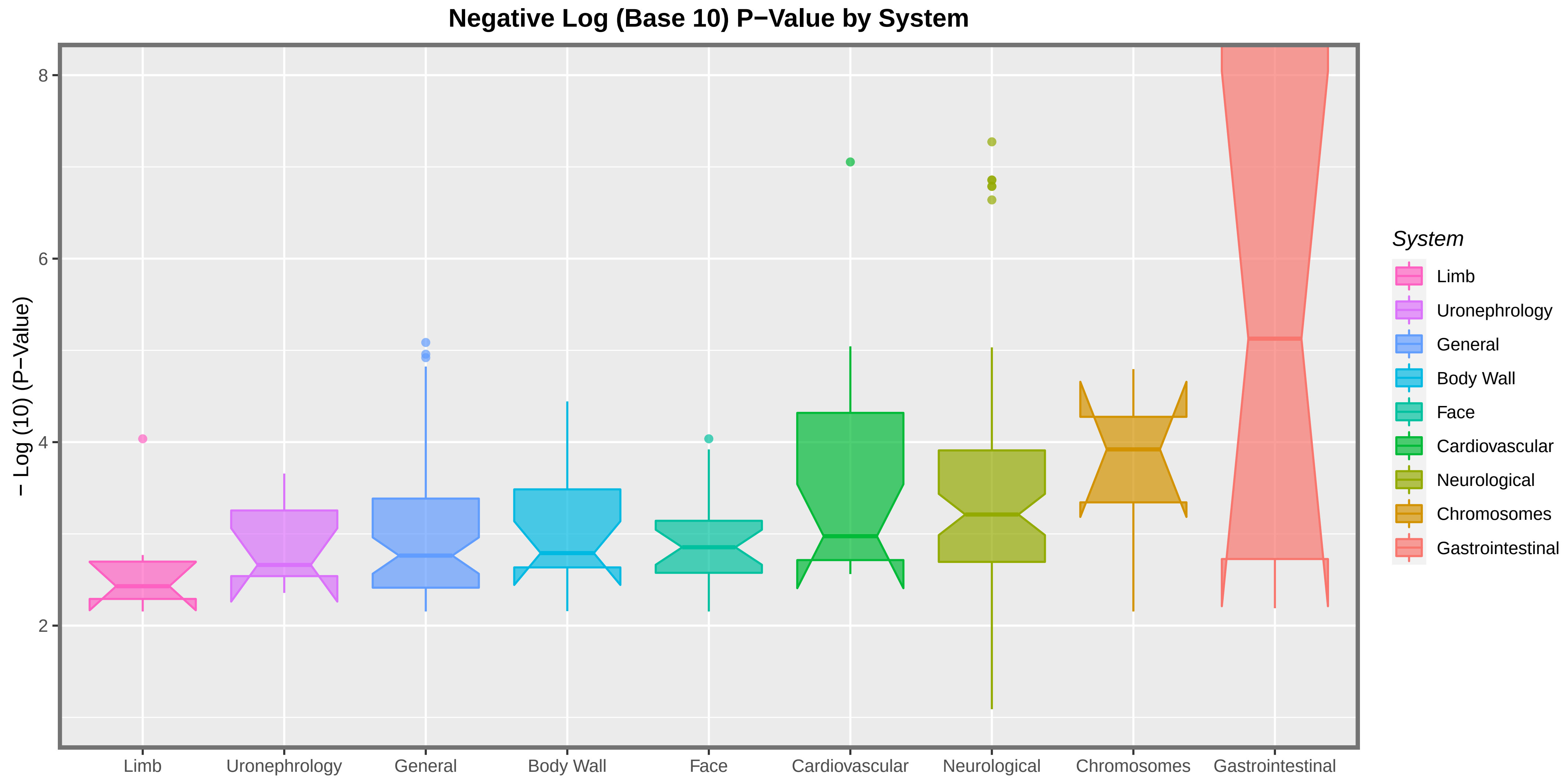
Negative Log (Base 10) P−Value by System
− Log (10) (P−Value)
8
6
4
2
Limb
Uronephrology
General
Body Wall
Face
Cardiovascular
Neurological
Chromosomes
Gastrointestinal
System
Limb
Uronephrology
General
Body Wall
Face
Cardiovascular
Neurological
Chromosomes
Gastrointestinal

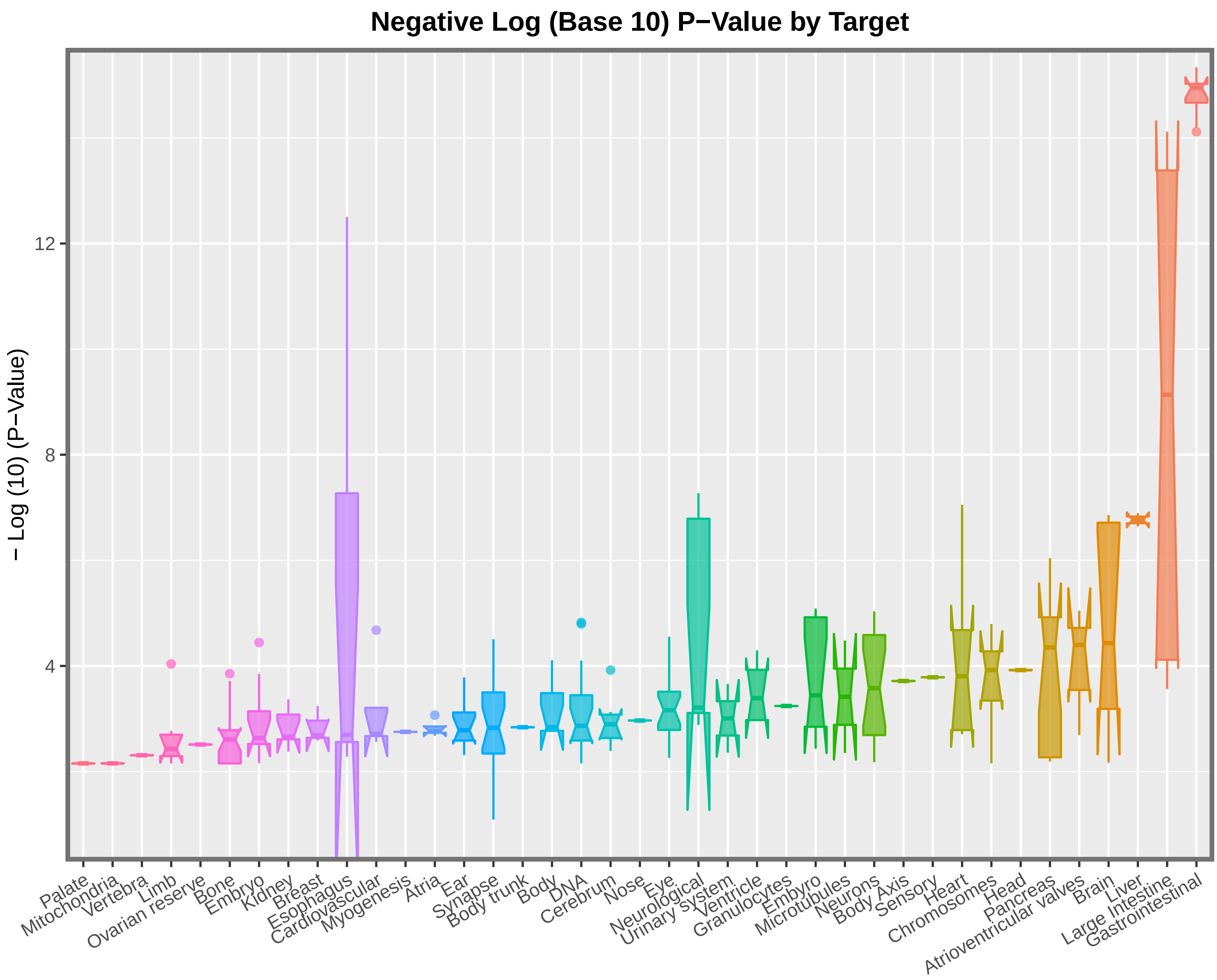

Negative Log (Base 10) P−Value by Target
− Log (10) (P−Value)
12
8
4
Palate
Mitochondria
Vertebra
Limb
Ovarian reserve
Bone
Embryo
Kidney
Breast
Esophagus
Cardiovascular
Myogenesis
Atria
Ear
Synapse
Body trunk
Body
DNA
Cerebrum
Nose
Eye
Neurological
Urinary system
Ventricle
Granulocytes
Embryo
Microtubules
Neurons
Body Axis
Sensory
Heart
Chromosomes
Head
Pancreas
Atrioventricular valves
Brain
Liver
Large Intestine
Gastrointestinal

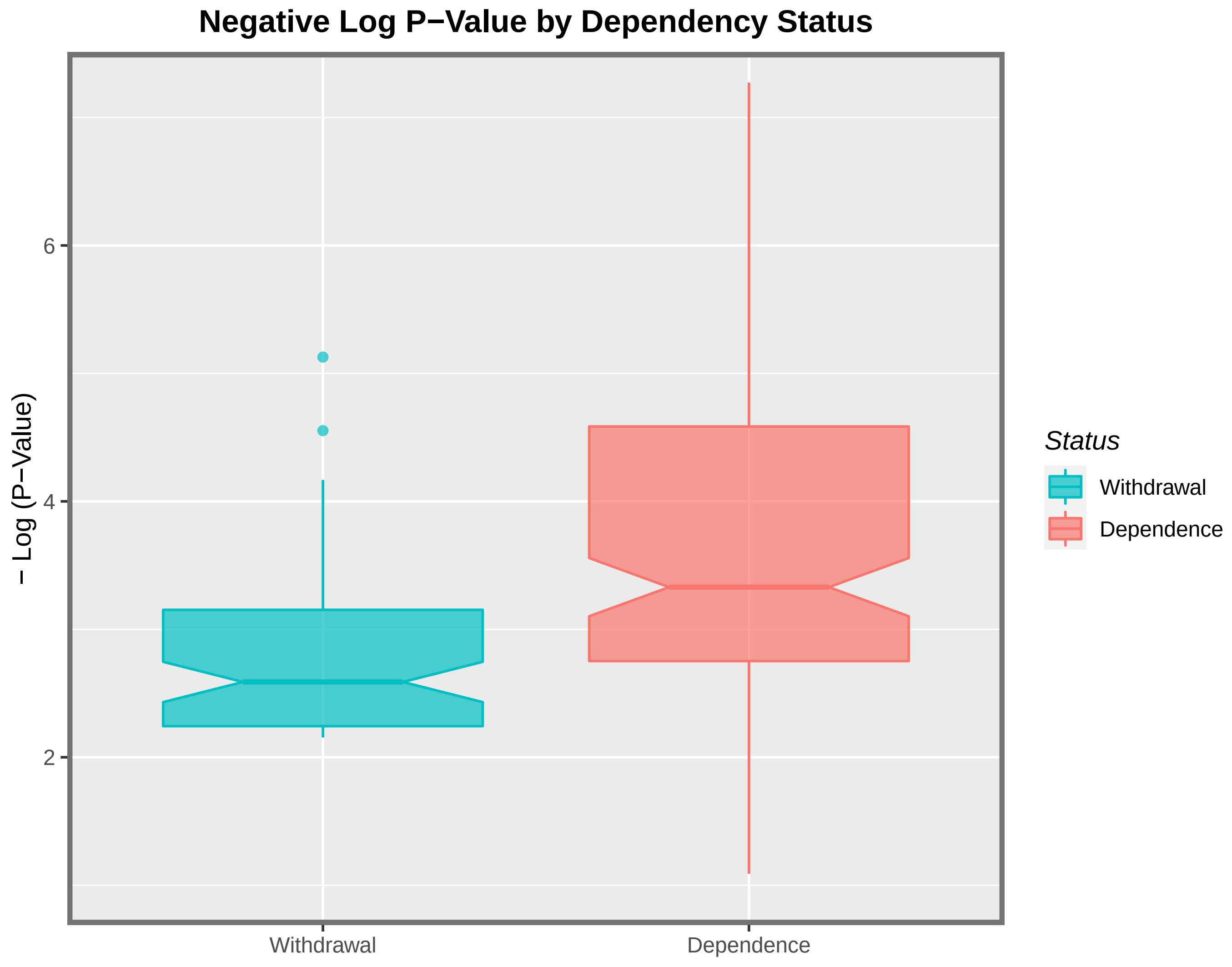

Negative Log P−Value by Dependency Status
− Log (P−Value)
6
4
2
Withdrawal
Dependence
Status
Withdrawal
Dependence

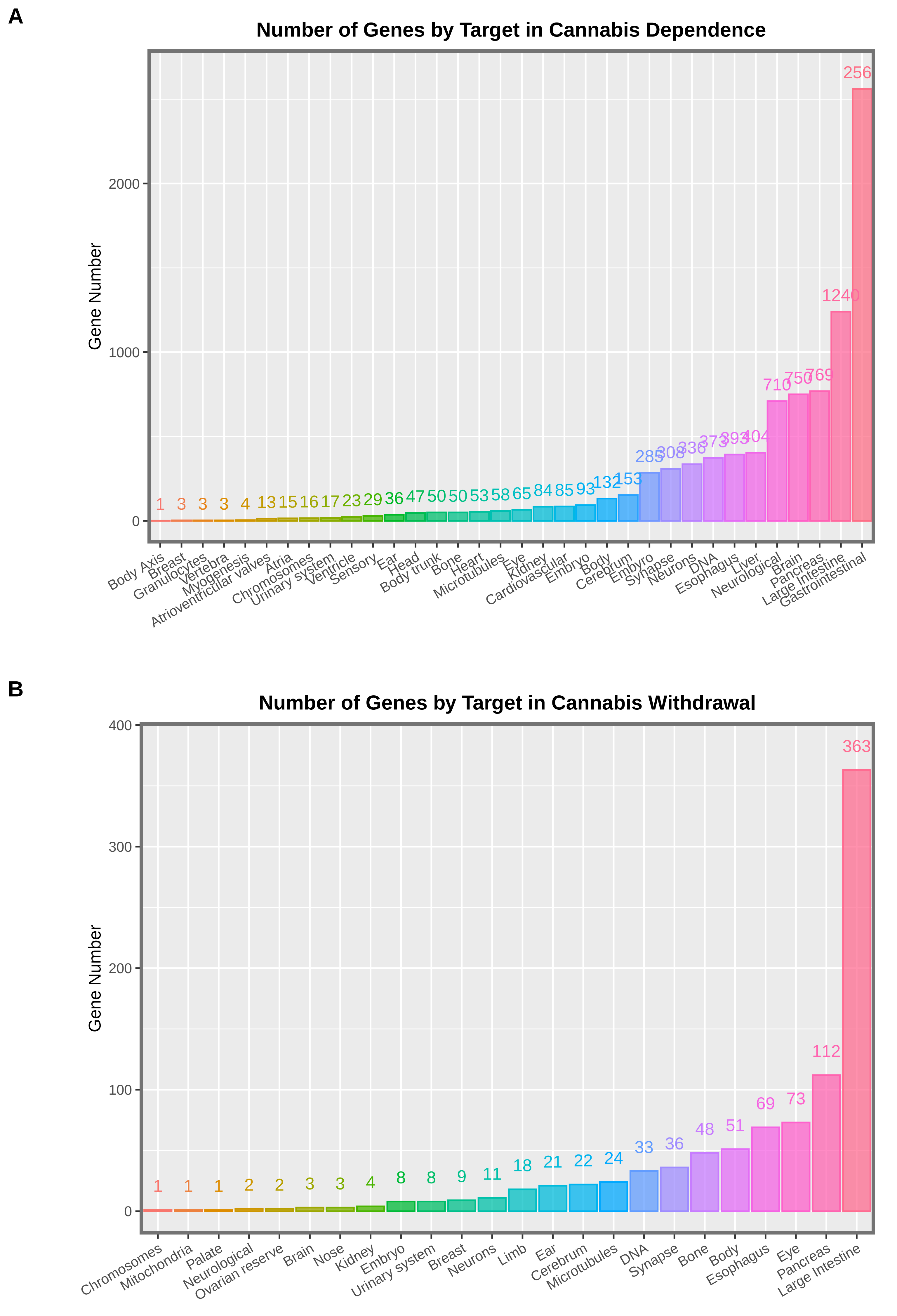
A
Number of Genes by Target in Cannabis Dependence
Gene Number
0
1000
2000
1 3 3 3 4 13 15 16 17 23 29 36 47 50 50 53 58 65 84 85 93 132 153 285 308 336 373 393 404 710 750 769 1240 256
Body Axis
Breast
Granulocytes
Vertebra
Myogenesis
Atrioventricular valves
Atria
Chromosomes
Urinary system
Ventricle
Sensory
Ear
Head
Body trunk
Bone
Heart
Microtubules
Eye
Kidney
Cardiovascular
Embryo
Body
Cerebrum
Embryo
Synapse
Neurons
DNA
Esophagus
Liver
Neurological
Brain
Pancreas
Large Intestine
Gastrointestinal
B
Number of Genes by Target in Cannabis Withdrawal
Gene Number
0
100
200
300
400
1 1 1 2 2 3 3 4 8 8 9 11 18 21 22 24 33 36 48 51 69 73 112 363
Chromosomes
Mitochondria
Palate
Neurological
Ovarian reserve
Brain
Nose
Kidney
Embryo
Urinary system
Breast
Neurons
Limb
Ear
Cerebrum
Microtubules
DNA
Synapse
Bone
Body
Esophagus
Eye
Pancreas
Large Intestine

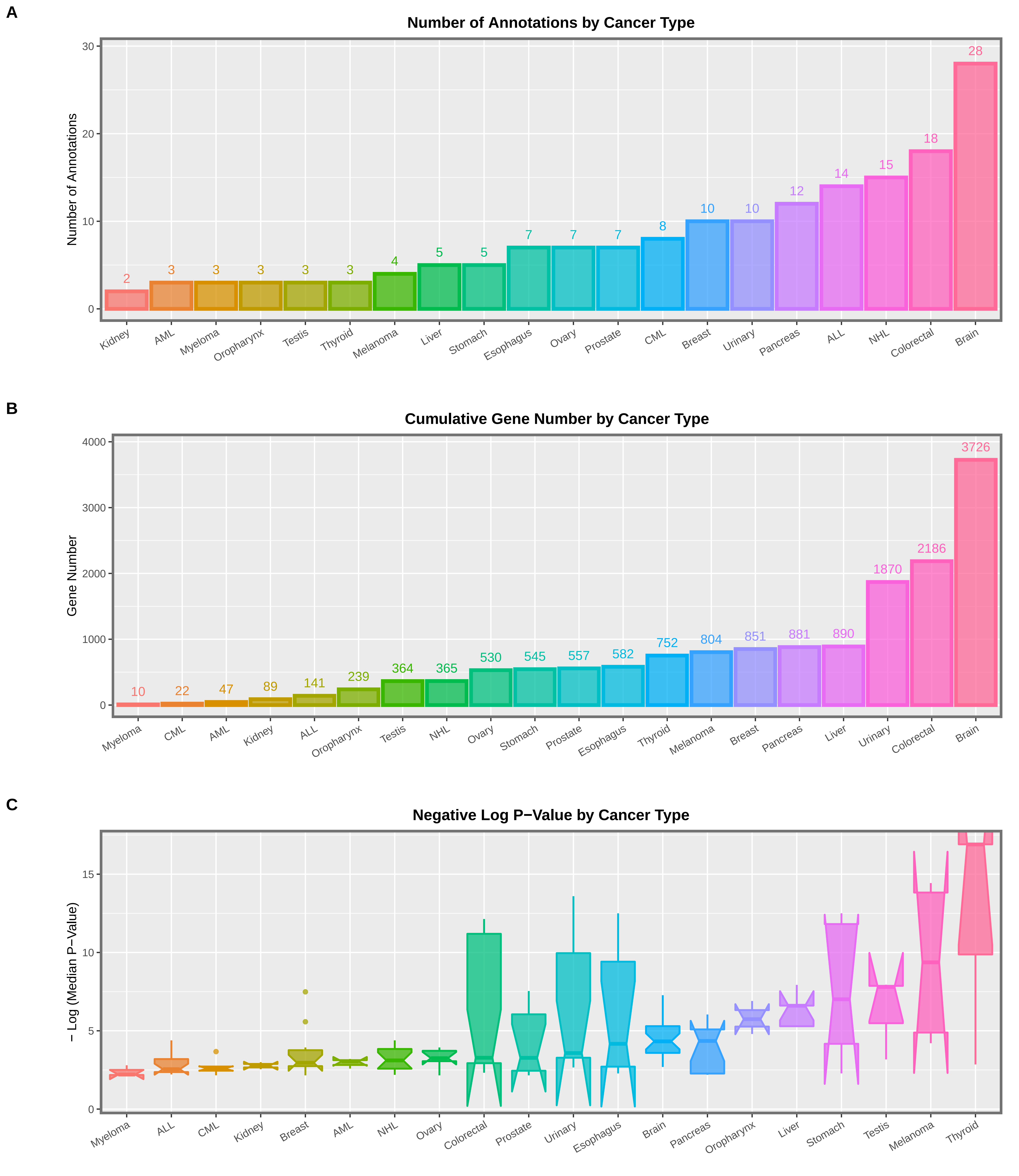
A
Number of Annotations by Cancer Type
Number of Annotations
Kidney 2, AML 3, Myeloma 3, Oropharynx 3, Testis 3, Thyroid 3, Melanoma 4, Liver 5, Stomach 5, Esophagus 7, Ovary 7, Prostate 7, CML 8, Breast 10, Urinary 10, Pancreas 12, ALL 14, NHL 15, Colorectal 18, Brain 28
B
Cumulative Gene Number by Cancer Type
Gene Number
Myeloma 10, CML 22, AML 47, Kidney 89, ALL 141, Oropharynx 239, Testis 364, NHL 365, Ovary 530, Stomach 545, Prostate 557, Esophagus 582, Thyroid 752, Melanoma 804, Breast 851, Pancreas 881, Liver 890, Urinary 1870, Colorectal 2186, Brain 3726
C
Negative Log P−Value by Cancer Type
− Log (Median P−Value)
Myeloma, ALL, CML, Kidney, Breast, AML, NHL, Ovary, Colorectal, Prostate, Urinary, Esophagus, Brain, Pancreas, Oropharynx, Liver, Stomach, Testis, Melanoma, Thyroid

A

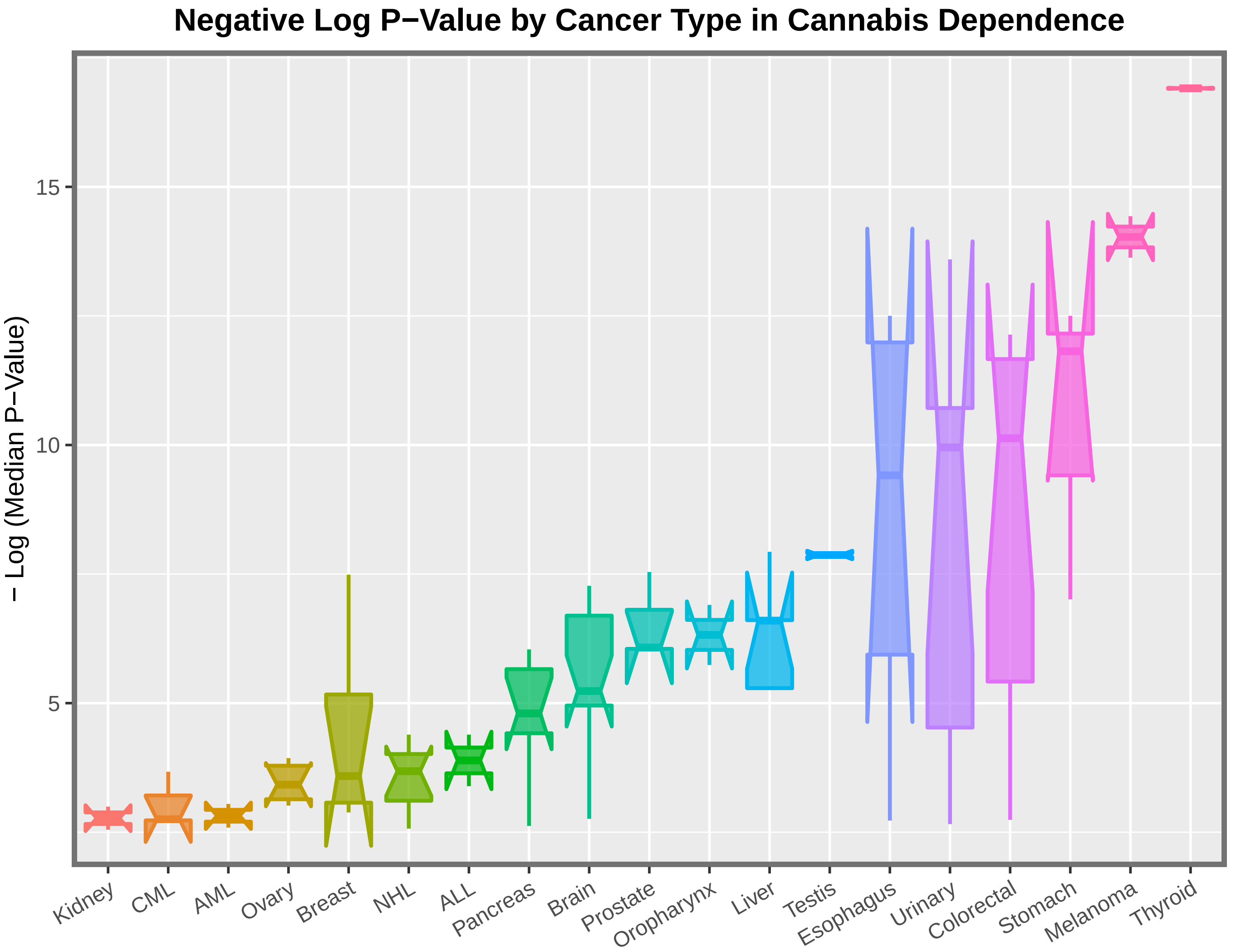
Negative Log P−Value by Cancer Type in Cannabis Dependence
− Log (Median P−Value)
15
10
5
Kidney
CML
AML
Ovary
Breast
NHL
ALL
Pancreas
Brain
Prostate
Oropharynx
Liver
Testis
Esophagus
Urinary
Colorectal
Stomach
Melanoma
Thyroid

B

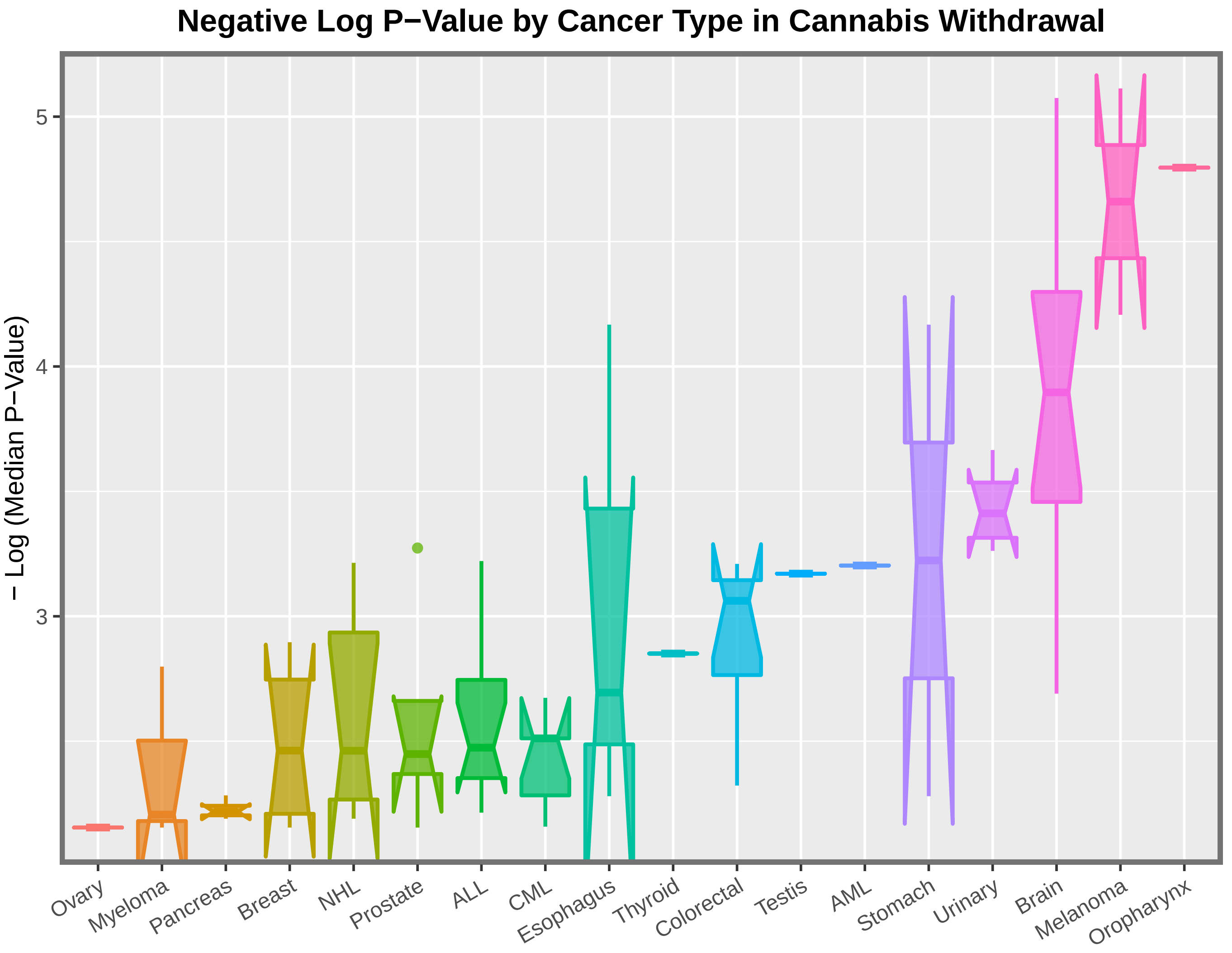
Negative Log P−Value by Cancer Type in Cannabis Withdrawal
− Log (Median P−Value)
5
4
3
Ovary
Myeloma
Pancreas
Breast
NHL
Prostate
ALL
CML
Esophagus
Thyroid
Colorectal
Testis
AML
Stomach
Urinary
Brain
Melanoma
Oropharynx

Supplementary Figure Captions

Supplementary Figure S1. Overall results - negative logarithm of P-Values for congenital anomalies from Schrott Database by organ system.

Supplementary Figure S2. Overall results - negative logarithm of P-Values for congenital anomalies from Schrott Database by organ target.

Supplementary Figure S3. Overall results – Boxplot of negative logarithm of grouped P-Values for congenital anomalies from Schrott Database comparing cannabis dependency with cannabis withdrawal.

Supplementary Figure S4. Number of genes annotated in the Schrott database for target organs by dependency status in (A) cannabis dependence and (B) withdrawal.

Supplementary Figure S5. (A) Numbers of gene annotations, (B) numbers of genes affected and (C) negative logarithm of P-value by cancer type - overall Schrott data.

Supplementary Figure S6. Direct comparison between P-values for cannabis-cancer relationships between (A) cannabis dependence and (B) cannabis withdrawal, Schrott data.
